# Hair regrowth treatment efficacy and resistance in androgenetic alopecia: A systematic review and continuous Bayesian network meta-analysis

**DOI:** 10.3389/fmed.2022.998623

**Published:** 2023-01-23

**Authors:** Peter R. Feldman, Pietro Gentile, Charles Piwko, Hendrik M. Motswaledi, Samantha Gorun, Jacob Pesachov, Michael Markel, Maxwell I. Silver, Megan Brenkel, Oriel J. Feldman, Corey L. Kamen, Elizabeth Uleryk, Jaime Guevara-Aguirre, Klaus M. Fiebig

**Affiliations:** ^1^Arbor Life Labs, Toronto, ON, Canada; ^2^Norwich Medical School, University of East Anglia, Norwich, United Kingdom; ^3^Surgical Science Department, University of Rome Tor Vergata, Rome, Italy; ^4^CHP Pharma Inc., Thornhill, ON, Canada; ^5^Department of Dermatology, Sefako Makgatho Health Sciences University, Pretoria, South Africa; ^6^Faculty of Epidemiology and Biostatistics, Western University, London, ON, Canada; ^7^School or Mathematics and Statistics, University of Glasgow, Glasgow, United Kingdom; ^8^Faculty of Medicine, Technion-Israel Institute of Technology, Haifa, Israel; ^9^Faculty of Dentistry, University of Toronto, Toronto, ON, Canada; ^10^Faculty of Medicine and Dentistry, University of Alberta, Edmonton, AB, Canada; ^11^Faculty of Medicine, University of Ottawa, Ottawa, ON, Canada; ^12^Faculty of Science, Wilfrid Laurier University, Waterloo, ON, Canada; ^13^Uleryk Consulting, Mississauga, ON, Canada; ^14^College of Medicine, Universidad San Francisco De Quito (USFQ), Quito, Ecuador; ^15^Faculty of Health, Medicine and Life Sciences, Maastricht University, Maastricht, Netherlands; ^16^Institute of Endocrinology, Metabolism, and Reproduction (IEMYR), Quito, Ecuador; ^17^College of Medicine, University of Florida, Gainesville, FL, United States

**Keywords:** hair loss, ALRV5XR, Dutasteride, Finasteride, LLLT, Minoxidil, Viviscal, Nutrafol

## Abstract

**Background:**

Androgenetic alopecia (AGA) affects almost half the population, and several treatments intending to regenerate a normal scalp hair phenotype are used. This is the first study comparing treatment efficacy response and resistance using standardized continuous outcomes.

**Objective:**

To systematically compare the relative efficacy of treatments used for terminal hair (TH) regrowth in women and men with AGA.

**Methods:**

A systematic literature review was conducted (from inception to August 11, 2021) to identify randomized, Placebo-controlled trials with ≥ 20 patients and reporting changes in TH density after 24 weeks. Efficacy was analyzed by sex at 12 and 24 weeks using Bayesian network meta-analysis (B-NMA) and compared to frequentist and continuous outcomes profiles.

**Results:**

The search identified 2,314 unique articles. Ninety-eight were included for full-text review, and 17 articles met the inclusion criteria for data extraction and analyses. Eligible treatments included ALRV5XR, Dutasteride 0.5 mg/day, Finasteride 1 mg/day, low-level laser comb treatment (LLLT), Minoxidil 2% and 5%, Nutrafol, and Viviscal. At 24 weeks, the B-NMA regrowth efficacy in TH/cm^2^ and significance (^**^) in women were ALRV5XR: 30.09^**^, LLLT: 16.62^**^, Minoxidil 2%: 12.13^**^, Minoxidil 5%: 10.82^**^, and Nutrafol: 7.32^**^, and in men; ALRV5XR: 21.03^**^, LLLT: 18.75^**^, Dutasteride: 18.37^**^, Viviscal: 13.23, Minoxidil 5%: 13.13^**^, Finasteride: 12.38, and Minoxidil 2%: 10.54. Two distinct TH regrowth response profiles were found; Continuous: ALRV5XR regrowth rates were linear in men and accelerated in women; Resistant: after 12 weeks, LLLT, Nutrafol, and Viviscal regrowth rates attenuated while Dutasteride and Finasteride plateaued; Minoxidil 2% and 5% lost some regrowth. There were no statistical differences for the same treatment between women and men. B-NMA provided more accurate, statistically relevant, and conservative results than the frequentist-NMA.

**Conclusion:**

Some TH regrowth can be expected from most AGA treatments with less variability in women than men. Responses to drug treatments were rapid, showing strong early efficacy followed by the greatest resistance effects from flatlining to loss of regrowth after 12–16 weeks. Finasteride, Minoxidil 2% and Viviscal in men were not statistically different from Placebo. LLLT appeared more efficacious than pharmaceuticals. The natural product formulation ALRV5XR showed better efficacy in all tested parameters without signs of treatment resistance (see Graphical abstract).

**Systematic review registration:**

www.crd.york.ac.uk/PROSPERO/display_record.asp?ID=CRD42021268040, identifier CRD42021268040.

## Graphical Abstract

Hair regrowth treatment efficacy and resistance in androgenetic alopecia. Systematic literature review and Bayesian network meta-analysis of terminal hair regrowth efficacy and resistance in women and men with androgenetic alopecia using different treatments vs. Placebo at 12 and 24 weeks. Women and men are listed in descending order of efficacy at 24 weeks of network meta-analysis (NMA) after 250,000 Markov Chain Monte Carlo simulations. Regrowth measures efficacy as mean difference (MD) in TH/cm^2^ between each treatment and Placebo using direct and indirect comparisons. 12 and 24W refer to 12 and 24 week treatment duration. SUCRA: surface under the cumulative ranking area measures relative confidence. Response Profile is a line graph connecting the treatment changes from baseline (BL) to MD at 12 and 24W. Y-axes (MD) are scaled 0–31 for women and 0–22 for men. Response Ratio (%) is change in regrowth of 12–24W compared to BL-12W. Quality of evidence is CiNeMA results using Cochrane risk of bias, GRADE scores, and study data. Heat maps are shared between each sex’s numerical result block and manually assigned to categorical results. 
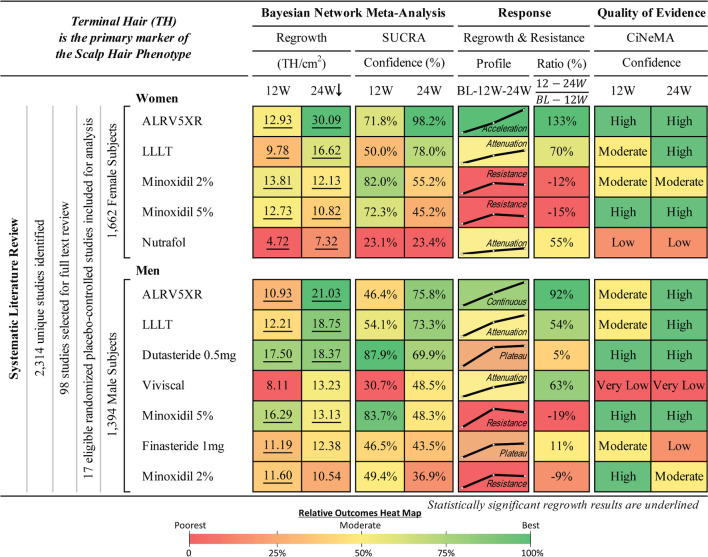

## 1. Introduction

Androgenetic alopecia (AGA), also known as male or female pattern hair loss, is the most common form of hair loss in men and women (1). AGA is a polygenic, heritable, age-dependent process that results in a progressive, non-scarring decline in scalp hair density, and is characterized by gradual loss of terminal hair (TH) and follicular miniaturization to vellus-like hair fibers in a generally sex-dependent pattern, leading to the eventual irreversible loss of functional potential of hair follicles (HFs) (1–3). THs are large pigmented hairs with a shaft diameter of 30–150 μm, while vellus hairs (VHs) have less than 30 μm diameter, are short (< 30 mm), unpigmented, and barely visible (4–6). A normal scalp averages seven THs per each VH (4). AGA results in a reduction in total hair count and a TH/VH ratio that is clinically identifiable when 15–30% of hair is lost or when the TH/VH ratio approaches 4 to 1 (1, 5, 7–9). TH contributes to more than 85% of total hair density, and constitutes almost all visible scalp hair. In consequence, its count is the single most important marker of the normal scalp hair phenotype.

Identifying and counting TH can be challenging. Manual hair counting methods used prior to 1990 were inaccurate (10). While phototrichoscopies of unclipped and undyed hair are useful for patient diagnosis and follow-up in the clinic, they are not precise and can lead to miscount. The 4 mm punch biopsy is highly effective at measuring and counting hair; however, few clinical trials have used this methodology. Of relevance, results from biopsies can anticipate, by weeks, visible scalp hair regrowth. Notably, in the last 30 years, improvements in imaging technology have increased the accuracy of hair diameter measurements and TH counting reliability. These newer methods are accurate, repeatable, and cost-effective. Among them, the contrast-enhanced phototrichogram (CEPT) technique, performed on hair clipped to < 1 mm and dyed, generates highly precise results. Consequently, CEPT linked to specialized analysis software is the currently accepted gold standard for measuring hair regrowth in clinical trials (5, 8, 9).

At the present, first-line treatments for AGA include the type I 5α-reductase androgen inhibitor, oral Finasteride 1 mg/day for men, and the potassium channel opener, topical Minoxidil 2% and 5%, for both sexes (1, 5, 8, 9, 11). TH regrowth induced by these agents was found to be derived from activation of dormant telogen terminal follicles (5, 8, 9). In any case, it has been noted that AGA-miniaturized vellus-like hairs, are not definitively influenced by these drugs; consequently, they cannot be converted back to THs (5, 8, 9). In contrast, ALRV5XR, a novel botanical natural product treatment for AGA, showed significant TH regrowth and appeared to reverse miniaturized hairs (12, 13). Individuals affected by AGA seeking therapy would benefit from both a safe option aimed to prevent further hair loss and, potentially, a treatment that might regenerate their normal scalp hair phenotype.

There have been several double-blinded randomized Placebo-controlled trials (RCTs) that lasted at least 24 weeks. This timeframe appears appropriate since it allows for reasonable measurement of continuous efficacy parameters. In addition, this period permits midpoint evaluations at 12 weeks to detect earlier changes. Therefore, a network meta-analysis (NMA) performed at 12 and 24 week time points, and an analysis of continuous treatment outcomes could better allow for a proper comparison of the regrowth profiles seen in different trials. They will also permit a more precise evaluation of discrete responses as well as of resistance dynamics.

Hair regrowth outcomes for AGA treatments have been compared in 15 meta-analyses (14–28). These evaluations included studies that performed total hair counting and other non-comparable hair counting methodologies. Notably, they compared a single outcome endpoint for treatments of different duration, including non-RCTs. In summary, among these 15 meta-analyses, which included studies with inaccurate hair counting methods and non-RCTs, only one, performed in men, compared the TH outcome of three drugs.

To our knowledge, no published NMA study, in women or men, has compared the efficacy or resistance of all commercially available non-surgical AGA treatments using standardized outcomes such as determination, at precise times, of TH characteristics, or with assessment of hair density by unit area and evaluation of continuous efficacy profiles over the study period. Furthermore, there are no reports of NMAs comparing pharmaceuticals, medical devices and natural treatments, or Bayesian to frequentist outcomes, or studies that measure efficacy using standardized methods for the treatment of AGA.

Under the above cited premises, the objective of this study was to examine, in women and men, the relative 24 week efficacy of all commercially available AGA treatments using standard dosage and administration routes. Efficacy was derived from determining the outcome of the most appropriate parameter: TH regrowth per unit area over an identical treatment duration period. In addition, treatment outcome was followed over time aiming to determine treatment resistance effects and the continuous efficacy of each treatment in maintaining or improving an existing phenotype.

## 2. Methods

### 2.1. Study eligibility

A professional librarian performed a database search on MEDLINE, Medline-in-Process, Medline ePub Ahead of Print, EMBASE databases (OvidSP) and Cochrane, initially on July 8, 2020, without date restrictions. The search was updated on August 11, 2021. Both subject headings and text word terms were used to search for RCTs on alopecia and current standard of care therapies (e.g., Minoxidil or Finasteride or Dutasteride or biotin or low-level laser light therapy or supplements or transplant, etc.). Manual and gray literature searches were also conducted, and studies included in prior meta-analyses were evaluated for study eligibility (see Search Strategy in Supplementary Appendix 2).

Eligible studies included RCTs of men or women diagnosed with AGA using either an approved or off-label drug, device, or commercially relevant treatment such as platelet-rich plasma (PRP) and natural products for a duration of at least 24 weeks, and which measured density changes of scalp TH, or non-vellus hair of diameter > 30 μm per unit area. Methods had to include performance of macrophotography or phototrichogram techniques on hair clipped to approximately 1 mm, at the same position on the scalp, and typically marked by tattoo, for repeated measures. Only RCTs were eligible to maximize the quality of evidence (29).

Studies were excluded if results were not separated for women or men, outcomes of interest were not measured or not reported, study size had fewer than 20 subjects or if a treatment arm had less than 10 subjects. Also excluded were those studies containing diagnosis of scarring alopecia, alopecia areata, and non-specific diagnoses that could not be interpreted explicitly as AGA—such as self-reported hair thinning, thinning hair, or patchy hair loss. Studies were also excluded when hair was counted manually or from biopsies or phototrichoscopies of unclipped hair, or when results were measures of total hair count that included vellus hair, percentage changes, or if there was no standard error that was either reported or that could be derived at 24 weeks, or when the design had a treatment crossover before 24 weeks, or when the intervention was experimental or not commercially available. In studies in which interim or partial results were previously published, the most recent article was selected. Studies published in languages other than English or German were excluded.

### 2.2. Study screening and data extraction

Two research teams independently screened titles, reviewed abstracts and full-text articles, extracted data, and assessed the risk of bias of included studies. The adjudicating reviewer panel resolved discrepancies, and external experts were consulted when necessary. Missing data were obtained by contacting authors and sponsors, and from additional data reported on clinicaltrials.gov. Extracted information included: Author, date, treatment, dose, country of study, sex, race, Fitzpatrick skin type, hair loss pattern and severity, scalp site studied, age, number of subjects randomized into each treatment group, number of subjects evaluated for outcomes at, or subsequent to 12 and 24 weeks, TH count at baseline (BL), change in TH density from BL at, or near 12 weeks, 24 weeks and study mid or endpoints, variances of changes from baseline as standard deviations (SD) and *p-*values. Imputed data and missing SD for changes from baseline were estimated using Cochrane’s methodology (30). All eligible studies were included in the NMA and the continuous outcomes analysis (COA). The number of subjects and TH changes were extracted for continuous efficacy evaluation at each available study timepoint. All TH counts and densities were standardized to a unit area of 1 cm^2^ at 12 and 24 weeks by interpolation between timepoints. Studies and cohorts were separated by sex into groups of women or men only for analysis.

### 2.3. Efficacy measures and synthesis

Each study cohort was assessed for efficacy at 12 and 24 weeks with 95% confidence intervals (CI) and *p-*values as the mean difference (MD) between treatment vs. Placebo measured in TH/cm^2^. Placebo, sham, or vehicle comparators for each study cohort are referred to as Placebo in the data synthesis and results. The primary outcome measure of the Bayesian NMA was MD of treatment vs. Placebo, measured in TH/cm^2^ with 95% credible intervals (CrI).

### 2.4. Statistical analysis methods

#### 2.4.1. Network meta-analyses

NMA outcomes were reported in forest plots, league tables, composite heat-mapped outcomes picto-tables, and netgraphs.

For each efficacy outcome, an NMA was conducted using Bayesian random-effects models. Studies with more than one cohort of the same treatment, with the same effective dose that was compared to the same Placebo cohort, were combined using Cochrane methods (30). Bayesian NMAs were seeded with uniform priors and simulated 250,000 times in a Markov Chain Monte Carlo (MCMC) model (30–32). For each outcome, the network of treatments was depicted through a netgraph as an integrated diagram with nodes representing each treatment and edges where treatments were compared to Placebo or another treatment in a head-to-head trial (30, 33).

League tables for women and men at 12 and 24 weeks were produced to present the relative efficacy for every possible direct and indirect pairwise combination. The point estimate for league tables was the MD, with 95% CrIs, CIs and significance (32).

Surface Under the Cumulative Rank Area (SUCRA) scores for each treatment to determine the relative confidence were estimated and ranked with the highest value corresponding to the most effective treatment (34).

#### 2.4.2. Quality of evidence within studies and across networks

Each eligible study was evaluated for risk of bias using the Cochrane Collaboration risk of bias assessment tool using qualitative judgment for five domains: randomization, deviations from intended intervention, missing outcomes data, measurement of the outcome, and selective reporting (30, 33). A separate overall risk of bias assessment was conducted (30). Each domain was judged as low, some concern, moderate, or high risk of bias (30).

For each NMA outcome, the quality of evidence across a network was assessed using the Confidence in Network Meta-Analysis (CiNeMA) framework based on the Grading of Recommendations Assessment, Development, and Evaluation (GRADE) approach (29, 33). Under the CiNeMA framework, qualitative and quantitative judgments were made across six domains: Within-study Cochrane risk of bias, reporting bias, indirectness, imprecision, heterogeneity, and incoherence (33, 35). In the CiNeMA framework, a confidence rating is an overall qualitative assessment that summarizes judgment that assesses the quality of evidence; Confidence ratings are high, moderate, low, or very low (33).

#### 2.4.3. Continuous outcomes analysis (COA)

Weekly data points were imputed for each study cohort via linear extrapolation between reported timepoints. Weekly outcomes for each treatment were determined using a weighted MD. Results were plotted on a standard outcome in TH/cm^2^ vs. time graph for each treatment’s COA. Error analysis was not performed for the COA due to sparse availability of change error data on the interim data points.

#### 2.4.4. Frequentist analysis

Frequentist NMA and MA analyses were performed for comparison purposes through a random effects model. Frequentist results were provided as MD with 95% CI, *p-*values, and z-scores (30). A statistical significance of *p* < 0.05 was used.

#### 2.4.5. Composite outcomes evaluation

A composite range of outcomes in women and men were evaluated at 12 and 24 weeks. Continuous outcomes were determined to at least 24 weeks. Domains of outcomes were evaluated as an estimate of treatment efficacy, SUCRA score, regrowth rate, regrowth profile, and quality of evidence.

#### 2.4.6. Statistical analysis software

R studio software version 4.1.2 (2021-11-01) was used for all analyses using a significance level of α = 0.05 (36). R package “meta” was used for meta-analysis, “netmeta” and “BUGSnet” were used for NMA, “ggplot2” was modified for Kilim plots, Bayesian forest plots, netgraphs, and CiNeMA plots (32, 33, 35–38). Microsoft Excel was used for continuous outcomes imputation and analysis.

### 2.5. Safety

Safety data as reported in the included studies were summarized.

### 2.6. Study registration

Study registration on PROSPERO: CRD42021268040. Ethics approval was not required since there were no direct human treatments or observations that used medical records. The authors declare adherence to PRISMA reporting guidelines for NMAs.

## 3. Results

### 3.1. Eligible studies

**FIGURE 1:**
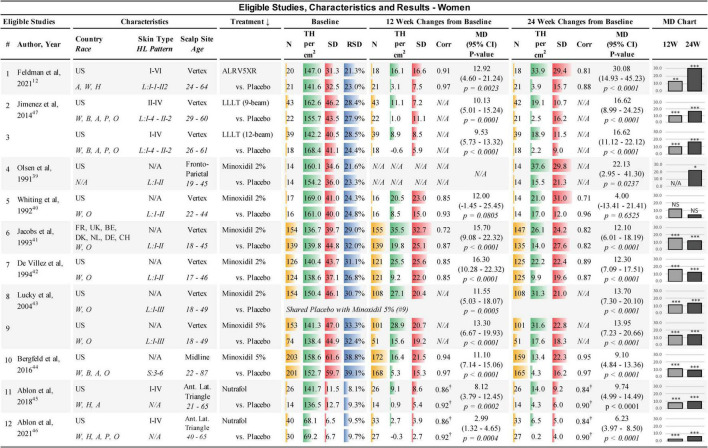
Eligible studies, characteristics, and results — Women. ↓, primary sort by treatment (secondary by year of study); Country: US, United States of America; FR, France; UK, United Kingdom; BE, Belgium; DK, Denmark; NL, Netherlands; DE, Germany; CH, Switzerland. Race: W, White; H, Hawaiian; A, Asian; B, African; P, Pacific; O, other; Skin Type, Fitzpatrick I-VI; HL, hair loss pattern (Ludwig or Savin scale); Scalp Site is observed position in study; Age, study-cohort age range; N, sample population in each arm; TH, terminal hair (mean count per cm^2^); SD, standard deviation; RSD, relative SD (SD/TH); Corr, correlation factor; †, imputed corr; MD, mean difference between treatment and Placebo; 95% CI = 95% confidence interval; 12W and 24W, 12 and 24 weeks of treatment; bar chart colors for N, TH, SD, RSD, and MD chart are for illustrative purposes; *N/A* = not available, NS = not statistically significant; *, ^**^, and ^***^ are frequentist statistically significant *P*-values < 0.05, < 0.01, and < 0.001, respectively.

**FIGURE 2:**
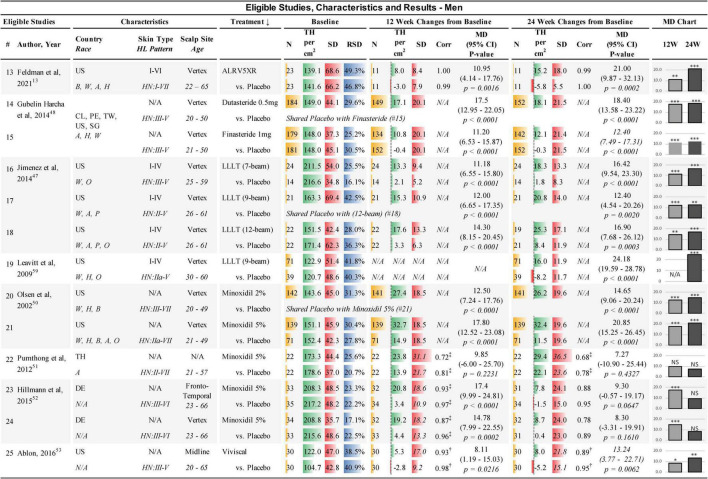
Eligible studies, characteristics, and results — Men. ↓, primary sort by treatment (secondary by year of study); Country: US, United States of America; CL, Chile; PE, Peru; TW, Taiwan; SG, Singapore; DE, Germany; Race: W, White; H, Hawaiian; A, Asian; B, African; P, Pacific; O, other; Skin Type, Fitzpatrick I-VI; HL, hair loss pattern (Hamilton-Norwood scale); Scalp Site is observed position in study; Age, study-cohort age range; N, sample population in each arm; TH, terminal hair (mean count per cm^2^); SD, standard deviation; RSD, relative SD (SD/TH); Corr, correlation factor; ‡, derived from 6-month endpoint Corr; †, derived from average Corr; MD, mean difference between treatment and Placebo; 95% CI = 95% confidence interval; 12W and 24W, 12 and 24 weeks of treatment; bar chart colors for N, TH, SD, ratio, and MD chart are for illustrative purposes; N/A, not available; NS, not statistically significant; *, ^**^, and ^***^ are frequentist statistically significant *P*-values < 0.05, < 0.01, and < 0.001, respectively. Details from studies 14 and 15 (48) were clarified from Clinicaltrials.gov: NCT01231607.

**FIGURE 3:**
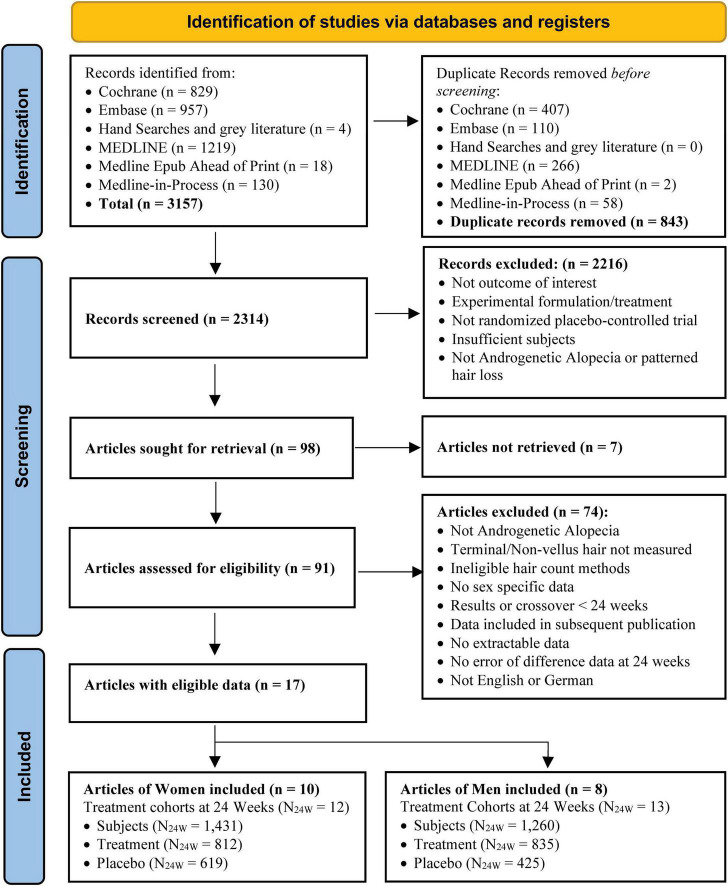
Search process of eligible studies.

The search identified 3,157 records. After removing 843 duplicates, 2,314 unique records were screened, and 98 studies were eligible for full-text review. Seven studies were not retrievable, 2 had repeated data in other studies or with different authors, and 72 did not meet the eligibility criteria, leaving a total of 17 eligible studies (12, 13, 39–53), 10 with eligible cohorts in women (12, 39–47, 53) and eight with eligible cohorts in men (13, 47–53). Data were extracted and included in the analysis from 25 treatment cohorts (12 for women, 13 for men) using eight treatments (5 for women, 7 for men), involving a total of 3,056, 2,575, and 2,691 subjects at BL, 12 and 24 weeks, of whom 1,662, 1,429 and 1,431 were women and 1,394, 1,146 and 1,260 were men, respectively. At 12 weeks, women had 832 treatment and 597 Placebo subjects, and men had 736 and 410, respectively. At 24 weeks, women had 812 treatment and 619 Placebo subjects, and men had 835 and 425, respectively (see Figures 1–3 and Supplementary Appendix 2 for more details of studies and treatments searched, screened, and excluded with reasons).

### 3.2. Treatments in eligible studies

The treatments included in this study were: A) The oral drugs Dutasteride 0.5 mg/day and Finasteride 1 mg/day in men; (B) The topical drugs Minoxidil 2% twice per day and Minoxidil 5% once per day; (C) The LLLT comb devices of the same effective dose; and, (D) The natural treatments ALRV5XR in both sexes, Nutrafol in women, and Viviscal in men (see Figures 1, 2).

### 3.3. Extracted data

Data extracted from the eligible studies included study and subject characteristics, subject demographics, sample sizes, and TH/cm^2^ at BL, with changes in TH/cm^2^ and SD at 12 and 24 weeks for each treatment cohort and Placebo for NMA purposes (see Figures 1, 2). Additional timepoint data of density changes were extracted for up to 48 weeks for continuous analysis (see Supplementary Appendix 1).

### 3.4. Imputed data

Missing SDs at 12 weeks in one study were imputed using the Cochrane correlation factors determined from BL and 24 week data within the study (51) (see Figures 1, 2). Three studies that did not have sufficient data to determine SDs from *p-*values or internal correlation factors, were estimated by taking the average of the correlation factors from the available data in the eligible studies (45, 46, 53). These average correlation factors at 12 and 24 weeks for women were (treatments: 0.855 and 0.837; Placebo 0.918 and 0.901) and for men were (treatments: 0.932 and 0.887; Placebo 0.977 and 0.947), respectively, and were used to estimate the SDs of change from BL in three studies (45, 46, 53) (see Figure 1, 2).

### 3.5. Study characteristics

Studies were conducted internationally. Eleven of the 12 studies in women and eight of the 13 studies in men were exclusively in the USA. A broad range of races were reported in the studies; however, most subjects were Caucasians. Fitzpatrick skin types were not available for most of the studies. All scalp study sites were at the vertex of the scalp, except for four study cohorts in women that were located at the frontoparietal, anterior lateral triangle, and midline. In men, two study cohorts were at the frontotemporal and midline. BL TH densities in women were 68.1–169.0 TH/cm^2^. BL SDs in women ranged from 6.5–61.6 TH/cm^2^ and the BL relative SDs (RSDs) of SD/TH ranged from 8.1 to 39.1%. BL TH densities in men were 104.7–217.2 TH/cm^2^. BL SDs in men ranged from 34.8 to 69.4 TH/cm^2^ and RSDs ranged from 16.1 to 49.3% (see Figures 1, 2). Dutasteride 0.5 mg, Finasteride 1 mg, Minoxidil 2% and Viviscal were in subjects < 51 years. All other treatments also included subjects > 60 years. Races, ranges of Fitzpatrick skin types, age ranges, and hair loss patterns of participants in these studies are described in Figures 1, 2.

All studies were found to be statistically different from Placebo in women except for one study in Minoxidil 2% at 12 and 24 weeks (40). In men, one cohort at 12 weeks and three cohorts at 24 weeks of Minoxidil 5%, were not statistically significantly different from Placebo (51, 52) (see Figures 1, 2).

### 3.6. Bayesian network meta-analyses of treatments vs. Placebo

#### 3.6.1. Women

**FIGURE 4:**
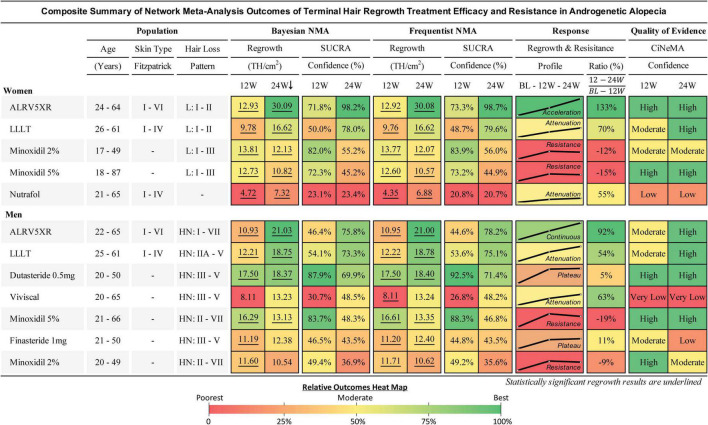
Composite summary of terminal hair regrowth and resistance from network meta-analysis outcomes for treatments in androgenetic alopecia. This outcomes table provides an overview of the studied populations, Bayesian and frequentist network meta-analyses (NMAs), response profiles showing regrowth and resistance, and quality of evidence for each treatment. Women and men are ranked in descending order of efficacy at 24 weeks of the Bayesian NMA after 250,000 Markov Chain Monte Carlo simulations. Regrowth measures efficacy as mean difference (MD) in TH/cm^2^ between each treatment and Placebo using direct and indirect comparisons at 12 and 24 weeks. Hair loss pattern: L = Ludwig (women), HN = Hamilton-Norwood (men). BL, 12W, and 24W refer to baseline, 12 weeks, and 24 weeks duration of treatment. SUCRA: surface under the cumulative ranking area measures relative confidence of outcomes of the compared treatments. Response Profile is a line graph connecting the treatment changes from baseline (BL) to MD at 12 and 24W. Y-axes (MD) are scaled 0–31 for women and 0–22 for men. Response Ratio (%) is change in regrowth of 12–24W compared to BL–12W. Quality of evidence is CiNeMA results using Cochrane risk of bias, GRADE scores, and study data. Heat maps are shared between each sex’s numerical result block and manually assigned to categorical results.

**FIGURE 5:**
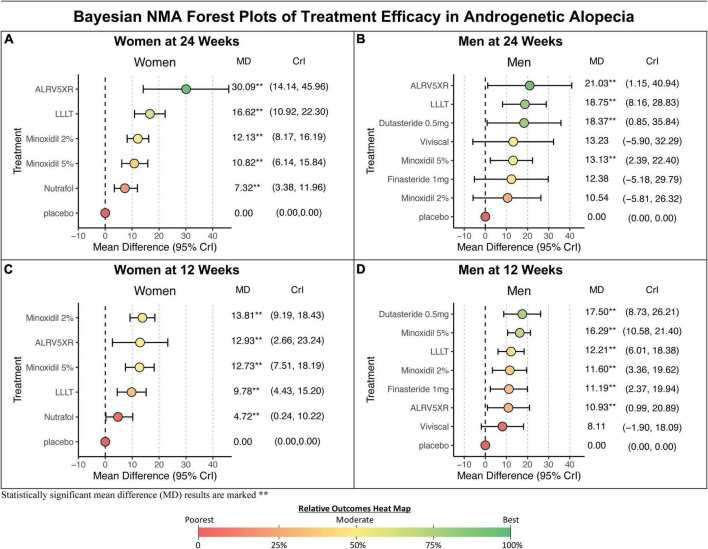
Bayesian NMA forest plots. Results of Bayesian network meta-analyses (NMA) forest plots show mean differences (MD) between treatment vs. Placebo for changes in terminal hair per cm^2^ from baseline. **(A,C)** Results for women. **(B,D)** Results for men. Results are ranked in descending order of MD with 95% credible intervals (CrI). Bayesian statistical significance is marked, **. The colors of treatment MD’s correspond with the heat map colors of Bayesian NMA results in Figure 4.

**FIGURE 6:**
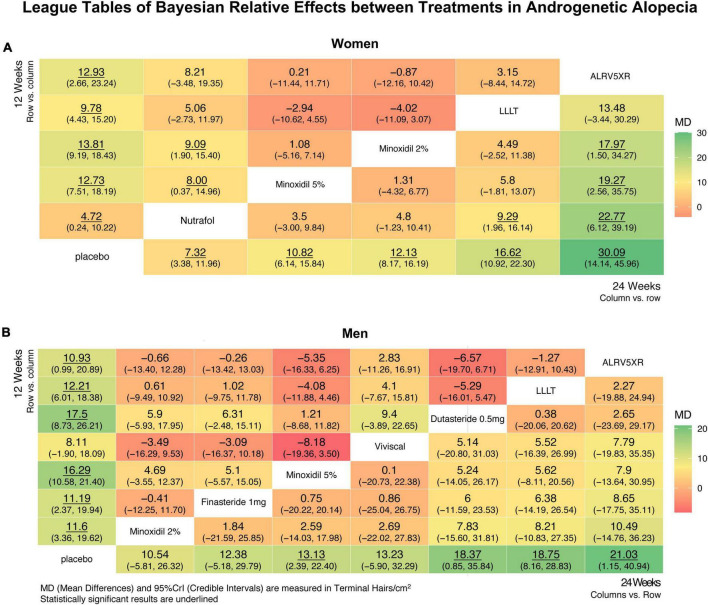
League tables showing relative effects between treatments after Bayesian network meta-analysis. These results show mean differences (MD) in regrowth of terminal hairs per cm^2^ (95% Credible Interval) between each treatment at 12 and 24 weeks in women **(A)** and men **(B)**, with significant differences underlined. League tables are arranged with 24 week results in the lower right diagonal and 12 week results in the top-left diagonal. Twenty four week results are treatment column vs. row, and are ranked in ascending order vs. Placebo from the bottom left row to bottom right. Twelve week results are treatment row vs. column. The largest positive differences are in dark green, and the largest negative differences are in dark red, and intermediate differences are shaded yellow accordingly.

In women at 24 weeks, the Bayesian NMA, using direct and indirect treatment comparisons, found MD in TH regrowth for each treatment group vs. Placebo in TH/cm^2^ (Bayesian significance = ^**^) ranked as follows: ALRV5XR: 30.09^**^, LLLT: 16.62^**^, Minoxidil 2%: 12.13^**^, Minoxidil 5%: 10.82^**^, and Nutrafol: 7.32^**^. At 12 weeks, the ranked women’s results were: Minoxidil 2%: 13.81^**^, ALRV5XR: 12.93^**^, Minoxidil 5%: 12.73^**^, LLLT: 9.78^**^, and Nutrafol 4.72^**^. MDs in the frequentist NMAs were similar to the Bayesian and at 24 weeks, all frequentist NMA results were significant (see Figures 4–6 and Supplementary Appendix 1).

#### 3.6.2. Men

In men at 24 weeks, the Bayesian NMA, using direct and indirect treatment comparisons, found the following ranked MD of each treatment group vs. Placebo in TH/cm^2^ (Bayesian significance = ^**^); ALRV5XR: 21.03^**^, LLLT: 18.75^**^, Dutasteride 0.5 mg: 18.37^**^, Viviscal: 13.23, Minoxidil 5%: 13.13^**^, Finasteride 1 mg: 12.38, and Minoxidil 2%: 10.54. At 12 weeks, the men’s results ranked; Dutasteride 0.5 mg: 17.50^**^, Minoxidil 5%: 16.29^**^, LLLT: 12.21^**^, Minoxidil 2%: 11.60^**^, Finasteride 1 mg: 11.19^**^, ALRV5XR: 10.93^**^, and Viviscal: 8.11. Bayesian statistical significance at 12 weeks was lost at 24 weeks for Finasteride 1 mg and Minoxidil 2%, and Viviscal was not significant at either 12 or 24 weeks. MDs in the frequentist NMAs were similar to the Bayesian and at 12 weeks all the treatments were significant, however, at 24 weeks, Viviscal was not significant and Minoxidil 2% was borderline significant (*p* = 0.0491) (see Figures 4–6 and Supplementary Appendix 1).

### 3.7. Regrowth from 12 to 24 weeks vs. BL to 12 weeks

Comparing regrowth from 12-24 weeks vs. BL-12 weeks, ALRV5XR showed an accelerating regrowth effect in women (133%) and an effectively continuous regrowth effect in men (92%). Resistance effects were observed in all other treatments after 12 weeks. There was an attenuated regrowth effect from 12-24 weeks vs. BL-12 weeks for LLLT (women: 70%, men: 54%), Nutrafol (women: 55%), and Viviscal (men: 63%). A flatlining effect was observed after 12 weeks for Dutasteride 0.5 mg (men: 5%) and Finasteride 1 mg (men: 11%). After peaking at 12 weeks, some of the regrowth was lost by 24 weeks for Minoxidil 2% (women: -12%, men: -9%) and Minoxidil 5% (women: -15%, men: -19%) (see Figures 4–6 and Supplementary Appendix 1).

### 3.8. NMA relative effects of treatment vs. treatment

In women, the Bayesian NMA relative effect results found at 24 weeks that ALRV5XR had a significantly greater MD in TH/cm^2^ (Bayesian significance = ^**^) than Nutrafol by 22.77^**^, Minoxidil 5% by 19.27^**^ and Minoxidil 2% by 17.97^**^, and LLLT was greater than Nutrafol by 9.29**. At 12 weeks, Minoxidil 2% and 5% were significantly greater than Nutrafol: 9.09^**^ and 8.00^**^ TH/cm^2^.

In men, there were no significant differences in NMA relative effects between treatments at 12 or 24 weeks. (For more details, see Figure 6 and Supplementary Appendix 1).

### 3.9. Surface under the cumulative rank area (SUCRA) scores

SUCRA scores can be found in Figure 4 and cumulative ranking curves can be seen in Supplementary Appendix 1.

### 3.10. Continuous outcomes analysis

**FIGURE 7:**
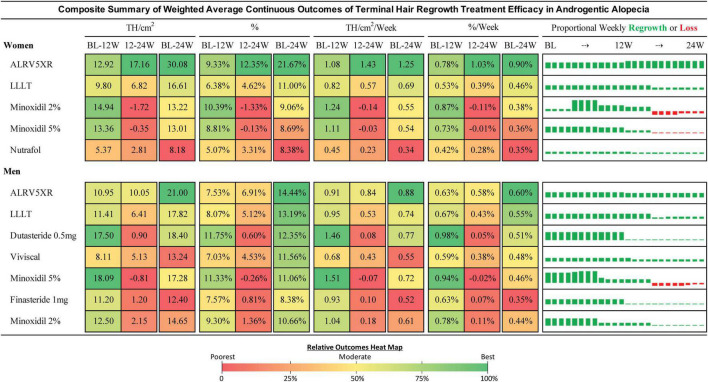
Composite summary of terminal hair regrowth from weighted average continuous outcomes analysis. This composite outcomes table provides a comprehensive overview of the treatments from weekly imputed data points for all eligible studies. Efficacy of each treatment is the difference between treatment and Placebo weighted by the N of each respective cohort. Efficacy percent is the change divided by the baseline of each cohort and weighted by the N of each cohort population. The bar charts show weekly regrowth or loss for each of the 24 weeks in TH/cm^2^. Treatments are sorted by the 24 week efficacy determined in the Bayesian NMA. Results may be different from the NMA due to analytical methods. Continuous outcomes have no measures for statistical significance. Heat map colors are green (highest result), yellow (moderate), and red (lowest). Heat maps are shared between BL-12W and 12-24W. The BL-24W column has its own heat map for the overall result. Heat maps are separate for women and men.

**FIGURE 8:**
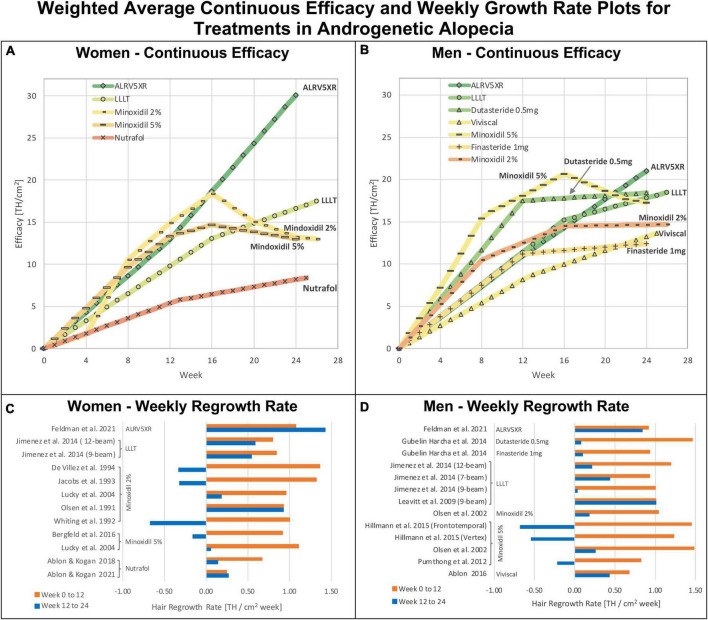
Weighted average continuous efficacy and weekly regrowth rate plots: Weighted average continuous efficacy plots are in panels **(A)** for women and **(B)** for men. Average weekly regrowth rates from 0 to 12 weeks and 12–24 weeks are in panels **(C)** for women and **(D)** for men. The continuous efficacy line graphs show the cumulative changes in efficacy for each treatment by sex. They identify two distinct regrowth response profiles common to each treatment in both sexes: Continuous: (ALRV5XR); Resistant: Dutasteride 0.5 mg, Finasteride 1 mg, LLLT, Minoxidil 2% and 5%, Nutrafol, and Viviscal. The colors of treatments in continuous efficacy response lines correspond with the 24 week heat map colors in the Bayesian network meta-analysis in Figure 4.

Continuous outcomes can be found in Figures 7, 8 and Section 4 of Supplementary Appendix 1.

### 3.11. Risk of bias and quality of evidence

The Cochrane risk of bias instrument assessed four studies (41, 45, 46, 53) with a high risk of bias, nine studies (13, 40, 42, 43, 47–49, 51, 52) with some concerns, and four studies (12, 39, 44, 50) with no overall concerns.

The summarized quality of evidence can be found in Figure 4, and the supporting Cochrane risk of bias assessments, CiNeMA results and conflict of interest data can be found in Section 3 of Supplementary Appendix 1.

### 3.12. Safety

A summary of treatment adverse events as reported in the respective trials can be found in Section 6 of Supplementary Appendix 1.

### 3.13. Frequentist results

Changes in efficacy from 12 to 24 weeks were similar between Bayesian and frequentist NMAs. Results from frequentist NMAs with SUCRA scores after 12 and 24 weeks of treatment can be found in Figure 4. Additional frequentist results can be found in Supplementary Appendix 1.

## 4. Discussion

This study is distinctive since it used comparable data from RCTs to determine TH density change as the primary marker of scalp hair phenotype. It used multiple standard analytical methods at key treatment time points, 12 and 24 weeks, and evaluated women and men separately. This approach enabled the detection of discrete insights and treatment nuances. In addition, it used rigorous methods to screen studies to ensure accurate eligibility. Opinions from independent experts, some of whom have led pivotal RCTs in AGA treatments since the late 1980’s, helped guide interpretation of eligible studies, methods, and data. Similar confirmatory results were obtained using a comprehensive range of accepted analytical methods, providing a valuable decision-making tool for clinical practitioners and, eventually, for patient self-evaluation of treatment options.

### 4.1. Treatment resistance effects

For the treatments that showed resistance, the first 12 weeks of treatment was the phase of the highest rates of TH regrowth. Thereafter, resistance affected each treatment at different rates with the drugs showing the most pronounced resistance. Men and women showed similar timing of resistance for the same treatments, particularly LLLT and Minoxidil. Nutrafol in women and Viviscal in men showed very similar resistance profiles. LLLT seems to have a reduced resistance effect and its mild attenuation maintained positive regrowth rate. The continuous regrowth effects observed with ALRV5XR appear to bypass or inhibit resistance factors (see Figures 4–8 and Supplementary Appendix 1). Possible combinations of ALRV5XR with other treatments may therefore be synergistic.

### 4.2. Bayesian vs. frequentist network meta-analysis

The Bayesian and frequentist NMAs were in agreement with MDs. In contrast, all the Bayesian 95% CrI’s were wider than the frequentist 95% CI’s by an average of 26.0% in women and 30.8% in men. A sensitivity analysis of the 24-week Bayesian NMA in men for the changes in significance from 12 to 24 weeks simulated 50,000, 150,000, 300,000, and 500,000 MCMC iterations and found no meaningful change to the 250,000 MCMC simulation results (see Supplementary Tables 1-1, 1-3 in Supplementary Appendix 1). Due to the sparseness of the study data, we believe that a Bayesian NMA provides more accurate, statistically relevant, and conservative results than the frequentist NMA.

### 4.3. Women vs. men

The MD regrowth profiles were similar between sexes for the same treatment type. The analysis found that the widths of CrI’s were narrower in women than in men by 55.9% (13.90 vs. 31.54 TH/cm^2^). Women had slightly lower efficacy than men for LLLT (11.4%) and Minoxidil 5% (17.6%), while Minoxidil 2% was slightly better in women than men (15.1%), however, ALRV5XR showed a much larger efficacy in women than men by 43.6% (30.11 vs. 20.97 TH/cm^2^). Analysis of the differences in the same treatment between women and men found no statistical significance (see Supplementary Tables 1-2, 1-3 in Supplementary Appendix 1).

### 4.4. Data anomalies, outliers and interpretation

Most studies reported mean BL TH densities consistent with the evaluated scalp sites for the eligible hair loss patterns, except for one study in women that was > 3 SDs less than the mean in both the treatment and Placebo groups (46). Two studies in women had a BL RSD < 10% compared to 21.3–39.1% in the other women’s studies (45, 46). Another study in men reported and referenced three different target area sizes in its methods for the hair density measurements (53). A target area of 1 cm^2^ was assumed since it resulted in normal BL TH density relative to the other studies. All data was extracted as reported according to Cochrane methods without interpretations.

### 4.5. Confidence, risk of bias and conflicts of interest

The inclusion of only RCTs in this NMA assisted in increasing the overall confidence of the GRADE and CiNeMA assessments. At 24 weeks, the CiNeMA confidence evaluations for each treatment *vs.* Placebo, found no concerns for women, and in men there were no major concerns, therefore no investigations into causes of heterogeneity or small study bias were deemed necessary. Studies most impacted by the risk of bias assessment were found wanting in reporting randomization procedures and selected reporting of results. The two largest effectors of the CiNeMA analysis results were the Cochrane risk of bias, which downgraded confidence in almost all studies, while the high magnitude effect upgraded confidence in ALRV5XR, LLLT and Dutasteride treatments.

All studies, except for one (51), declared a conflict of interest relating to study funding by the owner of the investigational treatment. Authors in 11 studies (12, 13, 41, 42, 44–46, 48–50, 52) were affiliated with or employed by the study sponsor. Five studies (40–42, 48, 50) had trichometric data generated by the sponsor or investigators employed by the sponsor (see Supplementary Table 1-5 in Supplementary Appendix 1).

After considering the results of the risk of bias and CiNeMA, no study conflict was deemed to have a sufficiently differential impact on the outcome of this NMA, that would require its exclusion. To further improve the confidence in this study the AMSTAR 2 (54) and ROBIS (55) appraisal tools have been used as a guide. Therefore, the confidence reported in Figure 4 can be considered a reasonable assessment.

### 4.6. Ineligible treatments of interest

Stem cells, PRP, oral Minoxidil, topical Finasteride, additional off-label drugs other than dutasteride, combination treatments, other laser, and electrostatic devices, natural treatments and cosmetics with hair loss improvement claims were of interest for this study; however, no eligible studies were found. Recently, the sub lingual use of Minoxidil in AGA with doses ranging from 0.45 to 4.05 mg has been reported, but it did not fit the inclusion criteria for this study (56). Also, a recent review reported PRP preparation and administration protocols have a broad variability, and there is no current consensus on which protocol provides the best results (57). Although no single vitamin, mineral, botanical, collagen, or protein eligible studies were found, many of these ingredients of interest are part of the natural treatments included in this study.

### 4.7. Future treatment directions addressing the biology of AGA

A need exists for improved AGA hair regrowth treatments using innovative hair follicle (HF) regenerative strategies that aim to prolong anagen, induce neogen in involuted HFs, and reverse the miniaturization of terminal HFs (58). Amongst the main identified biological targets that induce HF regeneration are the Wnt/β-catenin cascade and growth factors such fibroblast growth factor (FGF), vascular endothelial growth factor (VEGF), transforming growth factor β (TGF-β), insulin-like growth factor (IGF), endothelial cell growth factor (ECGF), and epidermal growth factor (EGF) (57, 58).

At present, multi-targeting treatments with HF regenerative effects have clinical evidence with ALRV5XR, show promise for PRP, and early work in wound induced hair neogenesis (WIHN) indicates potential as a novel treatment option (57–60). A combination of ALRV5XR and PRP may lead to an improved hair regrowth efficacy and a prolonged continuous regenerative response.

### 4.8. Hard evidence of treatments for AGA

The very small proportion of eligible studies based on the inclusion criteria (17 of the 2,314 unique records or 0.73%) measuring TH indicates there is very limited hard published evidence of TH regrowth. Furthermore, the eight eligible treatments found for this study represent a very small number of the commercially available treatment options claiming the prevention of hair loss or promotion of hair regrowth, suggesting treatments without eligible studies might have no hard published evidence.

### 4.9. First line treatment

When considering the current first-line therapies for AGA, topical Minoxidil 2% and 5%, and oral Finasteride 1 mg, these Bayesian results appear to challenge the usefulness of these treatments. Even though additional studies are required, LLLT comb devices and the natural treatment ALRV5XR appear as scientifically suitable candidates for inclusion within the AGA first-line of treatment options, alone or in combination.

## 5. Limitations

A small number of eligible studies reporting changes in TH density were found. Hindrances posed by inconsistencies in TH definition, incomparable hair-counting methods, and lack of error reporting of changes from baseline (particularly for midpoints within longer-term studies) were the most common omissions found in the studies that could not be included. Also, the study design intended to include CONSORT compliant RCTs; nevertheless, this factor was partially represented in the risk of bias and quality of evidence analysis. The lack of treatment population response data and odds ratios in the literature limited key parameters for analysis. Although this meta-analysis used trials published in English or German, it is possible that studies published in other languages may have a more comprehensive range of treatments, populations, races, skin, and hair types.

## 6. Conclusion

For most commercially available products claiming to prevent hair loss or to regrow hair, there is only a dearth of scientific proof available in the literature of TH regrowth. This meta-analysis examines the available evidence with scientifically validated methods in the few RCTs with published TH regrowth data. Despite the long-term treatment resistance and statistically significant improvement in TH reported for most treatments, the primary clinical outcome of virtually all of them was the maintenance of existing hair, or the regrowth of a small amount of hair that may decrease AGA pattern severity, with LLLT comb being more efficacious than first-line pharmaceuticals. The exception was ALRV5XR which demonstrated better efficacy without treatment resistance effects, and, in consequence, showed strong characteristics to be among the first-line AGA treatment options for men and women, alone or in combination.

## Eligible treatment trade names

Tradenames of eligible treatments in this study are ALRV5XR (Replenology), Dutasteride 0.5 mg (Avodart, generic dutasteride), Finasteride 1 mg (Proscar, Propecia, generic finasteride), LLLT comb (HairMax), Minoxidil (Rogaine, Regaine, generic minoxidil). Nutrafol and Viviscal are trade names.

## Plain language summary

Terminal hair (TH) on the scalp is thick, long hair giving a unique visual identity to each person. TH density is the most important marker of the scalp hair phenotype, yet it is one of the least reported hair loss treatment outcomes. Androgenetic alopecia, or patterned balding, affects about half the population. It is characterized by TH diminishing into short, fine vellus-like hair, which eventually stops growing and can lead to baldness. Scalp hair phenotype restoration can only be achieved by increasing TH density. This study compares the continuous efficacy of treatments claiming to promote hair regrowth. A systematic literature search found 2,314 unique published hair regrowth studies. Results could be used from only 17 studies with five different therapies in women and seven in men to statistically compare TH regrowth over 24 weeks. These treatments included drugs, laser devices, and natural products. A network meta-analysis using Bayesian statistics simulated the real-world effectiveness of each treatment. This study found that most of the included treatments will maintain existing TH density, and some might reduce the hair-loss pattern. However, responses to most treatments had a resistance effect after 12 weeks, where drugs plateaued or lost part of the regrowth, and some were not different from Placebo. Pharmaceutical treatments showed limited regrowth efficacy, and laser comb devices appeared slightly better than pharmaceuticals. The natural treatment ALRV5XR displayed the best results without resistance and might have the potential to restore TH in women and men.

## Author contributions

PRF and CP contributed to the study concept and design. PRF obtained funding and drafted the manuscript. EU developed the search strategy, performed the article search, and prepared the article database. SG, JP, MM, MIS, MB, OJF, and CLK screened the studies and extracted the data. PRF, CP, JG-A, PG, HMM, and KMF adjudicated and reviewed the data. PRF, SG, and KMF performed statistical analysis and visualization. PG, CP, HMM, JG-A, and KMF critically revised the manuscript for important intellectual content. All authors reviewed, edited, and approved the manuscript for submission.
