# Supplementary material for: Hair regrowth treatment efficacy and resistance in androgenetic alopecia: A systematic review and continuous Bayesian network meta-analysis
Source: Front Med (Lausanne). 2023 Jan 23;9:998623. doi: 10.3389/fmed.2022.998623 (PMC9900126; doi:10.3389/fmed.2022.998623)
Supplement: Supplementary file 1 [file Data_Sheet_1.pdf]

Supplementary Materials

Appendix 1 – Supplementary Tables and Figures

Hair Regrowth Treatment Efficacy and Resistance in Androgenetic Alopecia: A Systematic Review and Continuous Bayesian Network Meta-Analysis

Peter R. Feldman[1,2*], Pietro Gentile[3], Charles Piwko[4], Hendrik M. Motswaledi[5], Samantha Gorun[6,7], Jacob Pesachov[8], Michael Markel[8], Maxwell I. Silver[9,10], Megan Brenkel[11], Oriel J. Feldman[1,12], Corey L. Kamen[8], Elizabeth Uleryk[13], Jaime Guevara-Aguirre[14,15,16,17], Klaus M. Fiebig[1]

[1] Arbor Life Labs, Toronto, ON, Canada
[2] Norwich Medical School, University of East Anglia, Norwich, United Kingdom
[3] Surgical Science Department, University of Rome Tor Vergata, Rome, Italy
[4] CHP Pharma Inc., Thornhill, ON, Canada
[5] Department of Dermatology, Sefako Makgatho Health Sciences University, South Africa
[6] Faculty of Epidemiology & Biostatistics, Western University, ON, Canada
[7] School or Mathematics and Statistics, University of Glasgow, Glasgow, United Kingdom
[8] Faculty of Medicine, Technion-Israel Institute of Technology, Haifa, Israel
[9] Faculty of Dentistry, University of Toronto, Toronto, ON, Canada
[10] Faculty of Medicine and Dentistry, University of Alberta, Edmonton, AB, Canada
[11] Faculty of Medicine, University of Ottawa, Ottawa, ON, Canada
[12] Faculty of Science, Wilfrid Laurier University, Waterloo, ON, Canada
[13] Uleryk Consulting, Mississauga, ON, Canada
[14] Universidad San Francisco de Quito (USFQ), Quito, Ecuador
[15] Faculty of Health, Medicine and Life Sciences, Maastricht University, Maastricht, Netherlands
[16] Institute of Endocrinology, Metabolism, and Reproduction (IEMYR), Quito, Ecuador
[17] College of Medicine, University of Florida, Gainesville, FL, USA

*** Correspondence:**
Corresponding Author: Peter R Feldman
petefeldman@gmail.com

Keywords: hair loss, ALRV5XR, Dutasteride, Finasteride, LLLT, Minoxidil, Nutrafol, Viviscal

Submission Date: July 20, 2022
Revised: September 12, 2022
Accepted: December 7, 2022
Published: January 23, 2023

CITATION:
Feldman PR, Gentile P, Piwko C, Motswaledi H, Gorun S, Pesachov J, Markel M, Silver MI, Brenkel M, Feldman OJ, Kamen CL, Uleryk E, Guevara-Aguirre J and Fiebig KM (2023) Hair regrowth treatment efficacy and resistance in androgenetic alopecia: A systematic review and Bayesian network meta-analysis. Front. Med. 9:998623. doi: 10.3389/fmed.2022.998623

© 2023 Feldman, Gentile, Piwko, Motswaledi, Gorun, Pesachov, Markel, Silver, Brenkel, Feldman, Kamen, Uleryk, Guevara-Aguirre and Fiebig. This is an open-access article distributed under the terms of the Creative Commons Attribution License (CC BY). The use, distribution or reproduction in other forums is permitted, provided the original author(s) and the copyright owner(s) are credited and that the original publication in this journal is cited, in accordance with accepted academic practice. No use, distribution or reproduction is permitted which does not comply with these terms.

Table of Contents

Page

Section 1: Additional Bayesian NMA Results 3

Figure S1-1: Treatment Ranking Plots (Frequentist NMA) 3
Table S1-1: Bayesian NMA Sensitivity Test of Various Simulations in Men at 24 Weeks 4

Section 2: Additional Frequentist NMA Results 5

Figure S1-2: Frequentist Network Meta-Analysis Plots of Treatment Efficacy in Androgenetic Alopecia 5
Figure S1-3: Frequentist Relative Effects between Treatments 6
Figure S1-4: Treatment Ranking Plots (Frequentist NMA) *8*
Table S1-2: Statistical Comparison of differences in efficacy between the same treatment in Women and Men 9
Table S1-3: Comparison of Bayesian and Frequentist Credible and Confidence Intervals 9

Section 3: Confidence in Network Meta-Analysis and Risk of Bias Results 10

Figure S1-5: CiNeMa Network Plots and Risk of Bias between Treatments 10
Table S1-4: CiNeMA Results 11
Figure S1-6: Cochrane Risk of Bias 12
Table S1-5: Study Conflicts of Interest 13

Section 4: Continuous Outcomes Data and Additional Results 14

Table S1-6: Extracted Continuous Outcomes Data and Weekly imputed results in terminal hair regrowth in TH/ cm2 of AGA treatments in women by week 15
Table S1-7: Imputed results in terminal hair regrowth of AGA treatments in women by week in TH/ cm2 15
Table S1-8: Imputed results in terminal hair regrowth of AGA treatments in women by week in Percent (%) 16
Figure S1-6 Continuous Outcomes of regrowth of AGA treatments in women by week 16
Figure S1-7: Continuous Outcomes in Women for Treatments with Multiple Studies 17
Table S1-9: Extracted Continuous Outcomes Data and Weekly imputed results in terminal hair regrowth per cm2 of AGA treatments in men by week 18
Table S1-10: Imputed results in terminal hair regrowth of AGA treatments in men by week per cm^2 19
Figure S1-8: Continuous Outcomes of regrowth of AGA treatments in men by week 20
Table S1-11: Imputed results in terminal hair regrowth of AGA treatments in men by week in Percent (%) 20
Figure S1-9: Continuous in Men for Treatments with Multiple Studies 21

Section 5: Meta-Analysis Results 22

Figure S1-10: Meta-Analysis Forest Plot for Women after 24 weeks of treatment 22
Figure S1-11: Meta-Analysis Forest Plot for Women after 12 weeks of treatment 23
Figure S1-12: Meta-Analysis Forest Plot for Men after 24 weeks of treatment 24
Figure S1.-13: Meta-Analysis Forest Plot for Men after 12 weeks of treatment 25
Figure S1-14: Funnel Plots 26

Section 6: Safety Results 28

Table S1-12: Summary of Adverse Events (AEs) from Eligible Studies 28

Front. Med. 9:998623. doi: 10.3389/fmed.2022.998623

Section 1: Additional Bayesian NMA Results

Figure S1-1: Treatment Ranking Plots (Bayesian NMA)

Treatment Ranking Plots (Bayesian NMA)

Women

(A) **24 Weeks**

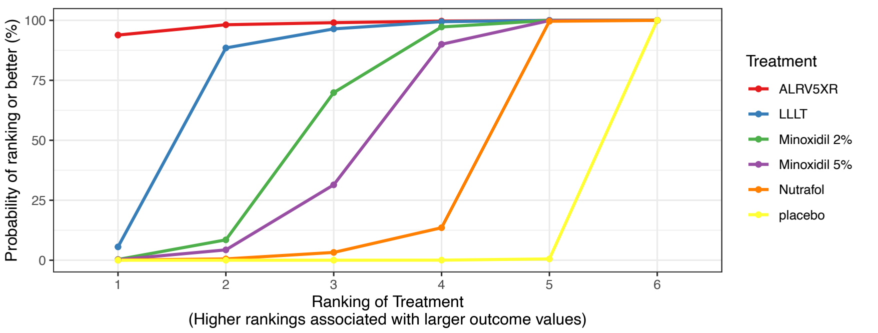

Men

(B) **24 Weeks**

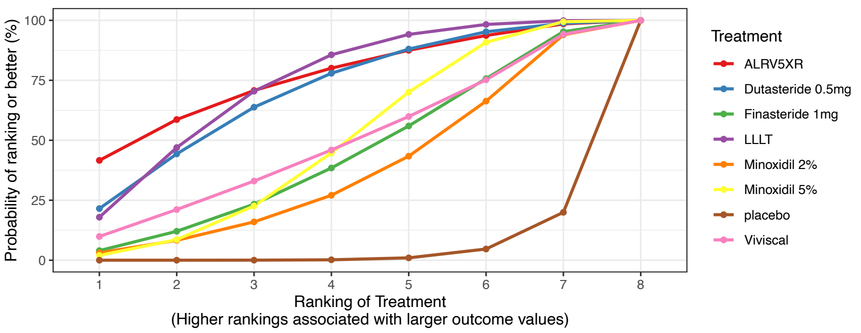

(C) **12 Weeks**

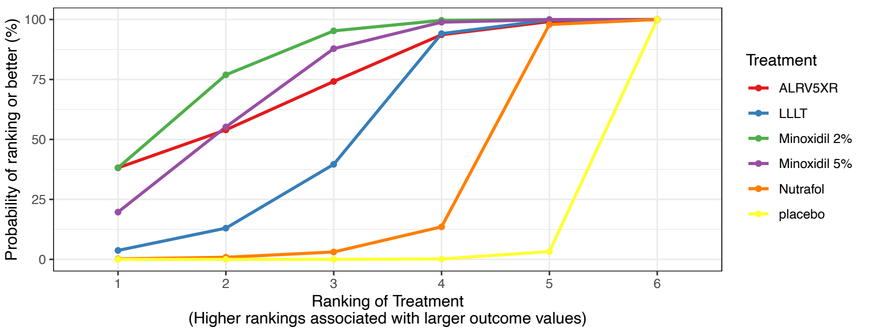

(D) **12 Weeks**

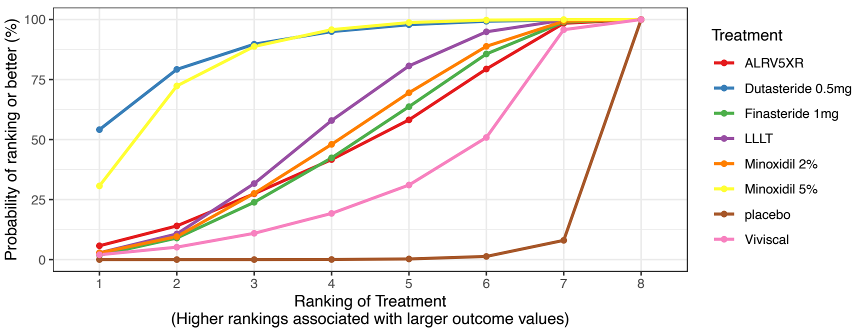

Table S1-1. Bayesian NMA Sensitivity Test of Various Simulations in Men at 24 Weeks
Sensitivity tests of the Men's Bayesian NMA evaluated the effect of simulations on MD and CrIs to look for stability or other effects that might have caused Finasteride 1mg and Minoxidil 2% to lose significance. The results showed very low variability across a wide range of simulations. Treatments with a lower CrI less than that of placebo, were found not significantly different from placebo for the Bayesian NMA.

No. Simulations	Treatment	Comparator	MD	Lower CrI	Upper CrI	CrI Width
**50k**	ALRV5XR	placebo	20.99	1.13	40.85	39.72
**150k**	ALRV5XR	placebo	21.03	1.25	40.87	39.62
**250k**	ALRV5XR	placebo	21.03	1.15	40.94	39.79
**300k**	ALRV5XR	placebo	21.02	1.13	40.94	39.81
**500k**	ALRV5XR	placebo	21.00	1.14	40.90	39.76
**50k**	Dutasteride 0.5mg	placebo	18.40	0.89	35.83	34.94
**150k**	Dutasteride 0.5mg	placebo	18.37	0.86	35.85	34.99
**250k**	Dutasteride 0.5mg	placebo	18.37	0.85	35.84	34.99
**300k**	Dutasteride 0.5mg	placebo	18.37	0.81	35.88	35.07
**500k**	Dutasteride 0.5mg	placebo	18.38	0.83	35.86	35.04
**50k**	Finasteride 1mg	placebo	12.40	-5.35	29.99	35.34
**150k**	Finasteride 1mg	placebo	12.40	-5.14	29.87	35.00
**250k**	Finasteride 1mg	placebo	12.38	-5.18	29.79	34.98
**300k**	Finasteride 1mg	placebo	12.37	-5.25	29.83	35.08
**500k**	Finasteride 1mg	placebo	12.38	-5.19	29.86	35.05
**50k**	LLLT	placebo	18.78	8.14	28.83	20.70
**150k**	LLLT	placebo	18.75	8.19	28.80	20.62
**250k**	LLLT	placebo	18.75	8.16	28.83	20.67
**300k**	LLLT	placebo	18.75	8.15	28.87	20.72
**500k**	LLLT	placebo	18.76	8.18	28.87	20.69
**50k**	Minoxidil 2%	placebo	10.54	-5.77	26.38	32.15
**150k**	Minoxidil 2%	placebo	10.55	-5.81	26.29	32.10
**250K**	Minoxidil 2%	placebo	10.54	-5.81	26.32	32.12
**300k**	Minoxidil 2%	placebo	10.54	-5.82	26.35	32.17
**500k**	Minoxidil 2%	placebo	10.54	-5.81	26.31	32.12
**50k**	Minoxidil 5%	placebo	13.12	2.40	22.43	20.03
**150k**	Minoxidil 5%	placebo	13.14	2.40	22.42	20.02
**250k**	Minoxidil 5%	placebo	13.13	2.39	22.40	20.01
**300k**	Minoxidil 5%	placebo	13.13	2.38	22.39	20.01
**500k**	Minoxidil 5%	placebo	13.13	2.40	22.39	19.99
**50k**	placebo	placebo	0.00	0.00	0.00	0.00
**150k**	placebo	placebo	0.00	0.00	0.00	0.00
**250k**	placebo	placebo	0.00	0.00	0.00	0.00
**300k**	placebo	placebo	0.00	0.00	0.00	0.00
**500k**	placebo	placebo	0.00	0.00	0.00	0.00
**50k**	Viviscal	placebo	13.24	-5.87	32.28	38.15
**150k**	Viviscal	placebo	13.22	-5.84	32.27	38.11
**250k**	Viviscal	placebo	13.23	-5.90	32.29	38.19
**300k**	Viviscal	placebo	13.24	-5.91	32.32	38.24
**500k**	Viviscal	placebo	13.23	-5.87	32.31	38.18

Front. Med. 9:998623. doi: 10.3389/fmed.2022.998623

Section 2: Additional Frequentist NMA Results

Figure S1-2. Frequentist Network Meta-Analysis Plots of Treatment Efficacy in Androgenetic Alopecia. Efficacy is measured as Mean Differences (MD) of changes from baseline of direct and indirect treatment comparisons vs. placebo in TH/cm^2. Plots for women are 24 weeks (A) and 12 weeks (C) and men are 24 weeks (B) and 12 weeks (D). Results are ranked in descending order of MD with 95% confidence intervals (CI). Statistical significance is $p < 0.0500$. The colors of treatment MD's correspond with the heat map colors of frequentist NMA results in Table 2.

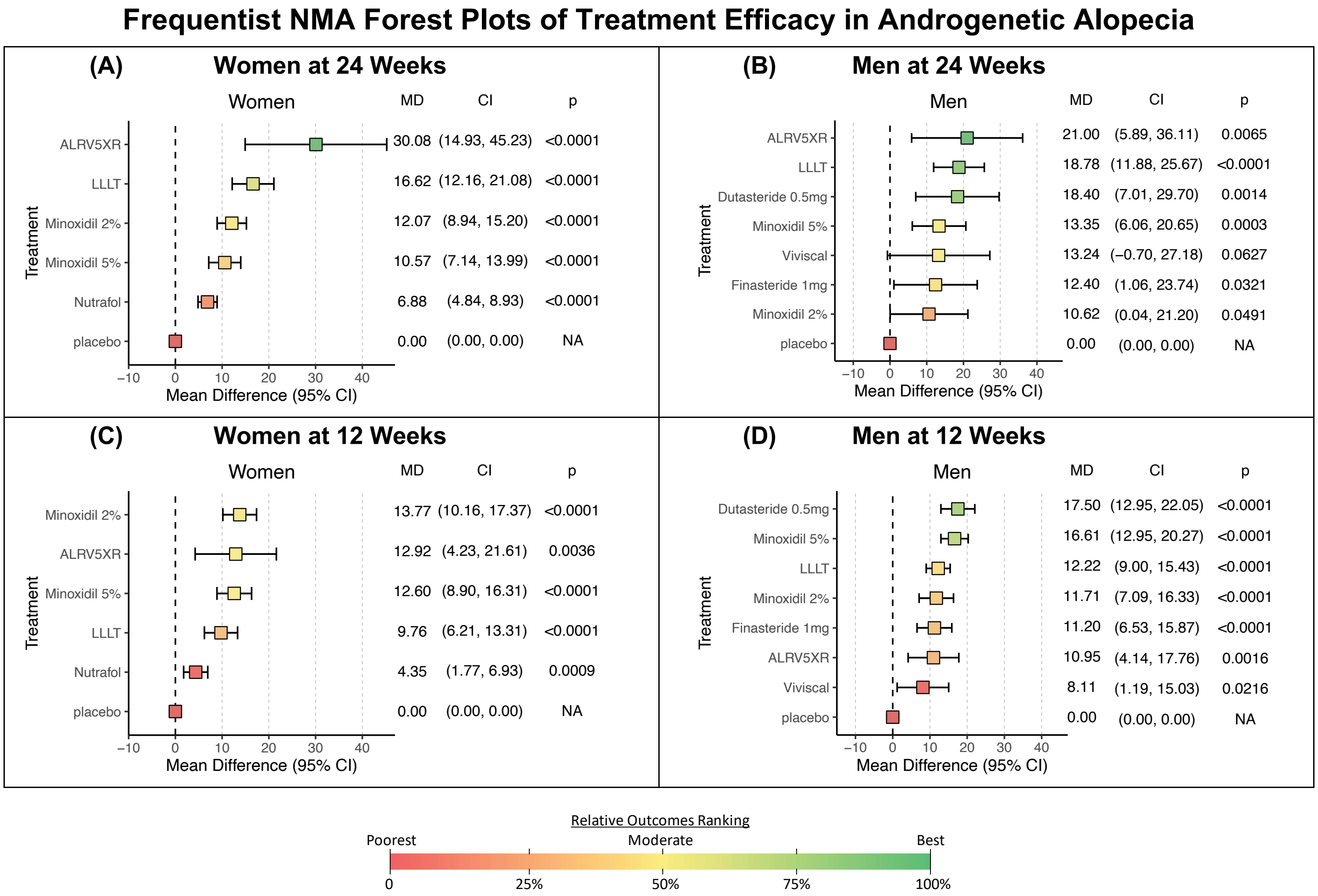

Front. Med. 9:998623. doi: 10.3389/fmed.2022.998623

Figure S1-3. Frequentist Relative Effects between Treatments.

Frequentist League Tables Showing Relative Effects between Treatments after Network Meta-Analysis of changes in terminal hair per cm2 in women at 24 weeks (A) and 12 weeks (B) and men 24 weeks (C) and 12 weeks (D) with AGA. These results show mean differences (MD) in regrowth of terminal hairs per cm^2 (95% Confidence Intervals) between each treatment at 12 and 24 weeks with significant differences underlined. League tables are ranked based on 24 week results with highest efficacy treatment from left – right and top to bottom. Differences between the treatments are rows vs. columns. At 12 and 24 weeks, all treatments were significantly greater than placebo with the exception of Viviscal at 24 weeks in men, and Minoxidil 2% in men at 24 weeks was borderline significant. At 24 weeks, In women at 24 weeks, ALRV5XR was significantly different from Minoxidil 2% and 5%, and Nutrafol. Notably, at 12 weeks in men, Dutasteride was significantly different to Finasteride and Minoxidil 5% was significantly different to Minoxidil 2%. There was no drug vs. drug significance after 12 weeks.

Frequentist Relative Effects between Treatments

Women

(A) 24 Weeks

ALRV5XR	LLLT	Minoxidil 2%	Minoxidil 5%	Nutrafol	placebo
ALRV5XR	**13.46**<br>(-2.33, 29.25)<br>z = 1.67<br>p = 0.0948	**18.01**<br>(2.54, 33.48)<br>z = 2.28<br>p = 0.0225	**19.51**<br>(3.99, 35.04)<br>z = 2.46<br>p = 0.0138	**23.20**<br>(7.91, 38.48)<br>z = 2.97<br>p = 0.0029	**30.08**<br>(14.93, 45.23)<br>z = 3.89<br>p < 0.0001
	LLLT	**4.55**<br>(-0.90, 10.00)<br>z = 1.64<br>p = 0.1016	**6.05**<br>(0.43, 11.68)<br>z = 2.11<br>p = 0.0349	**9.74**<br>(4.83, 14.64)<br>z = 3.89<br>p = 0.0001	**16.62**<br>(12.16, 21.08)<br>z = 7.30<br>p < 0.0001
		Minoxidil 2%	**1.51**<br>(-2.61, 5.62)<br>z = 0.72<br>p = 0.4735	**5.19**<br>(1.45, 8.92)<br>z = 2.72<br>p = 0.0065	**12.07**<br>(8.94, 15.20)<br>z = 7.57<br>p < 0.0001
			Minoxidil 5%	**3.68**<br>(-0.31, 7.67)<br>z = 1.81<br>p = 0.0708	**10.57**<br>(7.14, 13.99)<br>z = 6.04<br>p < 0.0001
				Nutrafol	**6.88**<br>(4.84, 8.93)<br>z = 6.59<br>p < 0.0001

Legend:
MD: Mean Difference in TH/cm^2
95% CI: 95% Confidence Interval
P-Value: Significance <0.05 (when MD underlined)
Z-Score: Significance >1.96 (when MD underlined)

(B) 12 Weeks

ALRV5XR	LLLT	Minoxidil 2%	Minoxidil 5%	Nutrafol	placebo
ALRV5XR	**3.16**<br>(-6.23, 12.55)<br>z = 0.66<br>p = 0.5092	**-0.85**<br>(-10.25, 8.56)<br>z = -0.18<br>p = 0.8601	**0.32**<br>(-9.13, 9.76)<br>z = 0.07<br>p = 0.9476	**8.57**<br>(-0.40, 17.63)<br>z = 1.85<br>p = 0.0639	**12.92**<br>(4.23, 21.61)<br>z = 2.91<br>p = 0.0036
	LLLT	**-4.01**<br>(-9.07, 1.05)<br>z = -1.55<br>p = 0.1207	**-2.84**<br>(-7.98, 2.29)<br>z = -1.09<br>p = 0.2771	**5.41**<br>(1.02, 9.80)<br>z = 2.41<br>p = 0.0158	**9.76**<br>(6.21, 13.31)<br>z = 5.38<br>p < 0.0001
		Minoxidil 2%	**1.16**<br>(-3.29, 5.62)<br>z = 0.51<br>p = 0.6089	**9.41**<br>(4.98, 13.85)<br>z = 4.16<br>p < 0.0001	**13.77**<br>(10.16, 17.37)<br>z = 7.48<br>p < 0.0001
			Minoxidil 5%	**8.25**<br>(3.74, 12.76)<br>z = 3.59<br>p = 0.0003	**12.60**<br>(8.90, 16.31)<br>z = 6.67<br>p < 0.0001
				Nutrafol	**4.35**<br>(1.77, 6.93)<br>z = 3.31<br>p = 0.0009

Figure S1-3. Frequentist Relative Effects between Treatments. (Cont.)

Relative Effects between Treatments

Men

(C) **24 Weeks**

ALRV5XR	LLLT	Dutasteride 0.5mg	Minoxidil 5%	Viviscal	Finasteride 1mg	Minoxidil 2%	placebo
ALRV5XR	**2.22**<br>(-14.38, 18.83)<br>z = 0.26<br>p = 0.7930	**2.60**<br>(-16.27, 21.47)<br>p = 0.7871<br>z = 0.27	**7.65**<br>(-9.13, 24.43)<br>z = 0.89<br>p = 0.3717	**7.76**<br>(-12.80, 28.32)<br>z = 0.74<br>p = 0.4594	**8.60**<br>(-10.29, 27.49)<br>z = 0.89<br>p = 0.3723	**10.38**<br>(-8.07, 28.82)<br>z = 1.10<br>p = 0.2701	**21.00**<br>(5.89, 36.11)<br>z = 2.72<br>p = 0.0065
	LLLT	**0.38**<br>(-12.86, 13.62)<br>z = 0.06<br>p = 0.9556	**5.42**<br>(-4.61, 15.46)<br>z = 1.06<br>p = 0.2896	**5.54**<br>(-10.02, 21.09)<br>z = 0.70<br>p = 0.4854	**6.38**<br>(-6.90, 19.65)<br>z = 0.94<br>p = 0.3464	**8.15**<br>(-4.47, 20.78)<br>z = 1.27<br>p = 0.2055	**18.78**<br>(11.88, 25.67)<br>z = 5.34<br>p < 0.0001
		Dutasteride 0.5mg	**5.05**<br>(-8.41, 18.50)<br>z = 0.74<br>p = 0.4621	**5.16**<br>(-12.79, 23.11)<br>z = 0.56<br>p = 0.5731	**6.00**<br>(-5.34, 17.34)<br>z = 1.04<br>p = 0.2997	**7.78**<br>(-7.70, 23.26)<br>z = 0.98<br>p = 0.3247	**18.40**<br>(7.10, 29.70)<br>z = 3.19<br>p = 0.0014
			Minoxidil 5%	**0.11**<br>(-15.62, 15.84)<br>z = 0.01<br>p = 0.9889	**0.95**<br>(-12.53, 14.44)<br>z = 0.14<br>p = 0.8899	**2.73**<br>(-7.66, 13.12)<br>z = 0.52<br>p = 0.6063	**13.35**<br>(6.06, 20.65)<br>z = 3.59<br>p = 0.0003
				Viviscal	**0.84**<br>(-17.13, 18.81)<br>z = 0.09<br>p = 0.9270	**2.62**<br>(-14.88, 20.12)<br>z = 0.29<br>p = 0.7692	**13.24**<br>(-0.70, 27.18)<br>z = 1.86<br>p = 0.0627
					Finasteride 1mg	**1.78**<br>(-13.73, 17.29)<br>z = 0.22<br>p = 0.8221	**12.40**<br>(1.06, 23.74)<br>z = 2.14<br>p = 0.0321
						Minoxidil 2%	**10.62**<br>(0.04, 21.20)<br>z = 1.97<br>p = 0.0491

Legend:

MD: Mean Difference in TH/cm^2
95% CI: 95% Confidence Interval
P-Value: Significance <0.05 (when MD underlined)
Z-Score: Significance >1.96 (when MD underlined)

(D) **12 Weeks**

ALRV5XR	LLLT	Dutasteride 0.5mg	Minoxidil 5%	Viviscal	Finasteride 1mg	Minoxidil 2%	placebo
ALRV5XR	**-1.27**<br>(-8.80, 6.26)<br>z = -0.33<br>p = 0.7413	**-6.55**<br>(-14.74, 1.64)<br>z = -1.57<br>p = 0.1169	**-5.66**<br>(-13.39, 2.07)<br>z = -1.43<br>p = 0.1514	**2.84**<br>(-6.87, 12.55)<br>z = 0.57<br>p = 0.5665	**-0.25**<br>(-8.51, 8.01)<br>z = -0.06<br>p = 0.9527	**-0.76**<br>(-8.99, 7.47)<br>z = -0.18<br>p = 0.8562	**10.95**<br>(4.14, 17.76)<br>z = 3.15<br>p = 0.0016
	LLLT	**-5.28**<br>(-10.85, 0.28)<br>z = -1.86<br>p = 0.0629	**-4.39**<br>(-9.26, 0.48)<br>z = -1.77<br>0.0772	**4.11**<br>(-3.52, 11.74)<br>z = 1.05<br>p = 0.2914	**1.02**<br>(-4.65, 6.69)<br>z = 0.35<br>p = 0.7249	**0.51**<br>(-5.12, 6.14)<br>z = 0.18<br>p = 0.8599	**12.22**<br>(9.00, 15.43)<br>z = 7.45<br>p < 0.0001
		Dutasteride 0.5mg	**0.89**<br>(-4.94, 6.73)<br>z = 0.30<br>p = 0.7645	**9.39**<br>(1.11, 17.67)<br>z = 2.22<br>p = 0.0262	**6.30**<br>(1.60, 11.00)<br>z = 2.63<br>p = 0.0086	**5.79**<br>(-0.69, 12.27)<br>z = 1.75<br>p = 0.0801	**17.50**<br>(12.95, 22.05)<br>z = 7.55<br>p < 0.0001
			Minoxidil 5%	**8.50**<br>(0.67, 16.33)<br>z = 2.13<br>p = 0.0334	**5.41**<br>(-0.53, 11.34)<br>z = 1.79<br>p = 0.0741	**4.90**<br>(0.77, 9.02)<br>z = 2.33<br>p = 0.0200	**16.61**<br>(12.95, 20.27)<br>z = 8.90<br>p < 0.0001
				Viviscal	**-3.09**<br>(-11.44, 5.26)<br>z = -0.73<br>p = 0.4684	**-3.60**<br>(-11.92, 4.72)<br>z = -0.85<br>p = 0.3965	**8.11**<br>(1.19, 15.03)<br>z = 2.30<br>p = 0.0216
					Finasteride 1mg	**-0.51**<br>(-7.08, 6.06)<br>z = -0.15<br>p = 0.8788	**11.20**<br>(6.53, 15.87)<br>z = 4.70<br>p < 0.0001
						Minoxidil 2%	**11.71**<br>(7.09, 16.33)<br>z = 4.97<br>p < 0.0001

Figure S1-4: Treatment Ranking Plots (Frequentist NMA)

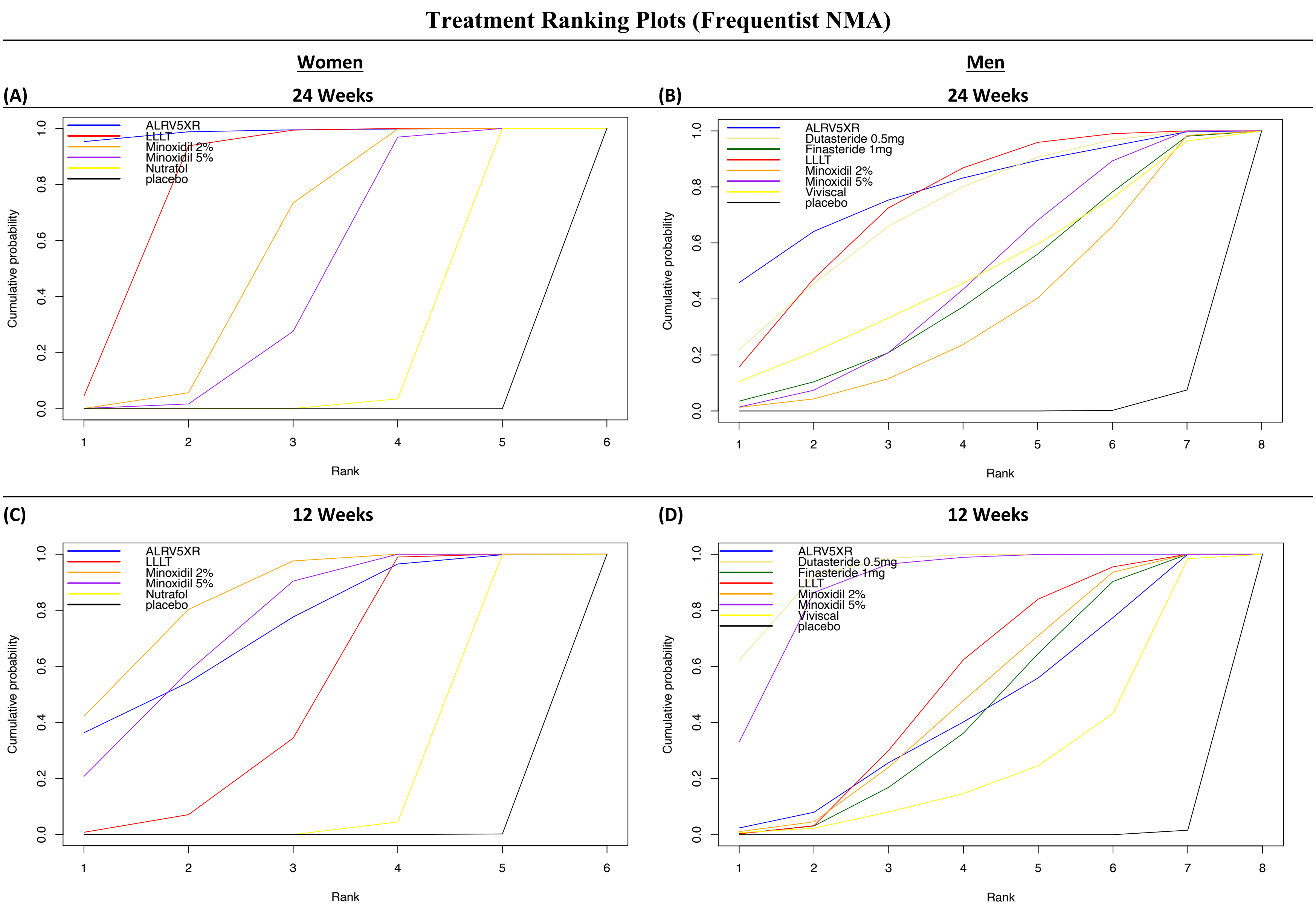

Front. Med. 9:998623. doi: 10.3389/fmed.2022.998623

Table S1-2: Statistical Comparison of differences in efficacy between the same treatment in Women and Men

Comparison	Sex Difference	p-value	95% CI
ARLV5XR - M vs. ALRV5XR - W	-9.08	0.3746	(-29.13, 10.97)
LLLT - M vs. LLLT - F	2.73	0.4619	(-4.55, 10.01)
Minoxidil 2% - M vs. Minoxidil 2% - F	-1.08	0.7898	(-8,98, 6.83)
Minoxidil 5% - M vs. Minoxidil 5% - F	3.87	0.2928	(-3.34, 11.08)

Table S1-3: Comparison of Bayesian and Frequentist MD's, Credible and Confidence Intervals

(A) Women

Sex	Treatment	Week	Analysis	MD	Difference	CI low	CI high	CI size	CI size difference	Z	P	Significant	SUCRA
Women	ALRV5XR	24	Bayesian NMA	30.09	0.03%	14.14	45.96	31.82	4.78%				98.2
Women	ALRV5XR	24	Freq NMA	30.08		14.93	45.23	30.3		3.89	<0.0001	yes	98.7
Women	LLLT	24	Bayesian NMA	16.62	0.00%	10.92	22.3	11.38	21.62%				78
Women	LLLT	24	Freq NMA	16.62		12.16	21.08	8.92		7.3	<0.0001	yes	79.6
Women	Minoxidil 2%	24	Bayesian NMA	12.13	0.49%	8.17	16.19	8.02	21.95%				55.2
Women	Minoxidil 2%	24	Freq NMA	12.07		8.94	15.2	6.26		7.57	<0.0001	yes	56
Women	Minoxidil 5%	24	Bayesian NMA	10.82	2.31%	6.14	15.84	9.7	29.38%				45.2
Women	Minoxidil 5%	24	Freq NMA	10.57		7.14	13.99	6.85		6.04	<0.0001	yes	44.9
Women	Nutrafol	24	Bayesian NMA	7.32	6.01%	3.38	11.96	8.58	52.33%				23.4
Women	Nutrafol	24	Freq NMA	6.88		4.84	8.93	4.09		6.59	<0.0001	yes	20.7

(B) Men

Sex	Treatment	Week	Analysis	MD	Difference	CI low	CI high	CI size	CI size difference	Z	P	Significant	SUCRA
Men	ALRV5XR	24	Bayesian NMA	21.03	0.14%	1.15	40.94	39.79	24.05%				75.8
Men	ALRV5XR	24	Freq NMA	21		5.89	36.11	30.22		2.74	0.0065	yes	78.2
Men	Dutasteride 0.5mg	24	Bayesian NMA	18.37	0.16%	0.85	35.84	34.99	35.15%				69.9
Men	Dutasteride 0.5mg	24	Freq NMA	18.4		7.01	29.7	22.69		3.21	0.0014	yes	71.4
Men	Finasteride 1mg	24	Bayesian NMA	12.38	0.16%	-5.18	29.79	34.97	35.14%				43.5
Men	Finasteride 1mg	24	Freq NMA	12.4		1.06	23.74	22.68		2.16	0.0321	yes	43.5
Men	LLLT	24	Bayesian NMA	18.75	0.16%	8.16	28.83	20.67	33.28%				73.3
Men	LLLT	24	Freq NMA	18.78		11.88	25.67	13.79		5.78	<0.0001	yes	75.1
Men	Minoxidil 2%	24	Bayesian NMA	10.54	0.76%	-5.81	26.32	32.13	34.14%				36.9
Men	Minoxidil 2%	24	Freq NMA	10.62		0.04	21.2	21.16		2	0.0491	yes	35.6
Men	Minoxidil 5%	24	Bayesian NMA	13.13	1.68%	2.39	22.4	20.01	27.09%				48.3
Men	Minoxidil 5%	24	Freq NMA	13.35		6.06	20.65	14.59		3.73	0.0003	yes	46.8
Men	Viviscal	24	Bayesian NMA	13.23	0.08%	-5.9	32.29	38.19	27.00%				48.5
Men	Viviscal	24	Freq NMA	13.24		-0.7	27.18	27.88		1.23	0.0627	no	48.2

Front. Med. 9:998623. doi: 10.3389/fmed.2022.998623

Section 3: Confidence in Network Meta-Analysis and Risk of Bias Results

Figure S1-5: CiNeMa Network Plots and Risk of Bias between Treatments

CiNeMA Network Plots and Risk of Bias between Treatments at 24 Weeks

Women

(A) **CiNeMa Network Plot**

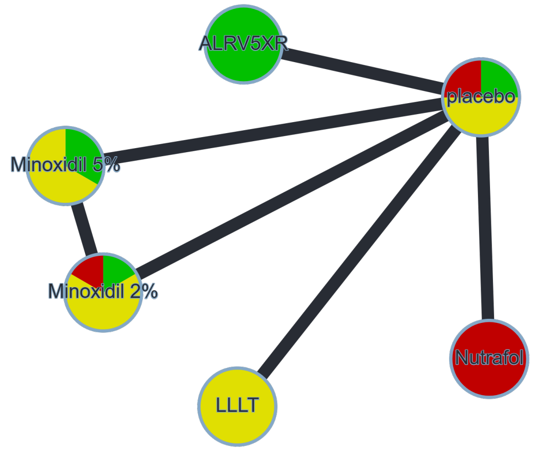

Men

(B) **CiNeMa Network Plot**

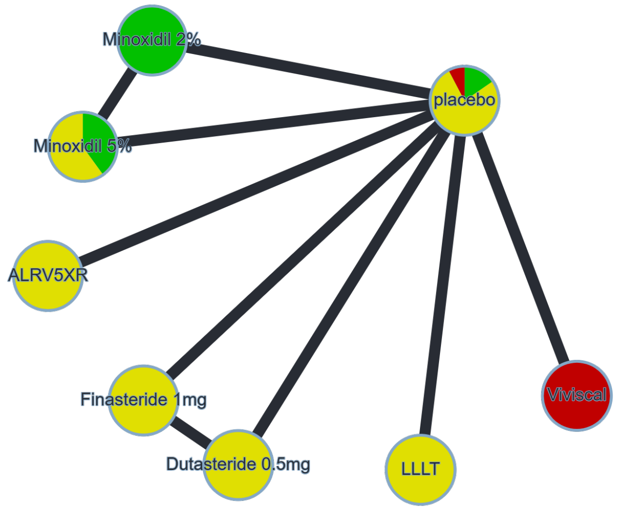

(C) **Women: CiNeMA Risk of Bias Chart**

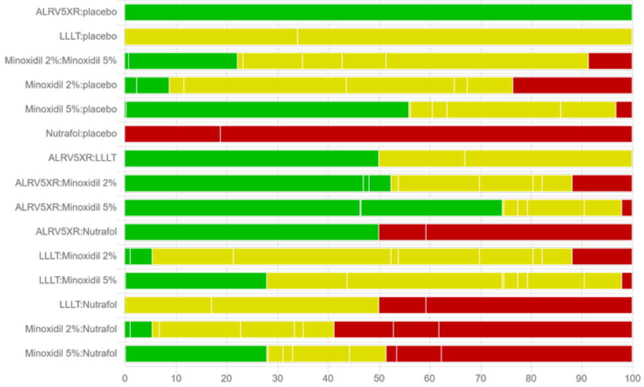

(D) **Men: CiNeMA Risk of Bias Chart**

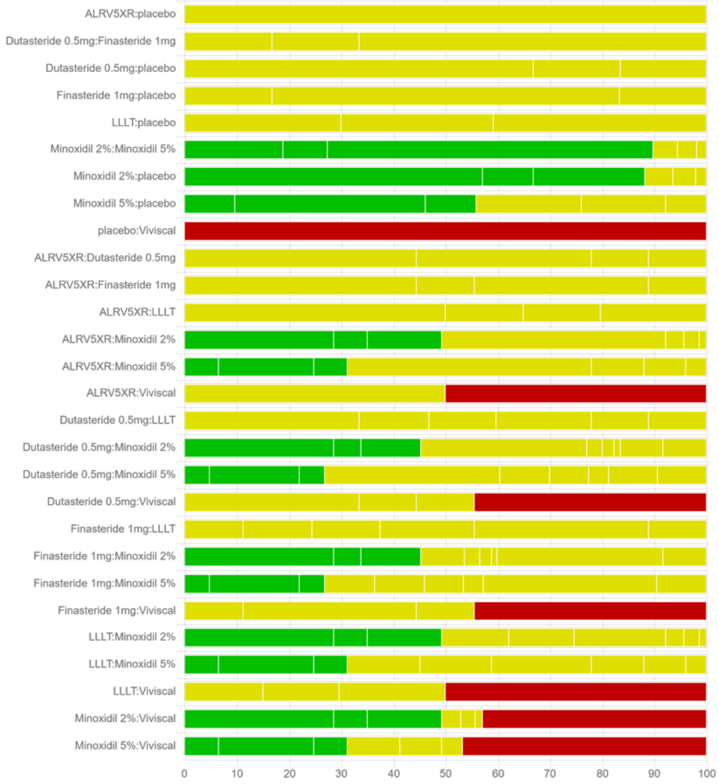

Legend: Green: Low Risk of bias, Yellow: Some Concerns, Red: High risk of bias.

Front. Med. 9:998623. doi: 10.3389/fmed.2022.998623

Table S1-4: CiNeMA Results

(A) Women at 24 Weeks

Comparison	Number of studies	Within-study bias	Reporting bias	Indirectness	Imprecision	Heterogeneity	Incoherence	Reason(s) for upgrading	Confidence rating	Reason(s) for downgrading
ALRV5XR:placebo	1	No concerns	Low risk	No concerns	No concerns	No concerns	No concerns	High magnitude of effect	High	[]
LLLT:placebo	2	Some concerns	Low risk	No concerns	No concerns	No concerns	No concerns	High magnitude of effect	High	["Within-study bias"]
Minoxidil 2%:Minoxidil 5%	1	Some concerns	Low risk	No concerns	No concerns	No concerns	No concerns		Moderate	["Within-study bias"]
Minoxidil 2%:placebo	5	Some concerns	Low risk	No concerns	No concerns	No concerns	No concerns		Moderate	["Within-study bias"]
Minoxidil 5%:placebo	2	No concerns	Low risk	No concerns	No concerns	No concerns	No concerns		High	[]
Nutrafol:placebo	2	Major concerns	Low risk	No concerns	No concerns	No concerns	No concerns		Low	["Within-study bias"]
ALRV5XR:LLLT	0	Some concerns	Low risk	No concerns	Some concerns	No concerns	No concerns		Low	["Within-study bias","Imprecision"]
ALRV5XR:Minoxidil 2%	0	Some concerns	Low risk	No concerns	No concerns	Some concerns	No concerns		Low	["Within-study bias","Heterogeneity"]
ALRV5XR:Minoxidil 5%	0	No concerns	Low risk	No concerns	No concerns	No concerns	No concerns		High	[]
ALRV5XR:Nutrafol	0	Some concerns	Low risk	No concerns	No concerns	No concerns	No concerns		Moderate	["Within-study bias"]
LLLT:Minoxidil 2%	0	Some concerns	Low risk	No concerns	No concerns	Some concerns	No concerns		Low	["Within-study bias","Heterogeneity"]
LLLT:Minoxidil 5%	0	Some concerns	Low risk	No concerns	No concerns	Some concerns	No concerns		Low	["Within-study bias","Heterogeneity"]
LLLT:Nutrafol	0	Major concerns	Low risk	No concerns	No concerns	No concerns	No concerns		Low	["Within-study bias"]
Minoxidil 2%:Nutrafol	0	Major concerns	Low risk	No concerns	No concerns	No concerns	No concerns		Low	["Within-study bias"]
Minoxidil 5%:Nutrafol	0	Some concerns	Low risk	No concerns	No concerns	No concerns	No concerns		Moderate	["Within-study bias"]

(B) Men at 24 Weeks

Comparison	Number of studies	Within-study bias	Reporting bias	Indirectness	Imprecision	Heterogeneity	Incoherence	Reason(s) for upgrading	Confidence rating	Reason(s) for downgrading
ALRV5XR:placebo	1	Some concerns	Low risk	No concerns	No concerns	No concerns	No concerns	High magnitude of effect	High	["Within-study bias"]
Dutasteride 0.5mg:Finasteride 1mg	1	Some concerns	Low risk	No concerns	Some concerns	No concerns	No concerns		Low	["Within-study bias","Imprecision"]
Dutasteride 0.5mg:placebo	1	Some concerns	Low risk	No concerns	No concerns	No concerns	No concerns	High magnitude of effect	High	["Within-study bias"]
Finasteride 1mg:placebo	1	Some concerns	Low risk	No concerns	No concerns	Some concerns	No concerns		Low	["Within-study bias","Heterogeneity"]
LLLT:placebo	3	Some concerns	Low risk	No concerns	No concerns	No concerns	No concerns	High magnitude of effect	High	["Within-study bias"]
Minoxidil 2%:Minoxidil 5%	1	No concerns	Low risk	No concerns	Some concerns	No concerns	No concerns		Moderate	["Imprecision"]
Minoxidil 2%:placebo	1	No concerns	Low risk	No concerns	No concerns	Some concerns	No concerns		Moderate	["Heterogeneity"]
Minoxidil 5%:placebo	4	No concerns	Low risk	No concerns	No concerns	No concerns	No concerns		High	[]
placebo:Viviscal	1	Major concerns	Low risk	No concerns	No concerns	Some concerns	No concerns		Very low	["Within-study bias","Heterogeneity"]
ALRV5XR:Dutasteride 0.5mg	0	Some concerns	Low risk	No concerns	Major concerns	No concerns	No concerns		Very low	["Within-study bias","Imprecision"]
ALRV5XR:Finasteride 1mg	0	Some concerns	Low risk	No concerns	Some concerns	Some concerns	No concerns		Very low	["Within-study bias","Imprecision","Heterogeneity"]
ALRV5XR:LLLT	0	Some concerns	Low risk	No concerns	Major concerns	No concerns	No concerns		Very low	["Within-study bias","Imprecision"]
ALRV5XR:Minoxidil 2%	0	Some concerns	Low risk	No concerns	Some concerns	Some concerns	No concerns		Very low	["Within-study bias","Imprecision","Heterogeneity"]
ALRV5XR:Minoxidil 5%	0	Some concerns	Low risk	No concerns	Some concerns	Some concerns	No concerns		Very low	["Within-study bias","Imprecision","Heterogeneity"]
ALRV5XR:Viviscal	0	Major concerns	Low risk	No concerns	Some concerns	Some concerns	No concerns		Very low	["Within-study bias","Imprecision","Heterogeneity"]
Dutasteride 0.5mg:LLLT	0	Some concerns	Low risk	No concerns	No concerns	Major concerns	No concerns		Very low	["Within-study bias","Heterogeneity"]
Dutasteride 0.5mg:Minoxidil 2%	0	Some concerns	Low risk	No concerns	Some concerns	No concerns	No concerns		Low	["Within-study bias","Imprecision"]
Dutasteride 0.5mg:Minoxidil 5%	0	Some concerns	Low risk	No concerns	Some concerns	Some concerns	No concerns		Very low	["Within-study bias","Imprecision","Heterogeneity"]
Dutasteride 0.5mg:Viviscal	0	Some concerns	Low risk	No concerns	Some concerns	Some concerns	No concerns		Very low	["Within-study bias","Imprecision","Heterogeneity"]
Finasteride 1mg:LLLT	0	Some concerns	Low risk	No concerns	Some concerns	No concerns	No concerns		Low	["Within-study bias","Imprecision"]
Finasteride 1mg:Minoxidil 2%	0	Some concerns	Low risk	No concerns	Some concerns	Some concerns	No concerns		Very low	["Within-study bias","Imprecision","Heterogeneity"]
Finasteride 1mg:Minoxidil 5%	0	Some concerns	Low risk	No concerns	Some concerns	Some concerns	No concerns		Very low	["Within-study bias","Imprecision","Heterogeneity"]
Finasteride 1mg:Viviscal	0	Some concerns	Low risk	No concerns	Major concerns	No concerns	No concerns		Very low	["Within-study bias","Imprecision"]
LLLT:Minoxidil 2%	0	Some concerns	Low risk	No concerns	Some concerns	No concerns	No concerns		Low	["Within-study bias","Imprecision"]
LLLT:Minoxidil 5%	0	Some concerns	Low risk	No concerns	Some concerns	No concerns	No concerns		Low	["Within-study bias","Imprecision"]
LLLT:Viviscal	0	Major concerns	Low risk	No concerns	Some concerns	Some concerns	No concerns		Very low	["Within-study bias","Imprecision","Heterogeneity"]
Minoxidil 2%:Viviscal	0	Some concerns	Low risk	No concerns	Major concerns	No concerns	No concerns		Very low	["Within-study bias","Imprecision"]
Minoxidil 5%:Viviscal	0	Some concerns	Low risk	No concerns	Major concerns	No concerns	No concerns		Very low	["Within-study bias","Imprecision"]

(C) Women at 12 Weeks

Comparison	Number of studies	Within-study bias	Reporting bias	Indirectness	Imprecision	Heterogeneity	Incoherence	Reason(s) for upgrading	Confidence rating	Reason(s) for downgrading
ALRV5XR:placebo	1	No concerns	Low risk	No concerns	No concerns	No concerns	No concerns		High	[]
LLLT:placebo	2	Some concerns	Low risk	No concerns	No concerns	No concerns	No concerns		Moderate	["Within-study bias"]
Minoxidil 2%:Minoxidil 5%	1	Some concerns	Low risk	No concerns	No concerns	No concerns	No concerns		Moderate	["Within-study bias"]
Minoxidil 2%:placebo	4	Some concerns	Low risk	No concerns	No concerns	No concerns	No concerns		Moderate	["Within-study bias"]
Minoxidil 5%:placebo	2	No concerns	Low risk	No concerns	No concerns	No concerns	No concerns		High	[]
Nutrafol:placebo	2	Major concerns	Low risk	No concerns	No concerns	No concerns	No concerns		Low	["Within-study bias"]
ALRV5XR:LLLT	0	Some concerns	Low risk	No concerns	Some concerns	No concerns	No concerns		Low	["Within-study bias","Imprecision"]
ALRV5XR:Minoxidil 2%	0	Some concerns	Low risk	No concerns	No concerns	Major concerns	No concerns		Very low	["Within-study bias","Heterogeneity"]
ALRV5XR:Minoxidil 5%	0	No concerns	Low risk	No concerns	No concerns	Major concerns	No concerns		Low	["Heterogeneity"]
ALRV5XR:Nutrafol	0	Some concerns	Low risk	No concerns	No concerns	Some concerns	No concerns		Low	["Within-study bias","Heterogeneity"]
LLLT:Minoxidil 2%	0	Some concerns	Low risk	No concerns	No concerns	No concerns	No concerns		Moderate	["Within-study bias"]
LLLT:Minoxidil 5%	0	Some concerns	Low risk	No concerns	No concerns	No concerns	No concerns		Moderate	["Within-study bias"]
LLLT:Nutrafol	0	Major concerns	Low risk	No concerns	No concerns	Some concerns	No concerns		Very low	["Within-study bias","Heterogeneity"]
Minoxidil 2%:Nutrafol	0	Major concerns	Low risk	No concerns	No concerns	No concerns	No concerns		Low	["Within-study bias"]
Minoxidil 5%:Nutrafol	0	Some concerns	Low risk	No concerns	No concerns	No concerns	No concerns		Moderate	["Within-study bias"]

(D) Men at 12 Weeks

Comparison	Number of studies	Within-study bias	Reporting bias	Indirectness	Imprecision	Heterogeneity	Incoherence	Reason(s) for upgrading	Confidence rating	Reason(s) for downgrading
ALRV5XR:placebo	1	Some concerns	Low risk	No concerns	No concerns	No concerns	No concerns		Moderate	["Within-study bias"]
Dutasteride 0.5mg:Finasteride 1mg	1	Some concerns	Low risk	No concerns	No concerns	No concerns	No concerns		Moderate	["Within-study bias"]
Dutasteride 0.5mg:placebo	1	Some concerns	Low risk	No concerns	No concerns	No concerns	No concerns	High magnitude of effect	High	["Within-study bias"]
Finasteride 1mg:placebo	1	Some concerns	Low risk	No concerns	No concerns	No concerns	No concerns		Moderate	["Within-study bias"]
LLLT:placebo	2	Some concerns	Low risk	No concerns	No concerns	No concerns	No concerns		Moderate	["Within-study bias"]
Minoxidil 2%:Minoxidil 5%	1	No concerns	Low risk	No concerns	No concerns	No concerns	No concerns		High	[]
Minoxidil 2%:placebo	1	No concerns	Low risk	No concerns	No concerns	No concerns	No concerns		High	[]
Minoxidil 5%:placebo	4	No concerns	Low risk	No concerns	No concerns	No concerns	No concerns	High magnitude of effect	High	[]
placebo:Viviscal	1	Major concerns	Low risk	No concerns	No concerns	Some concerns	No concerns		Very low	["Within-study bias","Heterogeneity"]
ALRV5XR:Dutasteride 0.5mg	0	Some concerns	Low risk	No concerns	Some concerns	No concerns	No concerns		Low	["Within-study bias","Imprecision"]
ALRV5XR:Finasteride 1mg	0	Some concerns	Low risk	No concerns	No concerns	Major concerns	No concerns		Very low	["Within-study bias","Heterogeneity"]
ALRV5XR:LLLT	0	Some concerns	Low risk	No concerns	No concerns	Some concerns	No concerns		Low	["Within-study bias","Heterogeneity"]
ALRV5XR:Minoxidil 2%	0	Some concerns	Low risk	No concerns	No concerns	Some concerns	No concerns		Low	["Within-study bias","Heterogeneity"]
ALRV5XR:Minoxidil 5%	0	Some concerns	Low risk	No concerns	Some concerns	No concerns	No concerns		Low	["Within-study bias","Imprecision"]
ALRV5XR:Viviscal	0	Major concerns	Low risk	No concerns	Some concerns	No concerns	No concerns		Very low	["Within-study bias","Imprecision"]
Dutasteride 0.5mg:LLLT	0	Some concerns	Low risk	No concerns	No concerns	Some concerns	No concerns		Low	["Within-study bias","Heterogeneity"]
Dutasteride 0.5mg:Minoxidil 2%	0	Some concerns	Low risk	No concerns	No concerns	Some concerns	No concerns		Low	["Within-study bias","Heterogeneity"]
Dutasteride 0.5mg:Minoxidil 5%	0	Some concerns	Low risk	No concerns	No concerns	No concerns	No concerns		Moderate	["Within-study bias"]
Dutasteride 0.5mg:Viviscal	0	Some concerns	Low risk	No concerns	No concerns	Some concerns	No concerns		Low	["Within-study bias","Heterogeneity"]
Finasteride 1mg:LLLT	0	Some concerns	Low risk	No concerns	No concerns	No concerns	No concerns		Moderate	["Within-study bias"]
Finasteride 1mg:Minoxidil 2%	0	Some concerns	Low risk	No concerns	No concerns	No concerns	No concerns		Moderate	["Within-study bias"]
Finasteride 1mg:Minoxidil 5%	0	Some concerns	Low risk	No concerns	No concerns	Some concerns	No concerns		Low	["Within-study bias","Heterogeneity"]
Finasteride 1mg:Viviscal	0	Some concerns	Low risk	No concerns	Some concerns	No concerns	No concerns		Low	["Within-study bias","Imprecision"]
LLLT:Minoxidil 2%	0	Some concerns	Low risk	No concerns	No concerns	No concerns	No concerns		Moderate	["Within-study bias"]
LLLT:Minoxidil 5%	0	Some concerns	Low risk	No concerns	No concerns	Some concerns	No concerns		Low	["Within-study bias","Heterogeneity"]
LLLT:Viviscal	0	Major concerns	Low risk	No concerns	Some concerns	No concerns	No concerns		Very low	["Within-study bias","Imprecision"]
Minoxidil 2%:Viviscal	0	Some concerns	Low risk	No concerns	Some concerns	No concerns	No concerns		Low	["Within-study bias","Imprecision"]
Minoxidil 5%:Viviscal	0	Some concerns	Low risk	No concerns	No concerns	Some concerns	No concerns		Low	["Within-study bias","Heterogeneity"]

Figure S1-6: Cochrane Risk of Bias

(A) Cochrane Risk of Bias by Study

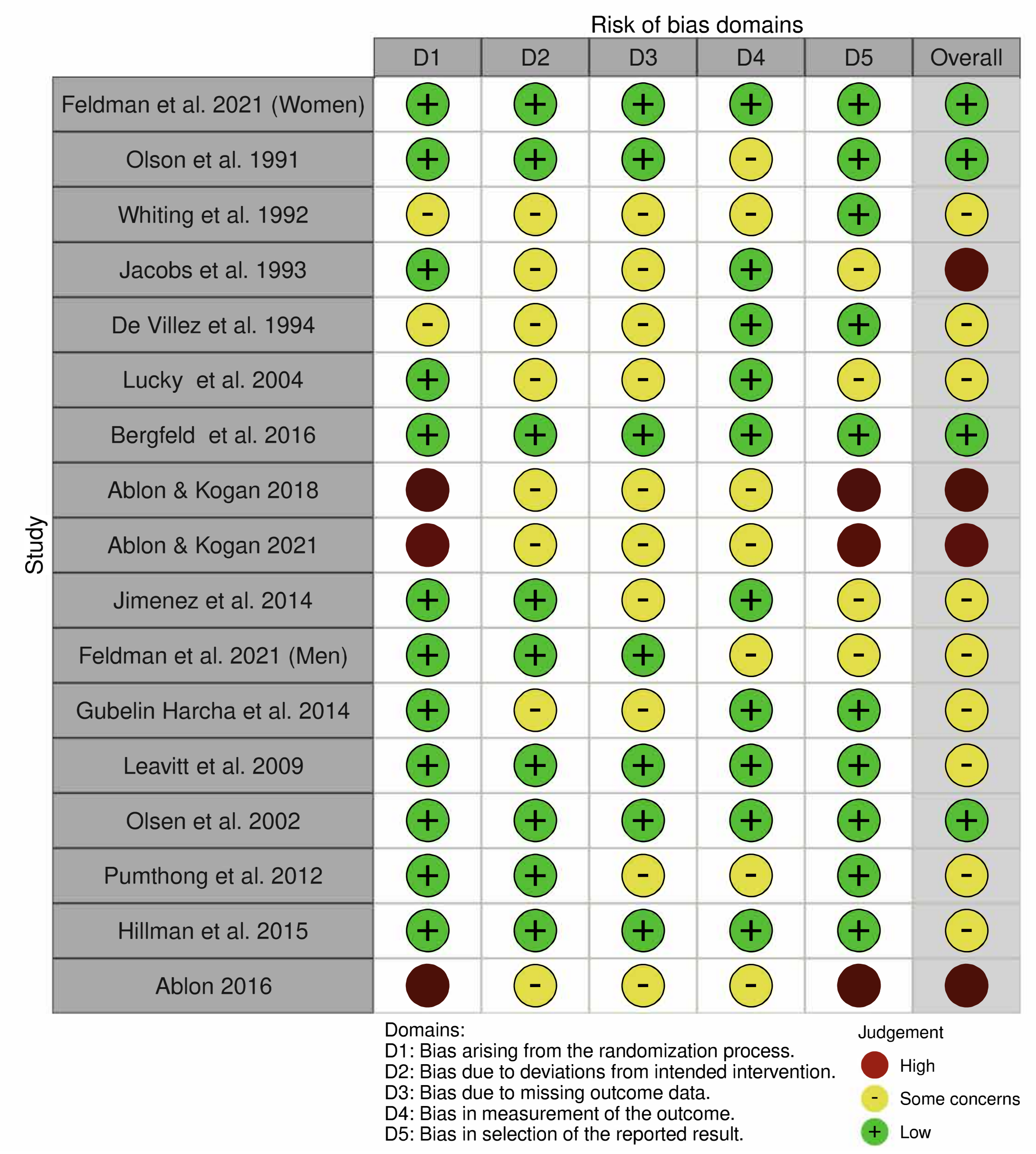

(B) Cochrane Summary Risk of Bias

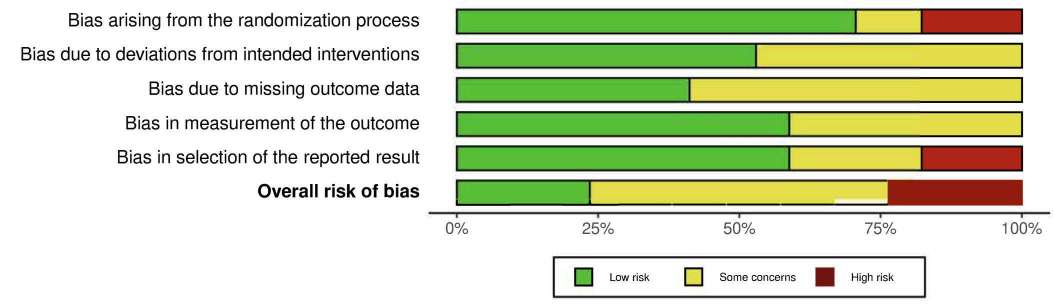

Table S1-5: Study Conflicts of Interest

Author, Year	Sponsor / owner of investigated treatment	Treatment	Sponsor Brand	Sponsor Affiliated Authors	Sponsor Generated Data
**Women**					
Ablon et al, 2018[45]	Unilever (Neutraceutical Wellness Inc.)	Nutrafol	Nutrafol	Yes	Unknown
Ablon et al, 2021[46]	Unilever (Neutraceutical Wellness Inc.)	Nutrafol	Nutrafol	Yes	Unknown
Bergfeld et al, 2016[44]	Johnson & Johnson Consumer Companies*	Minoxidil 5%	Rogaine	Yes	No
DeVillez et al, 1994[42]	The Upjohn Company	Minoxidil 2%	Rogaine	Yes	Yes
Feldman et al, 2021[12]	Arbor Life Labs	ALRV5XR	Replenology	Yes	No
Jacobs et al, 1993[41]	The Upjohn Company	Minoxidil 2%	Rogaine	Yes	Yes
Jimenez et al, 2014[49]***	Lexington International, LLC	LLLT (comb)	HairMax	No	No
Lucky et al, 2004[43]	Pfizer Inc (formerly Pharmacia Corporation, formerly The Upjohn Company)	Minoxidil 2% & 5%	Rogaine	No	No
Olsen et al, 1991[39]	The Upjohn Company	Minoxidil 2%	Rogaine	No	Unknown
Whiting et al, 1992[40]	The Upjohn Company	Minoxidil 2%	Rogaine	No	Yes
**Men**					
Ablon, 2016[53]	Church and Dwight (Lifes2good, Inc., Chicago, IL.)	Viviscal	Viviscal	No	No
Feldman et al, 2021[13]	Arbor Life Labs	ALRV5XR	Replenology	Yes	No
Gubelin Harcha et al, 2014[48]	GlaxoSmithKline	Dutasteride<br>Finasteride	Avodart<br>N/A (Generic)**	Yes	Yes
Hillmann et al, 2015[52]	Johnson & Johnson Consumer Companies*	Minoxidil 5%	Rogaine	Yes	No
Jimenez et al, 2014[47]***	Lexington International, LLC	LLLT (comb)	HairMax	No	No
Leavitt et al, 2009[49]	Lexington International, LLC	LLLT (comb)	HairMax	Yes	Unknown
Olsen et al, 2002[50]	Pharmacia Corporation (The Upjohn Corporation) *	Minoxidil 2% & 5%	Rogaine	Yes	Yes
Pumthong et al, 2012[51]	Unknown	Minoxidil 5%	Unknown	Unknown	Unknown

* *Johnson & Johnson acquired the Rogaine brand in 2006.* [44, 52]

** *Finasteride is also branded as Propecia and Proscar by Merck. Merck was not involved in this study.* [48]

*** This study is listed in both women and men groupings.[47]

Front. Med. 9:998623. doi: 10.3389/fmed.2022.998623

Section 4: Continuous Outcomes Data and Additional Results

Table S1-6: Extracted Continuous Outcomes Data and Weekly imputed results in terminal hair regrowth in TH/ cm^2 of AGA treatments in women by week

Extracted Data of Terminal Hair Regrowth from Eligible Studies

Women				Placebo			Treatment			Efficacy	
				Population	BL TH	Change from BL	Population	BL TH	Change from BL	Absolute	Relative
#	Treatment	Study	Week	N	TH/cm^2	TH/cm^2	N	TH/cm^2	TH/cm^2	TH/cm^2	%
1	ALRV5XR	Feldman et al. 2021	12	21	141.6	3.1	18	139.1	16.1	12.9	9.3%
2	ALRV5XR	Feldman et al. 2021	24	21	141.6	3.9	18	139.1	33.9	30.1	21.7%
3	LLLT	Jimenez et al. 2014 (12-beam)	16	18	168.4	-0.8	39	142.2	11.9	12.7	8.8%
4	LLLT	Jimenez et al. 2014 (12-beam)	26	18	168.4	3.0	39	142.2	20.6	17.6	12.7%
5	LLLT	Jimenez et al. 2014 (9-beam)	16	22	155.7	1.3	43	162.6	14.8	13.5	8.3%
6	LLLT	Jimenez et al. 2014 (9-beam)	26	21	155.7	2.8	42	162.6	20.2	17.4	10.6%
7	Minoxidil 2%	De Villez et al. 1994	4	122	138.6	3.8	122	140.4	3.6	-0.2	-0.2%
8	Minoxidil 2%	De Villez et al. 1994	8	124	138.6	5.9	125	140.4	16.3	10.4	7.4%
9	Minoxidil 2%	De Villez et al. 1994	12	121	138.6	9.2	121	140.4	25.5	16.3	11.5%
10	Minoxidil 2%	De Villez et al. 1994	16	126	138.6	8.4	126	140.4	26.4	18.0	12.7%
11	Minoxidil 2%	De Villez et al. 1994	20	123	138.6	11.8	120	140.4	25.3	13.5	9.5%
12	Minoxidil 2%	De Villez et al. 1994	24	123	138.6	9.9	125	140.4	22.2	12.3	8.7%
13	Minoxidil 2%	De Villez et al. 1994	28	121	138.6	15.7	119	140.4	26.6	10.9	7.6%
14	Minoxidil 2%	De Villez et al. 1994	32	128	138.6	10.1	128	140.4	22.7	12.6	8.9%
15	Minoxidil 2%	Jacobs et al. 1993	4	139	139.8	15.5	154	136.7	17.2	1.7	1.5%
16	Minoxidil 2%	Jacobs et al. 1993	8	138	139.8	8.9	154	136.7	20.6	11.7	8.7%
17	Minoxidil 2%	Jacobs et al. 1993	12	139	139.8	19.8	155	136.7	35.7	15.9	12.0%
18	Minoxidil 2%	Jacobs et al. 1993	16	139	139.8	10.2	153	136.7	28.4	18.2	13.5%
19	Minoxidil 2%	Jacobs et al. 1993	20	137	139.8	19.1	152	136.7	34.5	15.4	11.6%
20	Minoxidil 2%	Jacobs et al. 1993	24	137	139.8	14.0	147	136.7	26.1	12.1	9.1%
21	Minoxidil 2%	Jacobs et al. 1993	28	135	139.8	11.9	153	136.7	25.3	13.4	10.0%
22	Minoxidil 2%	Jacobs et al. 1993	32	139	139.8	19.1	155	136.7	33.1	14.0	10.6%
23	Minoxidil 2%	Olsen et al. 1991	32	14	154.2	20.6	14	160.1	50.1	29.5	17.9%
24	Minoxidil 2%	Whiting et al. 1992	8	16	161.0	7.0	16	169.0	20.0	13.0	7.5%
25	Minoxidil 2%	Whiting et al. 1992	16	16	161.0	10.0	16	169.0	21.0	11.0	6.2%
26	Minoxidil 2%	Whiting et al. 1992	24	20	161.0	17.0	14	169.0	21.0	4.0	1.9%
27	Minoxidil 2%	Whiting et al. 1992	32	13	161.0	20.0	15	169.0	28.0	8.0	4.1%
28	Minoxidil 2%	Lucky et al. 2004	8	51	138.4	11.1	108	150.4	18.3	7.2	4.1%
29	Minoxidil 2%	Lucky et al. 2004	16	51	138.4	20.0	108	150.4	35.9	15.9	9.4%
30	Minoxidil 2%	Lucky et al. 2004	32	51	138.4	15.2	108	150.4	26.7	11.5	6.8%
31	Minoxidil 2%	Lucky et al. 2004	48	51	138.4	9.4	108	150.4	20.7	11.3	7.0%
32	Minoxidil 5%	Lucky et al. 2004	8	51	138.4	11.1	101	141.3	21.7	10.6	7.3%
33	Minoxidil 5%	Lucky et al. 2004	16	51	138.4	20.0	101	141.3	36.0	16.0	11.0%
34	Minoxidil 5%	Lucky et al. 2004	32	51	138.4	15.2	101	141.3	27.1	11.9	8.2%
35	Minoxidil 5%	Lucky et al. 2004	48	51	138.4	9.4	101	141.3	24.5	15.1	10.5%
36	Minoxidil 5%	Bergfeld et al. 2016	12	168	152.7	5.3	172	158.6	16.4	11.2	6.9%
37	Minoxidil 5%	Bergfeld et al. 2016	24	165	152.7	4.3	159	158.6	13.4	9.1	5.6%
38	Nutrafol	Ablon & Kogan 2018	12.9	14	136.5	1.0	26	141.7	9.7	8.7	6.1%
39	Nutrafol	Ablon & Kogan 2018	25.7	14	136.5	4.8	26	141.7	14.7	9.9	6.9%
40	Nutrafol	Ablon & Kogan 2021	12.9	27	69.2	-0.3	33	68.1	2.9	3.2	4.7%
41	Nutrafol	Ablon & Kogan 2021	25.7	27	69.2	0.3	33	68.1	7.0	6.7	9.8%

Front. Med. 9:998623. doi: 10.3389/fmed.2022.998623

Table S1-7: Imputed results in terminal hair regrowth of AGA treatments in women by week in TH/ cm^2

WOMEN: Imputed Weighted Average Efficacy by Treatment by Week (TH/cm^2)

Week	0	1	2	3	4	5	6	7	8	9	10	11	12	13	14	15	16	17	18	19	20	21	22	23	24
**ALRV5XR**	0	1.1	2.2	3.2	4.3	5.4	6.5	7.5	8.6	9.7	10.8	11.8	12.9	14.4	15.8	17.2	18.6	20.1	21.5	22.9	24.4	25.8	27.2	28.7	30.1
**LLLT**	0	0.8	1.6	2.4	3.3	4.1	4.9	5.7	6.5	7.3	8.2	9.0	9.8	10.6	11.4	12.2	13.1	13.5	14.0	14.4	14.8	15.3	15.7	16.2	16.6
**Minoxidil 2%**	0	0.4	0.8	1.3	1.7	3.9	6.1	8.3	10.5	11.6	12.7	13.8	14.9	15.8	16.6	17.5	18.4	17.5	16.7	15.9	15.1	14.6	14.1	13.7	13.2
**Minoxidil 5%**	0	1.2	2.4	3.6	4.8	6.0	7.2	8.4	9.6	10.6	11.5	12.4	13.4	13.7	14.0	14.3	14.7	14.5	14.3	14.1	13.9	13.6	13.4	13.2	13.0
**Nutrafol**	0	0.4	0.9	1.3	1.8	2.2	2.7	3.1	3.6	4.0	4.5	4.9	5.4	5.8	6.0	6.2	6.4	6.7	6.9	7.1	7.3	7.5	7.7	8.0	8.2

Week	25	26	27	28	29	30	31	32	33	34	35	36	37	38	39	40	41	42	43	44	45	46	47	48
**ALRV5XR**																								
**LLLT**	17.1	17.5																						
**Minoxidil 2%**	13.1	13.0	12.9	12.7	12.9	13.1	13.2	13.4																
**Minoxidil 5%**																								
**Nutrafol**	8.4																							

Notes: Imputed results are treatment efficacy at each week measured as difference between the weighted average by population of each group within each treatment cohort. Weeks are whole weeks (fractional weeks are rounded down to the nearest integer) and the full data point result for the fractional week is assigned to the rounded down week.

Front. Med. 9:998623. doi: 10.3389/fmed.2022.998623

Table S1-8: Imputed results in terminal hair regrowth of AGA treatments in women by week in Percent (%)

WOMEN: Imputed Weighted Average Efficacy by Treatment by Week (%)

Week	0	1	2	3	4	5	6	7	8	9	10	11	12	13	14	15	16	17	18	19	20	21	22	23	24
**ALRV5XR**	0%	0.8%	1.6%	2.3%	3.1%	3.9%	4.7%	5.4%	6.2%	7.0%	7.8%	8.6%	9.3%	10.4%	11.4%	12.4%	13.4%	14.5%	15.5%	16.5%	17.6%	18.6%	19.6%	20.6%	21.7%
**LLLT**	0%	0.5%	1.1%	1.6%	2.1%	2.7%	3.2%	3.7%	4.3%	4.8%	5.3%	5.8%	6.4%	6.9%	7.4%	8.0%	8.5%	8.8%	9.1%	9.4%	9.8%	10.1%	10.4%	10.7%	11.0%
**Minoxidil 2%**	0%	0.3%	0.6%	0.8%	1.1%	2.7%	4.2%	5.7%	7.3%	8.1%	8.8%	9.6%	10.4%	11.0%	11.5%	12.1%	12.7%	12.1%	11.5%	10.9%	10.4%	10.0%	9.7%	9.4%	9.1%
**Minoxidil 5%**	0%	0.8%	1.6%	2.4%	3.2%	4.0%	4.8%	5.6%	6.4%	7.0%	7.6%	8.2%	8.8%	9.1%	9.3%	9.5%	9.8%	9.6%	9.5%	9.4%	9.2%	9.1%	9.0%	8.8%	8.7%
**Nutrafol**	0%	0.4%	0.8%	1.3%	1.7%	2.1%	2.5%	3.0%	3.4%	3.8%	4.2%	4.6%	5.1%	5.5%	5.7%	6.0%	6.3%	6.5%	6.8%	7.1%	7.3%	7.6%	7.9%	8.1%	8.4%

Week	25	26	27	28	29	30	31	32	33	34	35	36	37	38	39	40	41	42	43	44	45	46	47	48
**ALRV5XR**																								
**LLLT**	11.3%	11.6%																						
**Minoxidil 2%**	9.0%	8.9%	8.8%	8.7%	8.8%	9.0%	9.1%	9.2%																
**Minoxidil 5%**																								
**Nutrafol**	8.6%																							

Notes: Results are treatment efficacy at each week measured as percent difference between the population weighted average of each group within each treatment cohort. Weeks are whole weeks (fractional weeks are rounded down to the nearest integer) and the full data point result for the fractional week is assigned to the rounded down week.

Figure S1-6 Continuous Outcomes of regrowth of AGA treatments in women by week

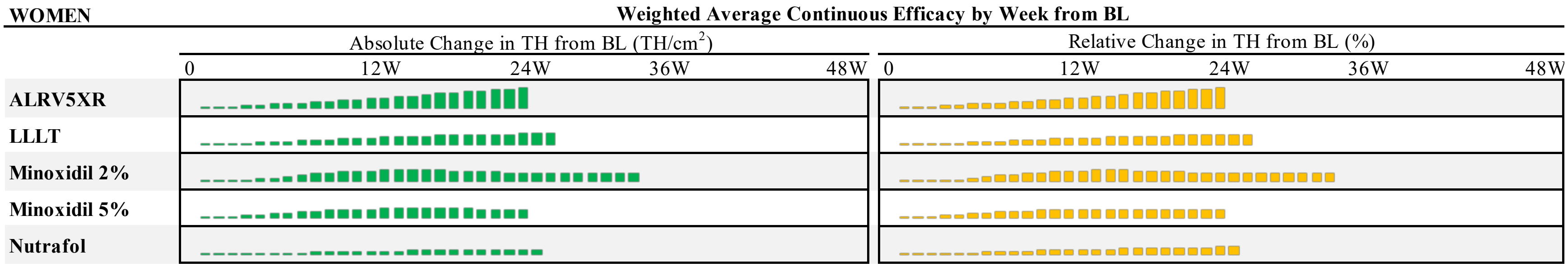

Front. Med. 9:998623. doi: 10.3389/fmed.2022.998623

Figure S1-7: Continuous Outcomes in Women for Treatments with Multiple Studies

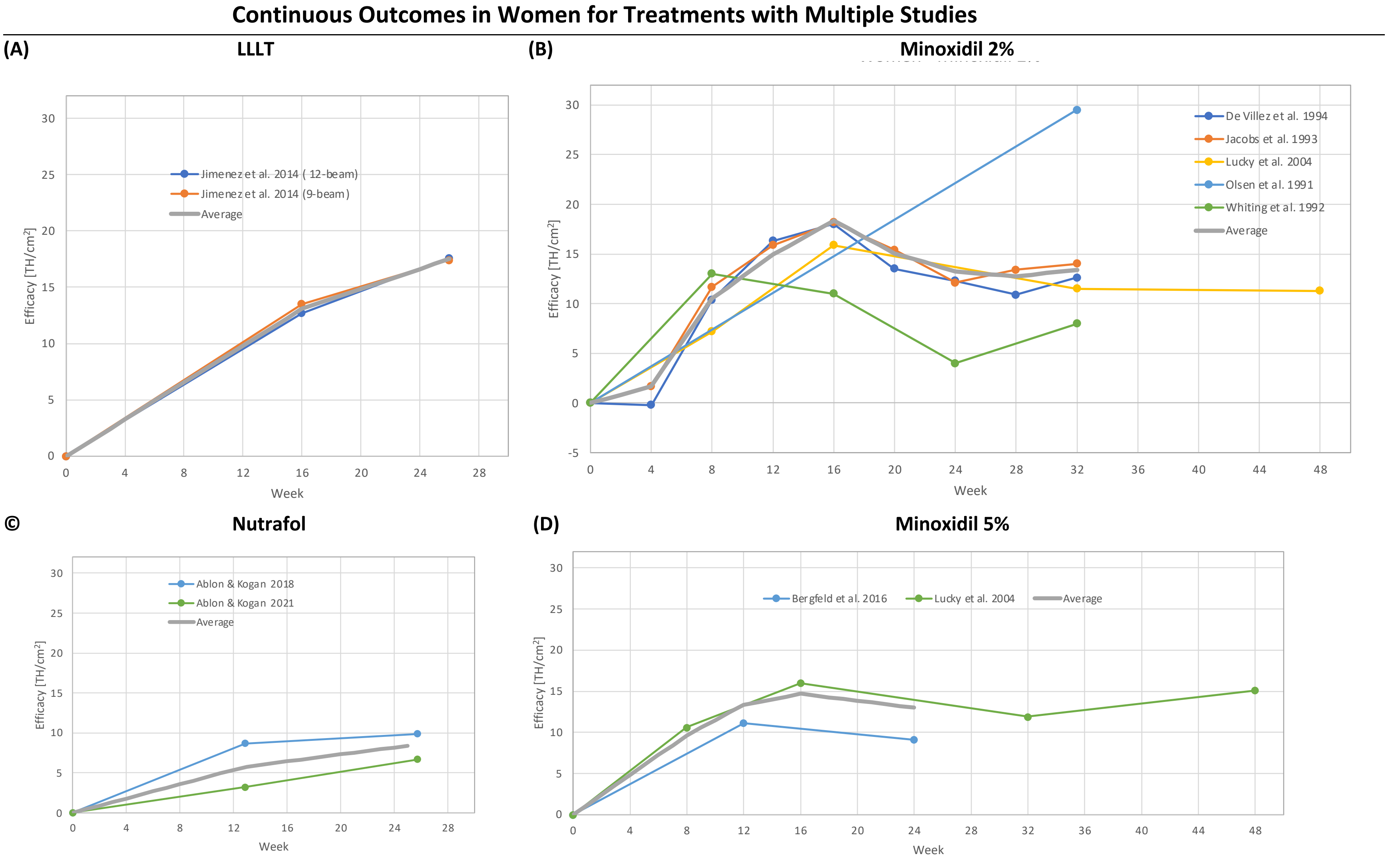

Table S1-9: Extracted Continuous Outcomes Data and Weekly imputed results in terminal hair regrowth per cm^2 of AGA treatments in men by week

Extracted Data of Terminal Hair Regrowth from Eligible Studies

Men				Placebo			Treatment			Efficacy	
				Population	BL TH	Change from BL	Population	BL TH	Change from BL	Absolute	Relative
#	Treatment	Study	Week	N	TH/cm^2	TH/cm^2	N	TH/cm^2	TH/cm^2	TH/cm^2	%
1	ALRV5XR	Feldman et al. 2021	12	11	141.6	-3.0	11	147.0	8.0	11.0	7.5%
2	ALRV5XR	Feldman et al. 2021	24	11	141.6	-5.8	11	147.0	15.2	21.0	14.4%
3	Dutasteride 0.5mg	Gubelin Harcha et al. 2014	12	157	148.0	-0.4	153	149.0	17.1	17.5	11.7%
4	Dutasteride 0.5mg	Gubelin Harcha et al. 2014	24	157	148.0	-0.3	153	149.0	18.1	18.4	12.4%
5	Finasteride 1mg	Gubelin Harcha et al. 2014	12	157	148.0	-0.4	141	148.0	10.8	11.2	7.6%
6	Finasteride 1mg	Gubelin Harcha et al. 2014	24	157	148.0	-0.3	141	148.0	12.1	12.4	8.4%
7	LLLT	Jimenez et al. 2014 (7-beam)	16	14	216.6	2.8	24	211.5	17.7	14.9	7.1%
8	LLLT	Jimenez et al. 2014 (7-beam)	26	14	216.6	1.6	24	211.5	18.4	16.8	8.0%
9	LLLT	Jimenez et al. 2014 (9-beam)	16	22	171.4	4.4	21	163.3	20.4	16.0	9.9%
10	LLLT	Jimenez et al. 2014 (9-beam)	26	21	171.4	9.4	21	163.3	20.9	11.5	7.3%
11	LLLT	Jimenez et al. 2014 (12-beam)	16	22	171.4	4.4	22	151.5	23.5	19.1	12.9%
12	LLLT	Jimenez et al. 2014 (12-beam)	26	21	171.4	9.4	19	151.5	25.7	16.3	11.5%
13	LLLT	Leavitt et al. 2009 (9-beam)	26	39	120.7	-8.9	71	122.9	17.3	26.2	21.5%
14	Minoxidil 2%	Olsen et al. 2002	8	71	152.4	14.5	141	143.6	25.0	10.5	7.9%
15	Minoxidil 2%	Olsen et al. 2002	16	71	152.4	15.3	141	143.6	29.8	14.5	10.7%
16	Minoxidil 2%	Olsen et al. 2002	32	71	152.4	7.7	141	143.6	22.5	14.8	10.6%
17	Minoxidil 2%	Olsen et al. 2002	48	71	152.4	3.9	139	143.6	12.7	8.8	6.3%
18	Minoxidil 5%	Olsen et al. 2002	8	71	152.4	14.5	139	151.1	29.7	15.2	10.1%
19	Minoxidil 5%	Olsen et al. 2002	16	71	152.4	15.3	139	151.1	35.7	20.4	13.6%
20	Minoxidil 5%	Olsen et al. 2002	32	71	152.4	7.7	139	151.1	29.0	21.3	14.1%
21	Minoxidil 5%	Olsen et al. 2002	48	71	152.4	3.9	141	151.1	18.6	14.7	9.8%
22	Minoxidil 5%	Hillmann et al. 2015 (Fron-temp)	16	34	217.2	4.5	30	208.3	27.7	23.2	11.2%
23	Minoxidil 5%	Hillmann et al. 2015 (Fron-temp)	24	34	217.2	-1.5	31	208.3	7.8	9.3	4.4%
24	Minoxidil 5%	Hillmann et al. 2015 (Vertex)	16	31	215.6	5.9	31	208.8	25.6	19.7	9.5%
25	Minoxidil 5%	Hillmann et al. 2015 (Vertex)	24	31	215.6	0.4	32	208.8	8.7	8.3	4.0%
26	Minoxidil 5%	Pumthong et al. 2012	4	22	178.6	6.9	22	173.3	8.2	1.3	0.9%
27	Minoxidil 5%	Pumthong et al. 2012	9	22	178.6	10.8	22	173.3	24.0	13.3	7.8%
28	Minoxidil 5%	Pumthong et al. 2012	13	22	178.6	15.0	22	173.3	23.7	8.8	5.3%
29	Minoxidil 5%	Pumthong et al. 2012	17	22	178.6	16.6	22	173.3	21.9	5.4	3.4%
30	Minoxidil 5%	Pumthong et al. 2012	22	22	178.6	24.0	22	173.3	27.2	3.3	2.3%
31	Minoxidil 5%	Pumthong et al. 2012	26	22	178.6	20.4	22	173.3	31.3	10.9	6.6%
32	Viviscal	Ablon 2016	12.9	30	104.7	-3.0	30	122.0	5.7	8.7	7.5%
33	Viviscal	Ablon 2016	25.7	30	104.7	-5.6	30	122.0	8.4	13.9	12.2%

Table S1-10: Imputed results in terminal hair regrowth of AGA treatments in men by week per cm^2

MEN: Imputed Weighted Average Efficacy by Treatment by Week (TH/cm^2)

Week	0	1	2	3	4	5	6	7	8	9	10	11	12	13	14	15	16	17	18	19	20	21	22	23	24
**ALRV5XR**	0	0.9	1.8	2.7	3.7	4.6	5.5	6.4	7.3	8.2	9.1	10.0	11.0	11.8	12.6	13.5	14.3	15.1	16.0	16.8	17.7	18.5	19.3	20.2	21.0
**Dutasteride 0.5mg**	0	1.5	2.9	4.4	5.8	7.3	8.8	10.2	11.7	13.1	14.6	16.0	17.5	17.6	17.7	17.7	17.8	17.9	18.0	18.0	18.1	18.2	18.3	18.3	18.4
**Finasteride 1mg**	0	0.9	1.9	2.8	3.7	4.7	5.6	6.5	7.5	8.4	9.3	10.3	11.2	11.3	11.4	11.5	11.6	11.7	11.8	11.9	12.0	12.1	12.2	12.3	12.4
**LLLT**	0	1.0	1.9	2.9	3.8	4.8	5.7	6.7	7.6	8.6	9.5	10.5	11.4	12.4	13.3	14.3	15.2	15.5	15.8	16.2	16.5	16.8	17.2	17.5	17.8
**Minoxidil 2%**	0	1.3	2.6	3.9	5.3	6.6	7.9	9.2	10.5	11.0	11.5	12.0	12.5	13.0	13.5	14.0	14.5	14.5	14.5	14.6	14.6	14.6	14.6	14.6	14.7
**Minoxidil 5%**	0	1.8	3.6	5.4	7.2	9.2	11.2	13.3	15.4	16.3	16.9	17.5	18.1	18.7	19.4	20.0	20.7	20.2	19.7	19.2	18.6	18.1	17.7	17.5	17.3
**Viviscal**	0	0.7	1.4	2.0	2.7	3.4	4.1	4.7	5.4	6.1	6.8	7.4	8.1	8.7	9.2	9.6	10.0	10.4	10.8	11.2	11.6	12.0	12.4	12.8	13.2

Week	25	26	27	28	29	30	31	32	33	34	35	36	37	38	39	40	41	42	43	44	45	46	47	48
**ALRV5XR**																								
**Dutasteride 0.5mg**																								
**Finasteride 1mg**																								
**LLLT**	18.2	18.5																						
**Minoxidil 2%**	14.7	14.7	14.7	14.7	14.7	14.8	14.8	14.8	14.4	14.1	13.7	13.3	12.9	12.6	12.2	11.8	11.4	11.1	10.7	10.3	9.9	9.6	9.2	8.8
**Minoxidil 5%**																								
**Viviscal**	13.6																							

Notes: Imputed results are treatment efficacy at each week measured as difference between the weighted average by population of each group within each treatment cohort. Weeks are whole weeks (fractional weeks are rounded down to the nearest integer) and the full data point result for the fractional week is assigned to the rounded down week.

Table S1-11: Imputed results in terminal hair regrowth of AGA treatments in men by week in Percent (%)

MEN: Imputed Weighted Average Efficacy by Week (%) for each Treatment

Week	0	1	2	3	4	5	6	7	8	9	10	11	12	13	14	15	16	17	18	19	20	21	22	23	24
**ALRV5XR**	0%	0.6%	1.3%	1.9%	2.5%	3.1%	3.8%	4.4%	5.0%	5.6%	6.3%	6.9%	7.5%	8.1%	8.7%	9.3%	9.8%	10.4%	11.0%	11.6%	12.1%	12.7%	13.3%	13.9%	14.4%
**Dutasteride 0.5mg**	0%	1.0%	2.0%	2.9%	3.9%	4.9%	5.9%	6.9%	7.8%	8.8%	9.8%	10.8%	11.7%	11.8%	11.8%	11.9%	11.9%	12.0%	12.0%	12.1%	12.1%	12.2%	12.2%	12.3%	12.4%
**Finasteride 1mg**	0%	0.6%	1.3%	1.9%	2.5%	3.2%	3.8%	4.4%	5.0%	5.7%	6.3%	6.9%	7.6%	7.6%	7.7%	7.8%	7.8%	7.9%	8.0%	8.0%	8.1%	8.2%	8.2%	8.3%	8.4%
**LLLT**	0%	0.7%	1.3%	2.0%	2.7%	3.4%	4.0%	4.7%	5.4%	6.1%	6.7%	7.4%	8.1%	8.7%	9.4%	10.1%	10.8%	11.1%	11.4%	11.7%	12.0%	12.3%	12.6%	12.9%	13.2%
**Minoxidil 2%**	0%	1.0%	2.0%	3.0%	3.9%	4.9%	5.9%	6.9%	7.9%	8.2%	8.6%	9.0%	9.3%	9.7%	10.0%	10.4%	10.7%	10.7%	10.7%	10.7%	10.7%	10.7%	10.7%	10.7%	10.7%
**Minoxidil 5%**	0%	1.1%	2.3%	3.4%	4.6%	5.8%	7.1%	8.4%	9.7%	10.2%	10.6%	11.0%	11.3%	11.7%	12.1%	12.5%	12.8%	12.6%	12.3%	12.0%	11.8%	11.5%	11.3%	11.2%	11.1%
**Viviscal**	0%	0.6%	1.2%	1.8%	2.3%	2.9%	3.5%	4.1%	4.7%	5.3%	5.9%	6.4%	7.0%	7.6%	7.9%	8.3%	8.7%	9.0%	9.4%	9.8%	10.1%	10.5%	10.8%	11.2%	11.6%

Week	25	26	27	28	29	30	31	32	33	34	35	36	37	38	39	40	41	42	43	44	45	46	47	48
**ALRV5XR**																								
**Dutasteride 0.5mg**																								
**Finasteride 1mg**																								
**LLLT**	13.5%	13.8%																						
**Minoxidil 2%**	10.7%	10.7%	10.6%	10.6%	10.6%	10.6%	10.6%	10.6%	10.3%	10.1%	9.8%	9.5%	9.3%	9.0%	8.7%	8.5%	8.2%	7.9%	7.6%	7.4%	7.1%	6.8%	6.6%	6.3%
**Minoxidil 5%**																								
**Viviscal**	11.9%																							

Notes: Results are treatment efficacy at each week measured as percent difference between the population weighted average of each group within each treatment cohort. Weeks are whole weeks (fractional weeks are rounded down to the nearest integer) and the full data point result for the fractional week is assigned to the rounded down week.

Figure S1-8 Continuous Outcomes of regrowth of AGA treatments in men by week

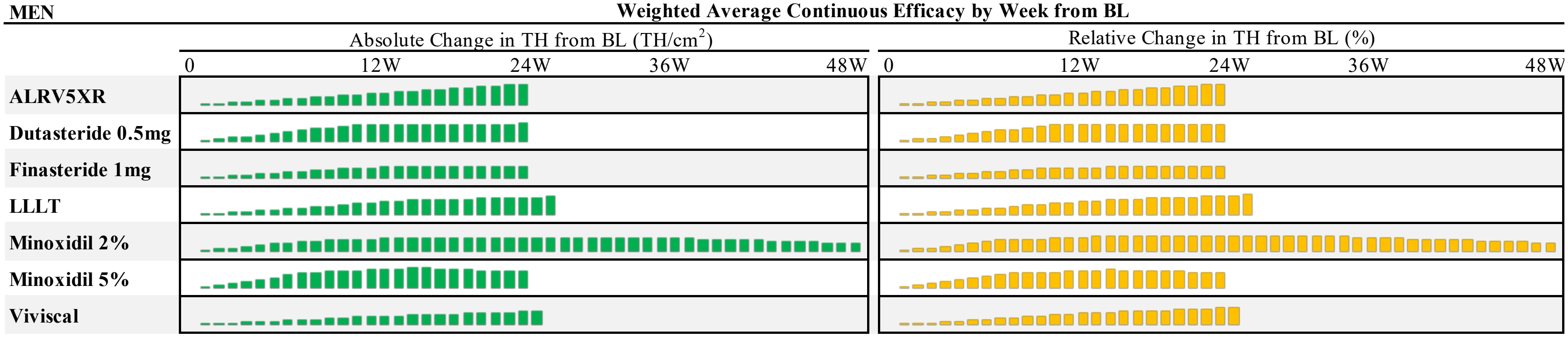

Figure S1-9: Continuous in Men for Treatments with Multiple Studies

Continuous Outcomes in Men for Treatments with Multiple Studies

(A) **LLLT**

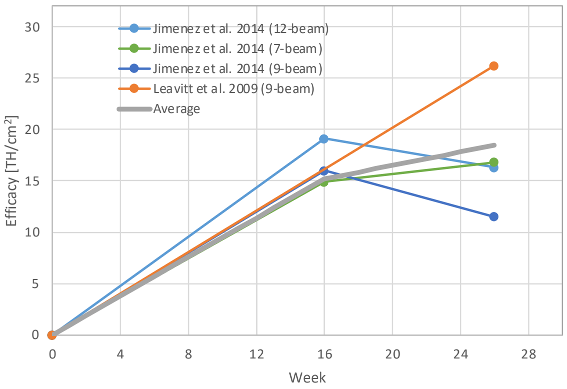

(B) **Minoxidil 5%**

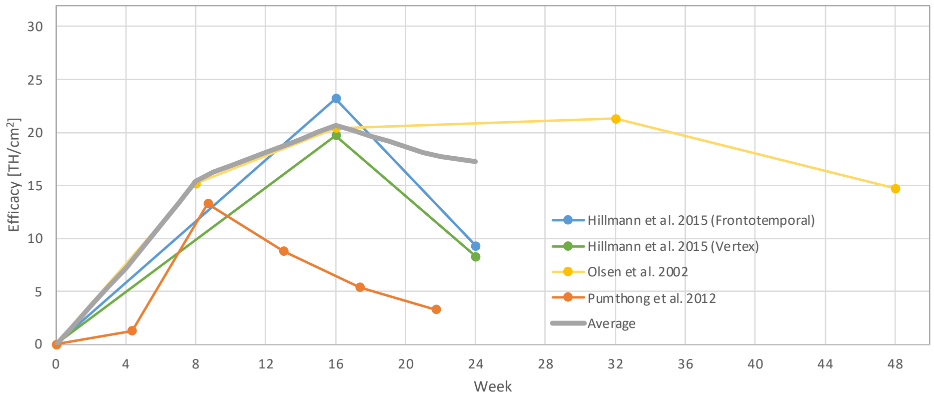

Front. Med. 9:998623. doi: 10.3389/fmed.2022.998623

Section 5: Meta-Analysis Results

Figure S1-10: Meta-Analysis Forest Plot for Women after 24 weeks of treatment

Study	Treatment Total	Treatment Mean	Treatment SD	Placebo Total	Placebo Mean	Placebo SD	MD	95%-CI	Weight
**Treatment = ALRV5XR**									
Feldman 2021	18	33.94	29.37	21	3.86	15.74	30.08	[14.93; 45.23]	2.7%
Random effects model	18			21			30.08	[14.93; 45.23]	2.7%
Heterogeneity: not applicable									
Test for effect in subgroup: $z = 3.89$ ($p < 0.0001$)									
**Treatment = LLLT**									
Jimenez 2014	42	19.12	10.72	21	2.50	16.16	16.62	[8.99; 24.25]	7.3%
Jimenez 2014	39	18.86	11.46	18	2.24	9.00	16.62	[11.12; 22.12]	10.1%
Random effects model	81			39			16.62	[12.16; 21.08]	17.4%
Heterogeneity: $I^2 = 0\%$, $\tau^2 = 0$, $p = 1.00$									
Test for effect in subgroup: $z = 7.30$ ($p < 0.0001$)									
**Treatment = Minoxidil 2%**									
Olsen 1991	14	37.61	29.76	14	15.48	21.30	22.13	[2.95; 41.30]	1.8%
Whiting 1992	14	21.00	31.00	14	17.00	12.00	4.00	[-13.41; 21.41]	2.1%
Jacobs 1993	147	26.10	24.25	135	14.00	27.60	12.10	[6.01; 18.19]	9.2%
De Villez 1994	125	22.20	22.36	125	9.90	19.57	12.30	[7.09; 17.51]	10.6%
Lucky 2004	108	31.30	21.00	51	17.60	18.30	13.70	[7.30; 20.10]	8.8%
Random effects model	408			339			12.60	[9.34; 15.85]	32.5%
Heterogeneity: $I^2 = 0\%$, $\tau^2 = < 0.0001$, $p = 0.73$									
Test for effect in subgroup: $z = 7.58$ ($p < 0.0001$)									
**Treatment = Minoxidil 5%**									
Lucky 2004	101	31.55	22.80	51	17.60	18.30	13.95	[7.24; 20.66]	8.4%
Bergfeld 2016	159	13.40	22.30	165	4.30	16.20	9.10	[4.84; 13.36]	12.2%
Random effects model	260			216			10.80	[6.27; 15.34]	20.6%
Heterogeneity: $I^2 = 30\%$, $\tau^2 = 3.5455$, $p = 0.23$									
Test for effect in subgroup: $z = 4.67$ ($p < 0.0001$)									
**Treatment = Nutrafol**									
Ablon 2018	26	14.03	9.25	14	4.29	6.02	9.74	[4.99; 14.49]	11.3%
Ablon 2021	33	6.45	4.96	27	0.22	4.00	6.23	[3.97; 8.50]	15.5%
Random effects model	59			41			7.34	[4.15; 10.53]	26.8%
Heterogeneity: $I^2 = 41\%$, $\tau^2 = 2.5406$, $p = 0.19$									
Test for effect in subgroup: $z = 4.50$ ($p < 0.0001$)									
**Random effects model**	**826**			**656**			**12.15**	**[9.45; 14.85]**	**100.0%**
Heterogeneity: $I^2 = 65\%$, $\tau^2 = 10.8848$, $p < 0.01$									
Test for overall effect: $z = 8.82$ ($p < 0.0001$)									
Test for subgroup differences: $\chi^2_4 = 17.89$, df = 4 ($p < 0.01$)									

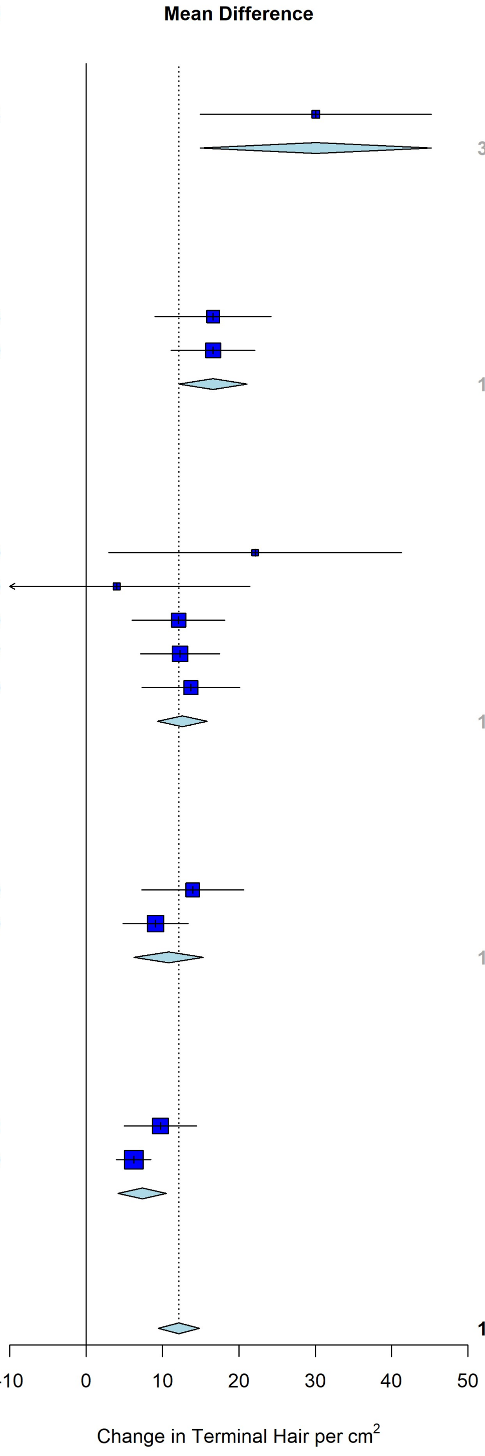

Front. Med. 9:998623. doi: 10.3389/fmed.2022.998623

Figure S1-11: Meta-Analysis Forest Plot for Women after 12 weeks of treatment

Study	Treatment Total	Treatment Mean	Treatment SD	Placebo Total	Placebo Mean	Placebo SD	MD	95%-CI	Weight
**Treatment = ALRV5XR**									
Feldman 2021	18	16.06	16.62	21	3.14	7.49	12.92	[4.60; 21.24]	6.2%
**Random effects model**	**18**			**21**			**12.92**	**[4.60; 21.24]**	**6.2%**
Heterogeneity: not applicable									
Test for effect in subgroup: z = 3.04 (p = 0.0023)									
**Treatment = LLLT**									
Jimenez 2014	43	11.10	7.20	22	0.98	11.10	10.13	[5.01; 15.24]	9.8%
Jimenez 2014	39	8.93	8.48	18	-0.60	5.85	9.53	[5.73; 13.32]	11.6%
**Random effects model**	**82**			**40**			**9.74**	**[6.69; 12.78]**	**21.4%**
Heterogeneity: I^2 = 0%, τ^2 = 0, p = 0.85									
Test for effect in subgroup: z = 6.27 (p < 0.0001)									
**Treatment = Minoxidil 2%**									
Whiting 1992	16	20.50	23.00	16	8.50	15.00	12.00	[-1.45; 25.45]	3.2%
Jacobs 1993	155	35.50	32.68	139	19.80	25.05	15.70	[9.08; 22.32]	7.9%
De Villez 1994	121	25.50	25.63	121	9.20	22.00	16.30	[10.28; 22.32]	8.7%
Lucky 2004	108	27.10	20.35	51	15.55	19.20	11.55	[5.03; 18.07]	8.1%
**Random effects model**	**400**			**327**			**14.42**	**[10.88; 17.97]**	**27.9%**
Heterogeneity: I^2 = 0%, τ^2 = 0, p = 0.71									
Test for effect in subgroup: z = 7.97 (p < 0.0001)									
**Treatment = Minoxidil 5%**									
Lucky 2004	101	28.85	20.65	51	15.55	19.20	13.30	[6.67; 19.93]	7.9%
Bergfeld 2016	172	16.40	21.50	168	5.30	15.30	11.10	[7.14; 15.06]	11.4%
**Random effects model**	**273**			**219**			**11.68**	**[8.28; 15.08]**	**19.3%**
Heterogeneity: I^2 = 0%, τ^2 = 0, p = 0.58									
Test for effect in subgroup: z = 6.73 (p < 0.0001)									
**Treatment = Nutrafol**									
Ablon 2018	26	9.05	8.55	14	0.93	5.37	8.12	[3.79; 12.45]	10.9%
Ablon 2021	33	2.71	3.85	27	-0.28	2.71	2.99	[1.32; 4.65]	14.2%
**Random effects model**	**59**			**41**			**5.15**	**[0.18; 10.12]**	**25.1%**
Heterogeneity: I^2 = 79%, τ^2 = 10.3775, p = 0.03									
Test for effect in subgroup: z = 2.03 (p = 0.0422)									
**Random effects model**	**832**			**648**			**10.51**	**[7.79; 13.23]**	**100.0%**
Heterogeneity: I^2 = 81%, τ^2 = 12.8224, p < 0.01									
Test for overall effect: z = 7.57 (p < 0.0001)									
Test for subgroup differences: χ^2_4 = 9.92, df = 4 (p = 0.04)									

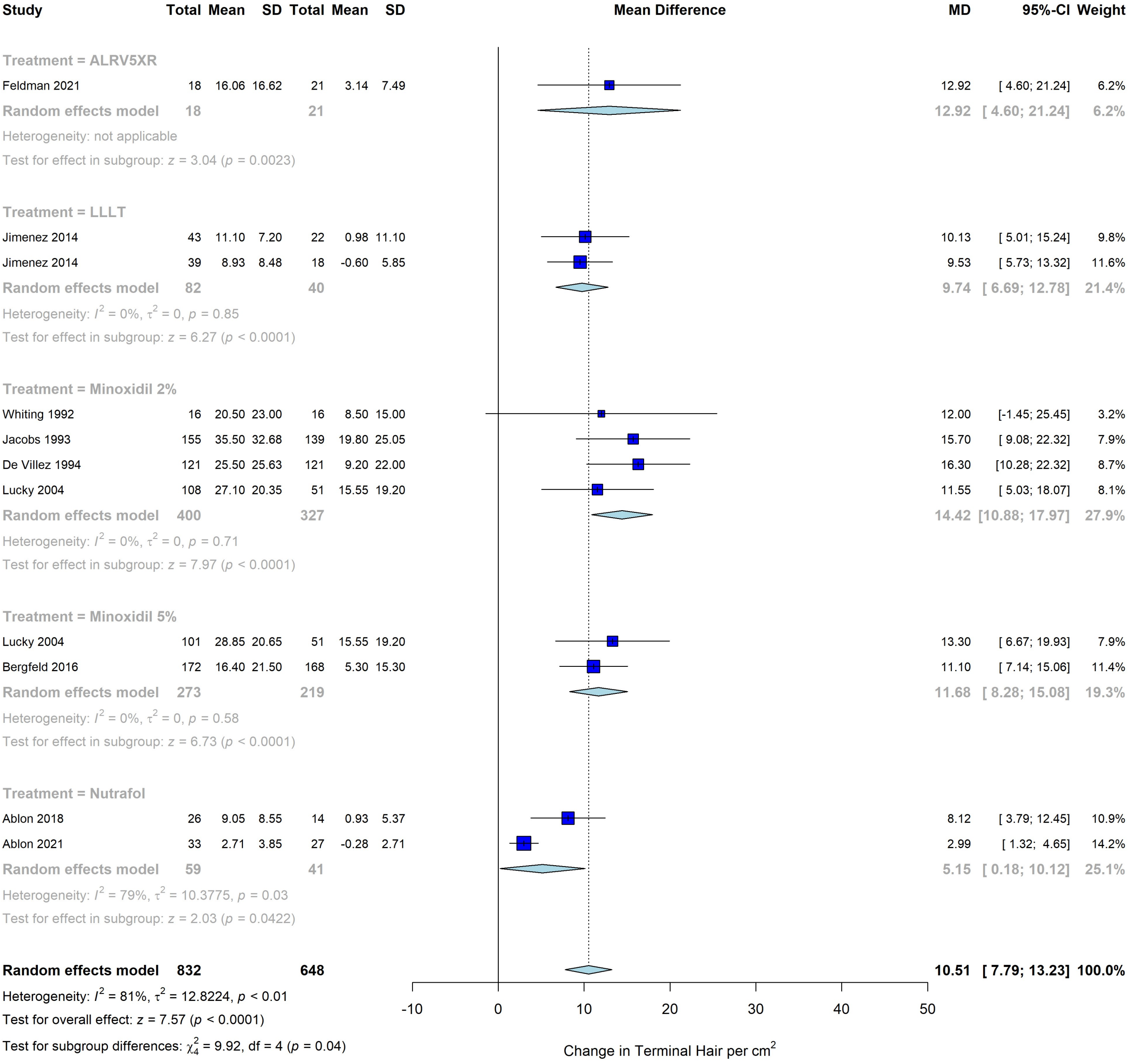

Figure S1-12: Meta-Analysis Forest Plot for Men after 24 weeks of treatment

Study	Treatment Total	Treatment Mean	Treatment SD	Placebo Total	Placebo Mean	Placebo SD	MD	95%-CI	Weight
**Treatment = ALRV5XR**									
Feldman 2021	11	15.18	18.02	11	-5.82	5.46	21.00	[9.87; 32.13]	5.0%
**Random effects model**	**11**			**11**			**21.00**	**[9.87; 32.13]**	**5.0%**
Heterogeneity: not applicable									
Test for effect in subgroup: z = 3.70 (p = 0.0002)									
**Treatment = Dutasteride 0.5mg**									
Gubelin Harcha 2014	152	18.10	21.45	152	-0.30	21.45	18.40	[13.58; 23.22]	12.1%
**Random effects model**	**152**			**152**			**18.40**	**[13.58; 23.22]**	**12.1%**
Heterogeneity: not applicable									
Test for effect in subgroup: z = 7.48 (p < 0.0001)									
**Treatment = Finasteride 1mg**									
Gubelin Harcha 2014	142	12.10	21.45	152	-0.30	21.45	12.40	[7.49; 17.31]	12.0%
**Random effects model**	**142**			**152**			**12.40**	**[7.49; 17.31]**	**12.0%**
Heterogeneity: not applicable									
Test for effect in subgroup: z = 4.95 (p < 0.0001)									
**Treatment = LLLT**									
Leavitt 2009	71	15.97	11.90	39	-8.22	11.70	24.18	[19.59; 28.78]	12.5%
Jimenez 2014	24	18.26	13.30	14	1.84	8.34	16.42	[9.54; 23.30]	9.0%
Jimenez 9+12 beam 2014	40	22.94	15.57	21	8.40	11.92	14.54	[7.52; 21.56]	8.8%
**Random effects model**	**135**			**74**			**18.88**	**[12.70; 25.06]**	**30.3%**
Heterogeneity: I^2 = 69%, τ^2 = 19.9774, p = 0.04									
Test for effect in subgroup: z = 5.99 (p < 0.0001)									
**Treatment = Minoxidil 2%**									
Olsen 2002	141	26.15	19.59	71	11.50	19.59	14.65	[9.06; 20.24]	10.9%
**Random effects model**	**141**			**71**			**14.65**	**[9.06; 20.24]**	**10.9%**
Heterogeneity: not applicable									
Test for effect in subgroup: z = 5.14 (p < 0.0001)									
**Treatment = Minoxidil 5%**									
Olsen 2002	139	32.35	19.59	71	11.50	19.59	20.85	[15.25; 26.45]	10.8%
Pumthong 2012	22	29.37	36.53	22	22.10	23.59	7.27	[-10.90; 25.44]	2.2%
Hillmann 2015	31	7.80	24.10	34	-1.50	15.00	9.30	[-0.57; 19.17]	5.9%
Hillmann 2015	32	8.70	24.00	31	0.40	23.00	8.30	[-3.31; 19.91]	4.7%
**Random effects model**	**224**			**158**			**13.14**	**[5.40; 20.89]**	**23.6%**
Heterogeneity: I^2 = 58%, τ^2 = 33.3953, p = 0.06									
Test for effect in subgroup: z = 3.33 (p = 0.0009)									
**Treatment = Viviscal**									
Ablon 2016	30	8.03	21.77	30	-5.21	15.07	13.24	[3.77; 22.71]	6.2%
**Random effects model**	**30**			**30**			**13.24**	**[3.77; 22.71]**	**6.2%**
Heterogeneity: not applicable									
Test for effect in subgroup: z = 2.74 (p = 0.0062)									
**Random effects model**	**835**			**648**			**16.30**	**[13.39; 19.21]**	**100.0%**

Heterogeneity: I^2 = 52%, τ^2 = 12.1421, p = 0.02
Test for overall effect: z = 10.98 (p < 0.0001)
Test for subgroup differences: χ^2_6 = 5.64, df = 6 (p = 0.46)

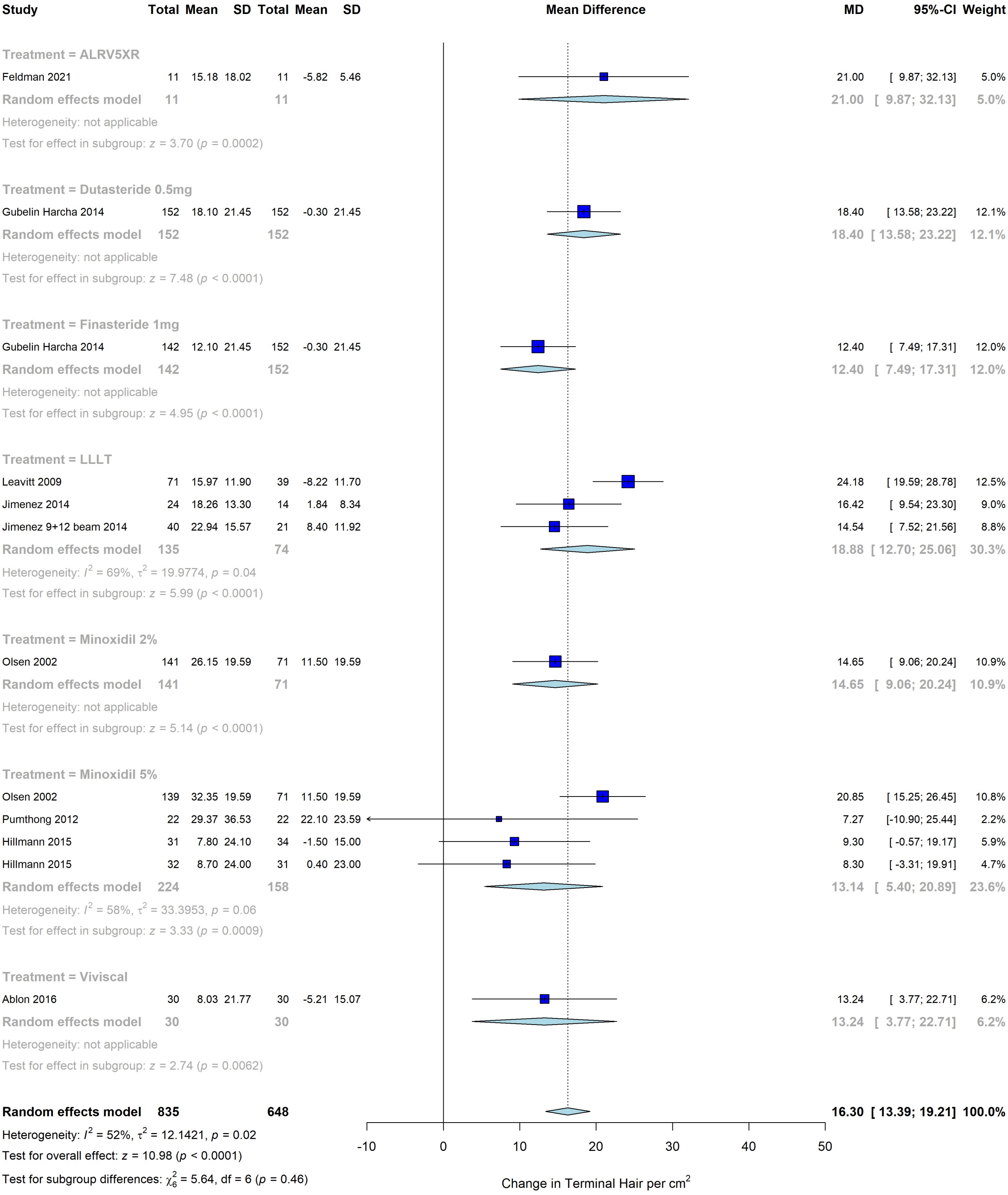

Front. Med. 9:998623. doi: 10.3389/fmed.2022.998623

Figure S1-13: Meta-Analysis Forest Plot for Men after 12 weeks of treatment

Study	Treatment Total	Treatment Mean	Treatment SD	Placebo Total	Placebo Mean	Placebo SD	MD	95%-CI	Weight
**Treatment = ALRV5XR**									
ALRV5XR	11	8.00	8.35	11	-2.95	7.94	10.95	[4.14; 17.76]	7.0%
**Random effects model**	11			11			10.95	[4.14; 17.76]	7.0%
Heterogeneity: not applicable									
Test for effect in subgroup: z = 3.15 (p = 0.0016)									
**Treatment = Dutasteride 0.5mg**									
Dutasteride 0.5mg	149	17.10	20.14	152	-0.40	20.09	17.50	[12.95; 22.05]	13.1%
**Random effects model**	149			152			17.50	[12.95; 22.05]	13.1%
Heterogeneity: not applicable									
Test for effect in subgroup: z = 7.55 (p < 0.0001)									
**Treatment = Finasteride 1mg**									
Finasteride 1mg	134	10.80	20.14	152	-0.40	20.09	11.20	[6.53; 15.87]	12.6%
**Random effects model**	134			152			11.20	[6.53; 15.87]	12.6%
Heterogeneity: not applicable									
Test for effect in subgroup: z = 4.70 (p < 0.0001)									
**Treatment = LLLT**									
LLLT	24	13.27	9.38	14	2.10	5.18	11.17	[6.55; 15.80]	12.8%
LLLT	43	16.49	12.07	22	3.30	6.30	13.19	[8.72; 17.66]	13.4%
**Random effects model**	67			36			12.22	[9.00; 15.43]	26.2%
Heterogeneity: I^2 = 0%, τ^2 = 0, p = 0.54									
Test for effect in subgroup: z = 7.45 (p < 0.0001)									
**Treatment = Minoxidil 2%**									
Minoxidil 2%	141	27.40	18.45	71	14.90	18.45	12.50	[7.24; 17.76]	10.6%
**Random effects model**	141			71			12.50	[7.24; 17.76]	10.6%
Heterogeneity: not applicable									
Test for effect in subgroup: z = 4.66 (p < 0.0001)									
**Treatment = Minoxidil 5%**									
Minoxidil 5%	139	32.70	18.45	71	14.90	18.45	17.80	[12.52; 23.08]	10.5%
Minoxidil 5%	22	23.80	31.09	22	13.95	21.73	9.85	[-6.00; 25.70]	1.5%
Minoxidil 5%	32	20.77	18.60	34	3.38	10.88	17.40	[9.99; 24.81]	6.1%
Minoxidil 5%	32	19.20	18.23	33	4.43	13.27	14.78	[7.00; 22.55]	5.6%
**Random effects model**	225			160			16.61	[12.95; 20.27]	23.7%
Heterogeneity: I^2 = 0%, τ^2 = 0, p = 0.76									
Test for effect in subgroup: z = 8.90 (p < 0.0001)									
**Treatment = Viviscal**									
Viviscal	30	5.32	17.02	30	-2.79	9.19	8.11	[1.19; 15.03]	6.8%
**Random effects model**	30			30			8.11	[1.19; 15.03]	6.8%
Heterogeneity: not applicable									
Test for effect in subgroup: z = 2.30 (p = 0.0216)									
**Random effects model**	**757**			**612**			**13.45**	**[11.49; 15.41]**	**100.0%**

Heterogeneity: I^2 = 15%, τ^2 = 2.2778, p = 0.30
Test for overall effect: z = 13.42 (p < 0.0001)
Test for subgroup differences: χ^2_6 = 10.30, df = 6 (p = 0.11)

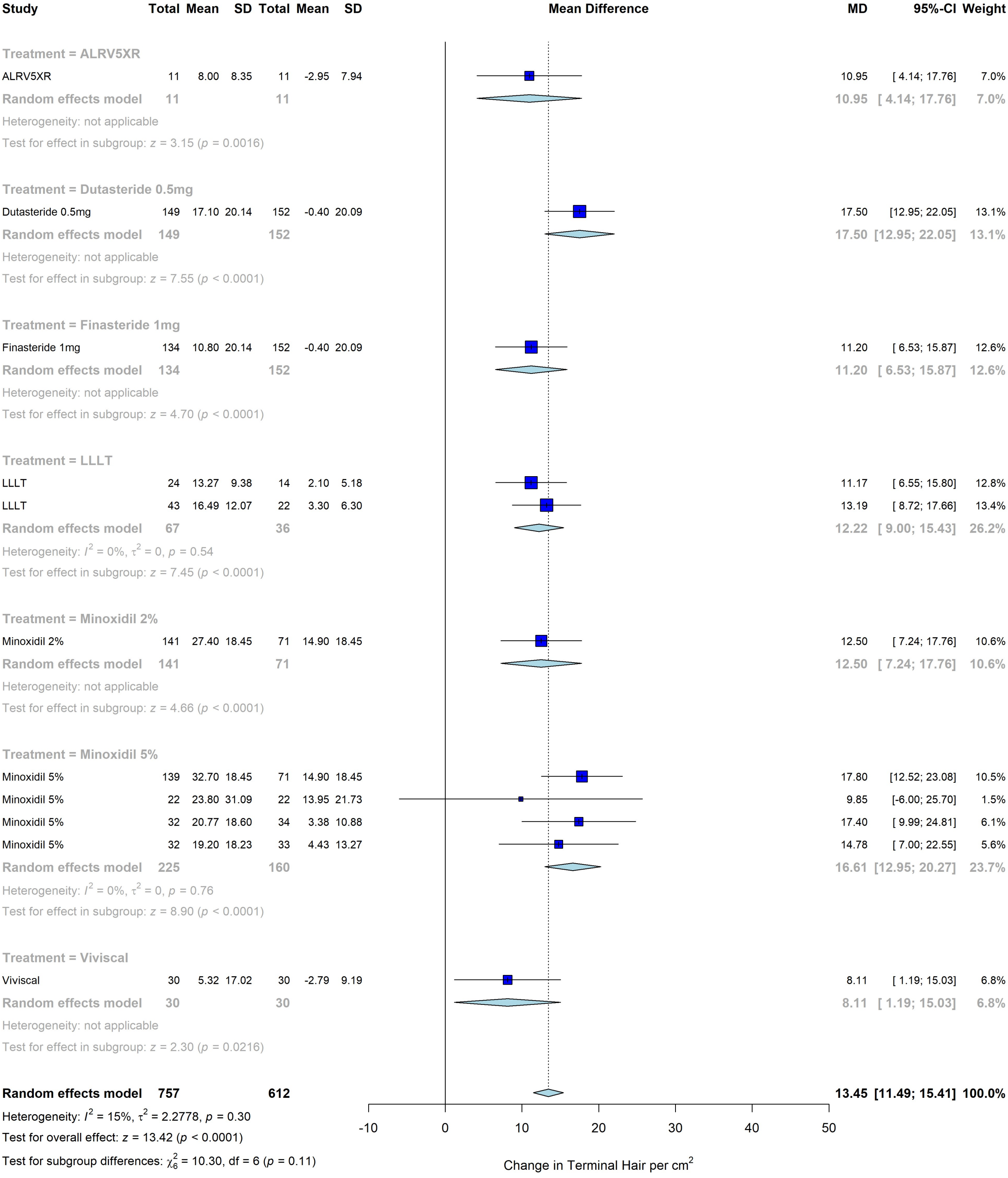

Figure S1-14: Meta-Analysis Funnel Plots

Meta-Analysis Funnel Plots

<u>Women</u>

(A) **24 Weeks**

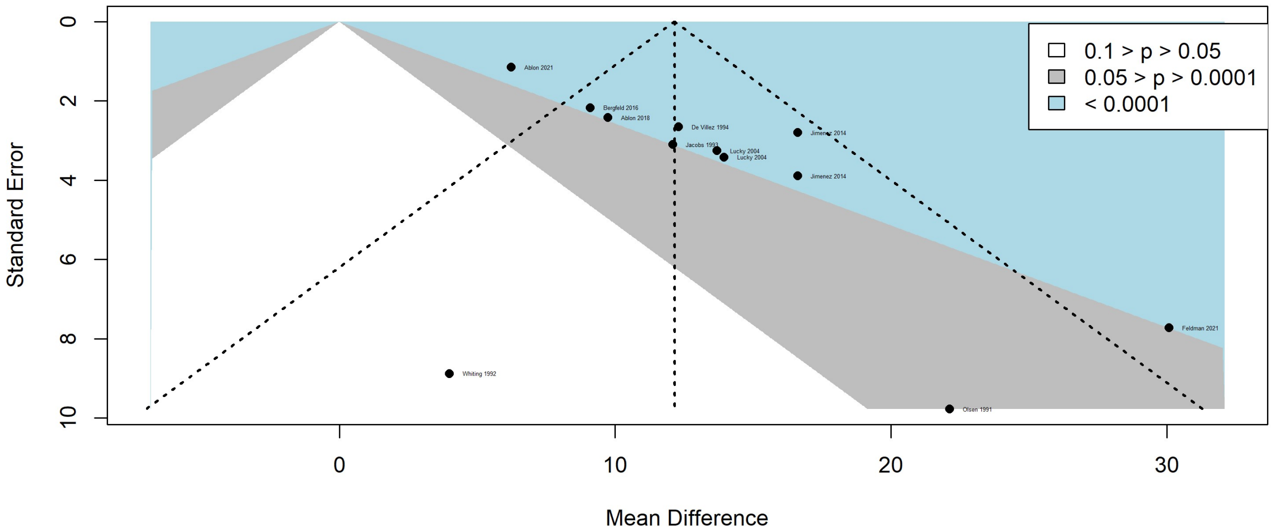

(B) **12 Weeks**

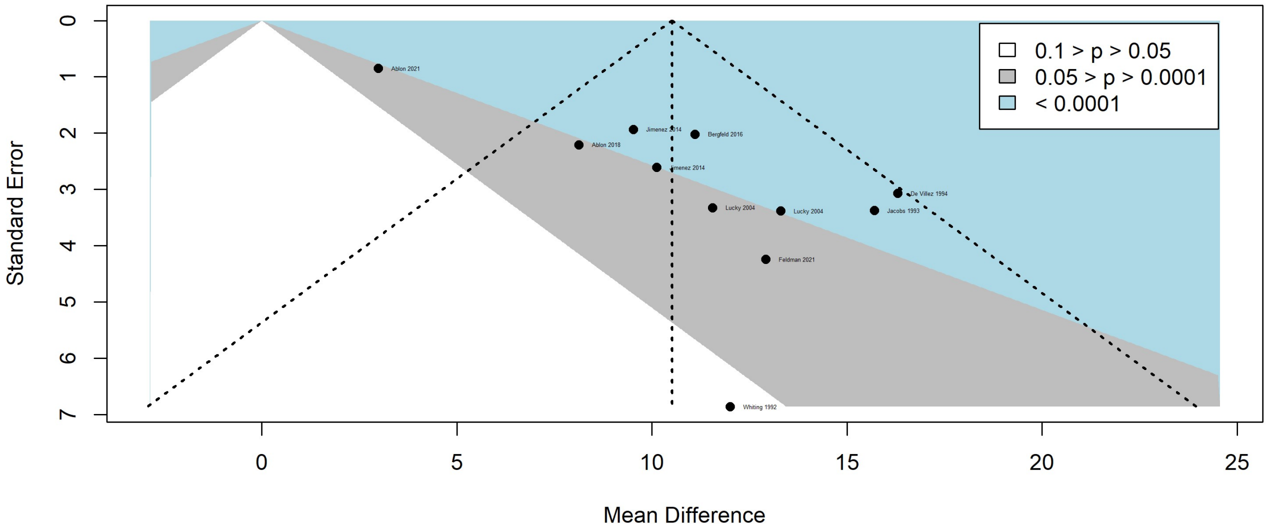

Front. Med. 9:998623. doi: 10.3389/fmed.2022.998623

Figure S1-13: Meta-Analysis Funnel Plots (Continued)

Meta-Analysis Funnel Plots

Men

(C) **24 Weeks**

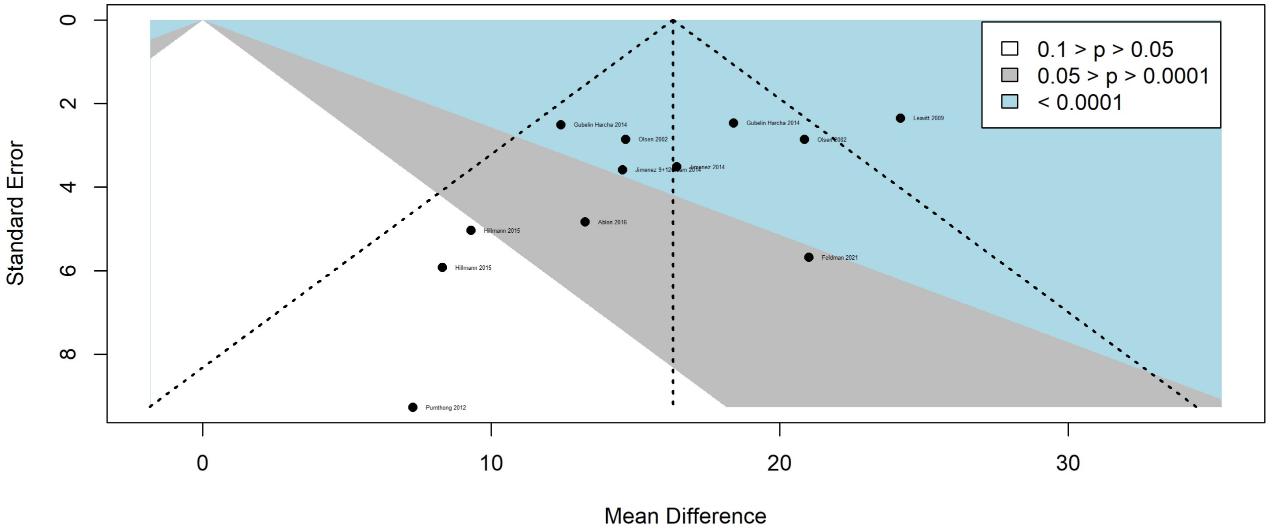

(D) **12 Weeks**

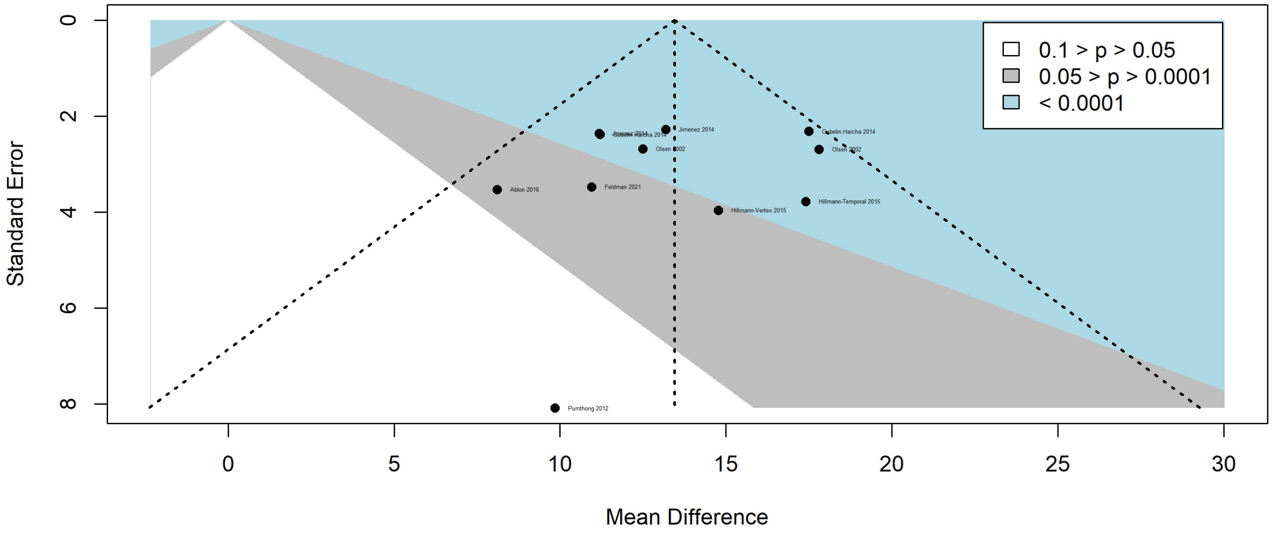

Section 6: Safety Results

Table S1-12: Summary of Adverse Events (AEs) from Eligible Studies.

This table shows presence of Adverse Events by type reported from treatment groups of eligible studies. No analysis has been performed on frequency, severity or comparisons to placebo groups. In women, Minoxidil 5% reported the widest range of AEs followed by Minoxidil 2%. In men, Minoxidil 5% had the widest range of AEs followed by Dutasteride 0.5mg and Finasteride 1mg reporting the same types of AEs. LLLT and natural treatments had few to no AEs.

	Women					Men						
**Adverse Events**	**ALRV5XR**	**LLLT**	**Minoxodil 2%**	**Minoxidil 5%**	**Nutrafol**	**ALRV5XR**	**Dutasteride 0.5mg**	**Finasteride 1mg**	**LLLT**	**Minoxidil 2%**	**Minoxidil 5%**	**Viviscal**
Cardiovascular			Y	Y								
Change in weight				Y			Y	Y				
Elevated Blood Pressure				Y						Y	Y	
Erythema			Y	Y								
Feeling of Skin Tension											Y	
Folliculitis			Y									
Gastrointestinal Complaints				Y	Y		Y	Y				
Hair Loss											Y	
Headache			Y	Y			Y	Y		Y	Y	
Hypertrichosis			Y	Y								
Malaise and Fatigue			Y		Y							
Nasopharyngitis							Y	Y				
Neurology-related Complaints									Y			
Palpitations											Y	
Papules				Y					Y			
Pruritus		Y	Y	Y						Y	Y	
Sexual Dysfunction							Y	Y				
Skin Disorders (dermatitis)		Y	Y	Y						Y	Y	
**Total**	**0**	**2**	**8**	**10**	**2**	**0**	**5**	**5**	**2**	**4**	**7**	**0**

Front. Med. 9:998623. doi: 10.3389/fmed.2022.998623
