# Supplementary material for: Hair regrowth treatment efficacy and resistance in androgenetic alopecia: A systematic review and continuous Bayesian network meta-analysis
Source: Front Med (Lausanne). 2023 Jan 23;9:998623. doi: 10.3389/fmed.2022.998623 (PMC9900126; doi:10.3389/fmed.2022.998623)
Supplement: Supplementary file 2 [file Data_Sheet_2.pdf]

Supplementary Materials

Appendix 2 – Search Strategy, Articles Searched and Reviewed

Hair Regrowth Treatment Efficacy and Resistance in Androgenetic Alopecia: A Systematic Review and Continuous Bayesian Network Meta-Analysis

Peter R. Feldman[1,2*], Pietro Gentile[3], Charles Piwko[4], Hendrik M. Motswaledi[5], Samantha Gorun[6,7], Jacob Pesachov[8], Michael Markel[8], Maxwell I. Silver[9,10], Megan Brenkel[11], Oriel J. Feldman[1,12], Corey L. Kamen[8], Elizabeth Uleryk[13], Jaime Guevara-Aguirre[14,15,16,17], Klaus M. Fiebig[1]

[1] Arbor Life Labs, Toronto, ON, Canada
[2] Norwich Medical School, University of East Anglia, Norwich, United Kingdom
[3] Surgical Science Department, University of Rome Tor Vergata, Rome, Italy
[4] CHP Pharma Inc., Thornhill, ON, Canada
[5] Department of Dermatology, Sefako Makgatho Health Sciences University, South Africa
[6] Faculty of Epidemiology & Biostatistics, Western University, ON, Canada
[7] School or Mathematics and Statistics, University of Glasgow, Glasgow, United Kingdom
[8] Faculty of Medicine, Technion-Israel Institute of Technology, Haifa, Israel
[9] Faculty of Dentistry, University of Toronto, Toronto, ON, Canada
[10] Faculty of Medicine and Dentistry, University of Alberta, Edmonton, AB, Canada
[11] Faculty of Medicine, University of Ottawa, Ottawa, ON, Canada
[12] Faculty of Science, Wilfrid Laurier University, Waterloo, ON, Canada
[13] Uleryk Consulting, Mississauga, ON, Canada
[14] Universidad San Francisco de Quito (USFQ), Quito, Ecuador
[15] Faculty of Health, Medicine and Life Sciences, Maastricht University, Maastricht, Netherlands
[16] Institute of Endocrinology, Metabolism, and Reproduction (IEMYR), Quito, Ecuador
[17] College of Medicine, University of Florida, Gainesville, FL, USA

*** Correspondence:**
Corresponding Author: Peter R Feldman
petefeldman@gmail.com

Keywords: hair loss, ALRV5XR, Dutasteride, Finasteride, LLLT, Minoxidil, Nutrafol, Viviscal

Submission Date: July 20, 2022
Revised: September 12, 2022
Accepted: December 7, 2022
Published: January 23, 2023

CITATION:
Feldman PR, Gentile P, Piwko C, Motswaledi H, Gorun S, Pesachov J, Markel M, Silver MI, Brenkel M, Feldman OJ, Kamen CL, Uleryk E, Guevara-Aguirre J and Fiebig KM (2023) Hair regrowth treatment efficacy and resistance in androgenetic alopecia: A systematic review and Bayesian network meta-analysis. Front. Med. 9:998623. doi: 10.3389/fmed.2022.998623

© 2023 Feldman, Gentile, Piwko, Motswaledi, Gorun, Pesachov, Markel, Silver, Brenkel, Feldman, Kamen, Uleryk, Guevara-Aguirre and Fiebig. This is an open-access article distributed under the terms of the Creative Commons Attribution License (CC BY). The use, distribution or reproduction in other forums is permitted, provided the original author(s) and the copyright owner(s) are credited and that the original publication in this journal is cited, in accordance with accepted academic practice. No use, distribution or reproduction is permitted which does not comply with these terms.

Supplementary Materials

Appendix 2 – Search Strategy, Articles Searched and Reviewed

Hair Regrowth Treatment Efficacy and Resistance in Androgenetic Alopecia: A Systematic Review and Continuous Bayesian Network Meta-Analysis

Table of Contents

**Tables:**	**Page**
Table S2-1. Tabulation of Eligible Studies	3
Table S2-2. Table of Inclusion/Exclusion Reason Codes	3
Table S2-3. Summary of Reasons for Inclusion/Exclusion by Treatment	3
Table S2-4. Eligible Articles Excluded from Study	4
**Article Search Reports**	
Article Database Search Methodology and Strategy Report	12

Front. Med. 9:998623. doi: 10.3389/fmed.2022.998623

Table S2-1. Tabulation of Studies assessed, eligible and treatment cohorts analysed

	Articles Assessed	Eligible Articles			Study Cohorts			Treatments		
		Women	Men	Total	Women	Men	Total	Women	Men	Total
**Sex**	98[a]	10	8	17	12	13	25	5	7	8
Women	48	10	-	10	12	-	12	5	-	5
Men	69	-	8	8	-	13	13	-	7	7

Notes: a: Articles add up to less than total of Men and Women due combined sex enrolments in some studies and common treatments.

Table S2-2. Table of Inclusion/Exclusion Reason Codes

Reason Codes	
1	Eligible
2	Not outcome of interest
3	Experimental formulation/treatment
4	Not randomized placebo-controlled trial
5	Insufficient subjects
6	Not Androgenetic Alopecia or patterned hair loss
7	No extractable data
8	No error data at 24 weeks
9	Data included in subsequent publication
10	Results or crossover < 24 weeks
11	Ineligible hair count methods
12	No sex specific data
13	Not English or German
14	Article not retrievable
15	Total Hair Only
16	Duplicate

Table S2-3. Summary of Primary Reasons for Inclusion/Exclusion by Treatment

	Eligible	Excluded by Reason Code													
**Treatment**	**1**	**2**	**3**	**4**	**5**	**6**	**7**	**9**	**10**	**11**	**12**	**14**	**15**	**16**	**Total**
ALRV5XR	2														**2**
Caffeine												1			**1**
Dutasteride		3													**3**
Dutasteride & Finasteride	1	1													**2**
Electrostatic			1												**1**
Fenugreek		1													**1**
Finasteride		4								2		3			**9**
Ketoconazole		1													**1**
LLLT	2	2					1		3		1				**9**
Minoxidil			1												**1**
Minoxidil 2%	4	2			1					15				1	**23**
Minoxidil 2% & Minoxidil 5%	2														**2**
Minoxidil 5%	3	1						1	2					1	**8**
Niacin		1													**1**
Nourkrin		1													**1**
Nutrafol	2														**2**
Pantogar						1									**1**
PRP		5		1	2		2		3		1	2	1	1	**18**
Serenoa repens		1													**1**
Stem Cell Tx									1		2				**3**
Tocotrienol		1													**1**
Viviscal	1	2				1									**4**
Hairgain		1													**1**
Wnt Activator			2												**2**
**Total**	**17**	**27**	**4**	**1**	**3**	**2**	**3**	**1**	**9**	**17**	**4**	**6**	**1**	**3**	**98**

Front. Med. 9:998623. doi: 10.3389/fmed.2022.998623

Table S2-4. Studies assessed

	Article	Reason Codes			Comments	Treatment
**1**	Ablon G. Double-blind, placebo-controlled study evaluating the efficacy of an oral supplement in women with self-perceived thinning hair. Journal of Clinical and Aesthetic Dermatology. 2012;5(11):28-34.	6			AGA Excluded	Viviscal
**2**	Ablon G. A 6-month, randomized, double-blind, placebo-controlled study evaluating the ability of a marine complex supplement to promote hair growth in men with thinning hair. Journal of Cosmetic Dermatology. 2016;15(4):358-366.	1				Viviscal
**3**	Ablon G, Dayan S. A randomized, double-blind, placebo-controlled, multi-center, extension trial evaluating the efficacy of a new oral supplement in women with self-perceived thinning hair. Journal of Clinical and Aesthetic Dermatology. 2015;8(12):15-21.	2			Outcome in %	Viviscal
**4**	Ablon G, Kogan S. A six-month, randomized, double-blind, placebo-controlled study evaluating the safety and efficacy of a nutraceutical supplement for promoting hair growth in women with self-perceived thinning hair. Journal of Drugs in Dermatology. 2018;17(5):558-565.	1				Nutrafol
**5**	Ablon G, Kogan S. A Randomized, Double-Blind, Placebo-Controlled Study of a Nutraceutical Supplement for Promoting Hair Growth in Perimenopausal, Menopausal, and Postmenopausal Women With Thinning Hair. J Drugs Dermatol. 2021;20(1):55-61.	1				Nutrafol
**6**	Alanis A, Barbara F, Meurehg C, Montes de Oca F, Ramirez L. Double-blind comparison of 2% topical minoxidil and placebo in early male pattern baldness. Current Therapeutic Research - Clinical and Experimental. 1991;49(5):723-730.	11			Manual	Minoxidil 2%
**7**	Alves R, Grimalt R. Randomized placebo-controlled, double-blind, half-head study to assess the efficacy of platelet-rich plasma on the treatment of androgenetic alopecia. Dermatologic Surgery. 2016;42(4):491-497.	12				PRP
**8**	Anderson CD, Hansted B, Abdallah MA, et al. Topical minoxidil in androgenetic alopecia. Scandinavian and Middle East experience. International journal of dermatology. 1988;27(6 SUPPL.):447-451.	11			Manual	Minoxidil 2%
**9**	Barikbin B, Khodamrdi Z, Kholoosi L, et al. Comparison of the effects of 665 nm low level diode Laser Hat versus and a combination of 665 nm and 808nm low level diode Laser Scanner of hair growth in androgenic alopecia. J Cosmet Laser Ther. 2017;17:17.	12				LLLT
**10**	Beoy LA, Woei WJ, Hay YK. Effects of tocotrienol supplementation on hair growth in human volunteers. Trop Life Sci Res. 2010;21(2):91-99.	2	12	15	Patchy hair loss	Tocotrienol
**11**	Bergfeld W, Washenik K, Callender V, et al. A phase III, multicenter, parallel-design clinical trial to compare the efficacy and safety of 5% minoxidil foam versus vehicle in women with female pattern hair loss. Journal of Drugs in Dermatology. 2016;15(7):874-881.	1				Minoxidil 5%

**12**	Blume-Peytavi U, Issiakhem Z, Gautier S, et al. Efficacy and safety of a new 5% minoxidil formulation in male androgenetic alopecia: A randomized, placebo-controlled, double-blind, noninferiority study. Journal of Cosmetic Dermatology. 2019;18(1):215-220.	10			16 Weeks	Minoxidil 5%
**13**	Cervelli V, Garcovich S, Bielli A, et al. The effect of autologous activated platelet rich plasma (AA-PRP) injection on pattern hair loss: Clinical and histomorphometric evaluation. BioMed Research International. 2014;2014 (no pagination)(760709).	5			PRP	PRP
**14**	Civatte J, Degreef H, Dockx P, et al. Topical 2% minoxidil solution in male pattern alopecia: The initial european experience. International Journal of Dermatology. 1988;27(6 SUPPL.):424-429.	11			Manual	Minoxidil 2%
**15**	Civatte J, Laux B, Simpson NB, Vickers CF. 2% topical minoxidil solution in male-pattern baldness: preliminary European results. Dermatologica. 1987;175 Suppl 2:42-49.	11			Manual	Minoxidil 2%
**16**	Connors TJ, Cooke DE, De Launey WE, et al. Australian trial of topical minoxidil and placebo in early male pattern baldness. The Australasian journal of dermatology. 1990;31(1):17-25.	11			Manual	Minoxidil 2%
**17**	De Villez RL. Androgenetic alopecia treated with topical minoxidil. Journal of the American Academy of Dermatology. 1987;16(3 II SUPPL.):669-672.	11			Manual	Minoxidil 2%
**18**	DeVillez RL, Jacobs JP, Szpunar CA, Warner ML. Androgenetic alopecia in the female: Treatment with 2% topical minoxidil solution. Archives of Dermatology. 1994;130(3):303-307.	1				Minoxidil 2%
**19**	Dicle O, Bilgic Temel A, Gulkesen KH. Platelet-rich plasma injections in the treatment of male androgenetic alopecia: A randomized placebo-controlled crossover study. Journal of Cosmetic Dermatology. 2020;19(5):1071-1077.	10			PRP	PRP
**20**	Draelos ZD, Jacobson EL, Kim H, Kim M, Jacobson MK. A pilot study evaluating the efficacy of topically applied niacin derivatives for treatment of female pattern alopecia. Journal of Cosmetic Dermatology. 2005;4(4):258-261.	2			Hair Fullness	Niacin
**21**	Dubin DP, Lin MJ, Leight HM, et al. The effect of platelet-rich plasma on female androgenetic alopecia: A randomized controlled trial. Journal of the American Academy of Dermatology. 2020;83(5):1294-1297.	15			PRP	PRP
**22**	Dutree-Meulenberg R. O. G. M. MD, C. Nieboer, M.D., PH.D., F. H . j ., Koedijk, M.D., and E. Stolz, M.D., PH.D. Treatment of Male Pattern Alopecia Using Topical Minoxidil in The Netherlands. INTERNATIONAL JOURNAL OF DERMATOLOCY. 1988;27(6):435-440.	11			Manual	Minoxidil 2%
**23**	Eun HC, Kwon OS, Yeon JH, et al. Efficacy, safety, and tolerability of dutasteride 0.5 mg once daily in male patients with male pattern hair loss: A randomized, double-blind, placebo-controlled, phase III study. Journal of the American Academy of Dermatology. 2010;63(2):252-258.	2	15			Dutasteride
**24**	Feldman PR, Fiebig KM, Piwko C, et al. Safety and efficacy of ALRV5XR in women with androgenetic alopecia or telogen effluvium: A randomised, double-blinded, placebo-controlled clinical trial. EClinicalMedicine. 2021;37 (no pagination)(100978).	1				ALRV5XR

**25**	Feldman PR, Fiebig KM, Piwko C, et al. Safety and efficacy of ALRV5XR in men with androgenetic alopecia: A randomised, double-blinded, placebo-controlled clinical trial. EClinicalMedicine. 2021;40:101124.	1				ALRV5XR
**26**	Finasteride Male Pattern Hair Loss Study Group. Long-term (5-year) multinational experience with finasteride 1 mg in the treatment of men with androgenetic alopecia. European Journal of Dermatology. 2002;12(1):38-49.	14			Article not retrievable	Finasteride
**27**	Friedman S, Schnoor P. Novel Approach to Treating Androgenetic Alopecia in Females with Photobiomodulation (Low-Level Laser Therapy). Dermatologic Surgery. 2017;43(6):856-867.	10			17 Weeks	LLLT
**28**	Gentile P. The effect of autologous activated platelet rich plasma (AA-PRP) injection on pattern hair loss: clinical and histomorphometric evaluation. Biomed research international. 2014;2014:760709.	16			Same as Cervelli, 2014	PRP
**29**	Gentile P. Autologous cellular method using micrografts of human adipose tissue derived follicle stem cells in androgenic alopecia. International journal of molecular sciences. 2019;20(14).	12				Stem Cell Tx
**30**	Gentile P, Cole JP, Cole MA, et al. Evaluation of not-activated and activated PRP in hair loss treatment: Role of growth factor and cytokine concentrations obtained by different collection systems. International Journal of Molecular Sciences. 2017;18 (2) (no pagination)(408).	2	15			PRP
**31**	Gentile P, Garcovich S, Bielli A, Scioli MG, Orlandi A, Cervelli V. The effect of platelet-rich plasma in hair regrowth: A randomized placebo-controlled trial. Stem Cells Translational Medicine. 2015;4(11):1317-1323.	10				PRP
**32**	Gentile P, Garcovich S, Scioli MG, Bielli A, Orlandi A, Cervelli V. Mechanical and Controlled PRP Injections in Patients Affected by Androgenetic Alopecia. Journal of visualized experiments : JoVE. 2018(pagination).	2	15			PRP
**33**	Ghonemy S, Alarawi A, Bessar H. Efficacy and safety of a new 10% topical minoxidil versus 5% topical minoxidil and placebo in the treatment of male androgenetic alopecia: a trichoscopic evaluation. Journal of Dermatological Treatment. 2021;32(2):236-241.	2			Outcomes Grade, Ratios	Minoxidil 5%
**34**	Ghonemy S, Bessar H, Alarawi A. Efficacy and safety of a new 10% topical minoxidil versus 5% topical minoxidil and placebo in the treatment of male androgenetic alopecia: a trichoscopic evaluation. Journal of dermatological treatment. 2019:1-24.	16			Author Sequence changed	Minoxidil 5%
**35**	Gressenberger P, Pregartner G, Gary T, Wolf P, Kopera D. Platelet-rich Plasma for Androgenetic Alopecia Treatment: A Randomized Placebo-controlled Pilot Study. Acta Derm Venereol. 2020;100(15):adv00247.	2	15			PRP
**36**	Gubelin Harcha W, Barboza Martinez J, Tsai TF, et al. A randomized, active- and placebo-controlled study of the efficacy and safety of different doses of dutasteride versus placebo and finasteride in the treatment of male subjects with androgenetic alopecia. Journal of the American Academy of Dermatology. 2014;70(3):489-498.	1				Dutasteride, Finasteride
**37**	Gupta T. Efficacy of platelet rich plasma in treating androgenetic alopecia. Journal of the Dermatology Nurses' Association Conference: 24th World Congress of Dermatology Milan Italy. 2020;12(2).	2	15			PRP
**38**	Hansted B, Abdallah MA, Oumeish OY, et al. Topical minoxidil in androgenetic alopecia. Scandinavian and Middle East experience. International Journal of Dermatology. 1988;27(6 SUPPL.):447-451.	16			Same as Anderson, 1988	Minoxidil 2%

**39**	Hillmann K, Garcia Bartels N, Kottner J, Stroux A, Canfield D, Blume-Peytavi U. A Single-Centre, Randomized, Double-Blind, Placebo-Controlled Clinical Trial to Investigate the Efficacy and Safety of Minoxidil Topical Foam in Frontotemporal and Vertex Androgenetic Alopecia in Men. Skin Pharmacology and Physiology. 2015;28(5):236-244.	1				Minoxidil 5%
**40**	Hillmann K, Garcia Bartels N, Stroux A, Canfield D, Blume-Peytavi U. Investigator-initiated double-blind, two-armed, placebo-controlled, randomized clinical trial with an open-label extension phase, to investigate the efficacy of 5% Minoxidil topical foam twice daily in men with androgenetic alopecia in the fronto-temporal and vertex regions regarding hair volume over 24/52 weeks. Journal of Investigative Dermatology. 2013;133 (5):1400.	9				Minoxidil 5%
**41**	Jacobs JP, Szpunar CA, Warner ML. Use of topical minoxidil therapy for androgenetic alopecia in women. International Journal of Dermatology. 1993;32(10):758-762.	1				Minoxidil 2%
**42**	Jimenez JJ, Wikramanayake TC, Bergfeld W, et al. Efficacy and safety of a low-level laser device in the treatment of male and female pattern hair loss: A multicenter, randomized, sham device-controlled, double-blind study. American Journal of Clinical Dermatology. 2014;15(2):115-127.	1				LLLT
**43**	Kachhawa D, Vats G, Sonare D, Rao P, Khuraiya S, Kataiya R. A spilt head study of efficacy of placebo versus platelet-rich plasma injections in the treatment of androgenic alopecia. Journal of Cutaneous and Aesthetic Surgery. 2017;10(2):86-89.	14				PRP
**44**	Kang JS, Zheng Z, Choi MJ, Lee SH, Kim DY, Cho SB. The effect of CD34+ cell-containing autologous platelet-rich plasma injection on pattern hair loss: A preliminary study. Journal of the European Academy of Dermatology and Venereology. 2014;28(1):72-79.	4	15			PRP
**45**	Katz HI, Hien NT, Prawer SE, Goldman SJ. Long-term efficacy of topical minoxidil in male pattern baldness. Journal of the American Academy of Dermatology. 1987;16(3 II SUPPL.):711-718.	11			4 and 12 Months	Minoxidil 2%
**46**	Kaufman KD, Olsen EA, Whiting D, et al. Finasteride in the treatment of men with androgenetic alopecia. Finasteride Male Pattern Hair Loss Study Group. Journal of the American Academy of Dermatology. 1998;39(4 Pt 1):578-589.	2	15			Finasteride
**47**	Kawashima M, Hayashi N, Igarashi A, et al. Finasteride in the treatment of Japanese men with male pattern hair loss. European Journal of Dermatology. 2004;14(4):247-254.	14				Finasteride
**48**	Kim H, Choi JW, Kim JY, Shin JW, Lee SJ, Huh CH. Low-level light therapy for androgenetic alopecia: A 24-week, randomized, double-blind, sham device-controlled multicenter trial. Dermatologic Surgery. 2013;39(8):1177-1183.	2	12	15		LLLT
**49**	Koperski JA, Orenberg EK, Wilkinson DI. Topical minoxidil therapy for androgenetic alopecia. A 30-month study. Archives of Dermatology. 1987;123(11):1483- 1487.	11	10	14	4 and 12 Months	Minoxidil 2%

**50**	Lanzafame RJ, Blanche RR, Bodian AB, Chiacchierini RP, Fernandez-Obregon A, Kazmirek ER. The growth of human scalp hair mediated by visible red light laser and LED sources in males. Lasers in Surgery and Medicine. 2013;45(8):487-495.	10			16 Weeks	LLLT
**51**	Lanzafame RJ, Blanche RR, Chiacchierini RP, Kazmirek ER, Sklar JA. The growth of human scalp hair in females using visible red light laser and LED sources. Lasers in Surgery and Medicine. 2014;46(8):601-607.	10			16 Weeks	LLLT
**52**	Leavitt M, Charles G, Heyman E, Michaels D. HairMax LaserComb laser phototherapy device in the treatment of male androgenetic alopecia: A randomized, double-blind, sham device-controlled, multicentre trial. Clin Drug Invest. 2009;29(5):283-292.	1				LLLT
**53**	Lee WS, Chul Eun H, Sang Kwon O, In Ro B, Young Sim W. Effect of dutasteride on male pattern baldness: Phase III clinical trial: Hormones, hair growth and pattern hair loss. Experimental Dermatology. 2010;19 (6):572.	2	15			Dutasteride
**54**	Lengg N, Heidecker B, Seifert B, Trueb RM. Dietary supplement increases anagen hair rate in women with telogen effluvium: Results of a double-blind, placebo-controlled trial. Therapy. 2007;4(1):59-65.	6	15			Pantogar
**55**	Leyden J, Dunlap F, Miller B, et al. Finasteride in the treatment of men with frontal male pattern hair loss. Journal of the American Academy of Dermatology. 1999;40(6 I):930-937.	2	15			Finasteride
**56**	Lopez-Bran E, Robledo A, Aspiolea F, et al. Multicenter comparative study of the efficacy of topical 2% minoxidil (Regaine) versus placebo in the treatment of male baldness. Advances in Therapy. 1990;7(3):159-168.	11				Minoxidil 2%
**57**	Lucky AW, Piacquadio DJ, Ditre CM, et al. A randomized, placebo-controlled trial of 5% and 2% topical minoxidil solutions in the treatment of female pattern hair loss. Journal of the American Academy of Dermatology. 2004;50(4):541-553.	1				Minoxidil 2%, Minoxidil 5%
**58**	Maddin WS, Bell PW, James JH. The biological effects of a pulsed electrostatic field with specific reference to hair. Electrotrichogenesis. International Journal of Dermatology. 1990;29(6):446-450.	3				Electrostatic
**59**	Mapar MA, Shahriari S, Haghighizadeh MH. Efficacy of platelet-rich plasma in the treatment of androgenetic (male-patterned) alopecia: A pilot randomized controlled trial. Journal of Cosmetic and Laser Therapy. 2016;18(8):452-455.	7			Split Head	PRP
**60**	Morganti P, Fabrizi G, James B, Bruno C. Effect of gelatin-cystine and serenoa repens extract on free radicals level and hair growth. Journal of Applied Cosmetology. 1998;16(3):57-64.	2			Outcome in %	Serenoa Repens
**61**	Olsen EA. Topical minoxidil in the treatment of androgenetic alopecia in women. Cutis. 1991;48(3):243-246+248.	1				Minoxidil 2%
**62**	Olsen EA, DeLong ER, Weiner MS. Dose-response study of topical minoxidil in male pattern baldness. Journal of the American Academy of Dermatology. 1986;15(1):30-37.	11				Minoxidil 2%
**63**	Olsen EA, Dunlap FE, Funicella T, et al. A randomized clinical trial of 5% topical minoxidil versus 2% topical minoxidil and placebo in the treatment of androgenetic alopecia in men. Journal of the American Academy of Dermatology. 2002;47(3):377-385.	1				Minoxidil 2%, Minoxidil 5%

**64**	Olsen EA, Hordinsky M, Whiting D, et al. The importance of dual 5alpha-reductase inhibition in the treatment of male pattern hair loss: results of a randomized placebo-controlled study of dutasteride versus finasteride. Journal of the American Academy of Dermatology. 2006;55(6):1014-1023.	2	15			Dutasteride, Finasteride
**65**	Olsen EA, Weiner MS, Delong ER, Pinnell SR. Topical minoxidil in early male pattern baldness. Journal of the American Academy of Dermatology. 1985;13(2 I):185-192.	2	15			Minoxidil 2%
**66**	Olsen EA, Whiting D, Bergfeld W, et al. A multicenter, randomized, placebo-controlled, double-blind clinical trial of a novel formulation of 5% minoxidil topical foam versus placebo in the treatment of androgenetic alopecia in men. Journal of the American Academy of Dermatology. 2007;57(5):767-774.	10			16 Weeks	Minoxidil 5%
**67**	Petzoldt D, Borelli S, Braun-Falco O, et al. The German double-blind placebo-controlled evaluation of topical minoxidil solution in the treatment of early male pattern baldness. International Journal of Dermatology. 1988;27(6 SUPPL.):430-434.	11				Minoxidil 2%
**68**	Pierard-Franchimont C, De Doncker P, Cauwenbergh G, Pierard GE. Ketoconazole shampoo: effect of long-term use in androgenic alopecia. Dermatology. 1998;196(4):474-477.	2				Ketoconazole
**69**	Prasad HKNR. A randomized double blind study of the effect of finasteride on hair growth in male patients of androgenetic alopecia. Indian journal of dermatology. 2005;50(3):139-145.	11				Finasteride
**70**	Price VH, Menefee E. Quantitative estimation of hair growth I. Androgenetic alopecia in women: Effect of minoxidil. Journal of Investigative Dermatology. 1990;95(6):693-687.	2				Minoxidil 2%
**71**	Price VH, Roberts JL, Hordinsky M, et al. Lack of efficacy of finasteride in postmenopausal women with androgenetic alopecia. Journal of the American Academy of Dermatology. 2000;43(5):768-776.	2	15			Finasteride
**72**	Puig CJ, Reese R, Peters M. Double-blind, placebo-controlled pilot study on the use of platelet-rich plasma in women with female androgenetic alopecia. Dermatologic Surgery. 2016;42(11):1243-1247.	7				PRP
**73**	Pumthong G, Asawanonda P, Varothai S, et al. Curcuma aeruginosa, a novel botanically derived 5alpha-reductase inhibitor in the treatment of male-pattern baldness: a multicenter, randomized, double-blind, placebo-controlled study. Journal of Dermatological Treatment. 2012;23(5):385-392.	1			Curcumin not of interest	Minoxidil 5%
**74**	Rietschel RL, Duncan SH. Safety and efficacy of topical minoxidil in the management of androgenetic alopecia. Journal of the American Academy of Dermatology. 1987;16(3 II SUPPL.):677-685.	11				Minoxidil 2%
**75**	Rizer RL, Stephens TJ, Herndon JH, Sperber BR, Murphy J, Ablon GR. A Marine Protein-based Dietary Supplement for Subclinical Hair Thinning/Loss: Results of a Multisite, Double-blind, Placebo-controlled Clinical Trial. Int J Trichology. 2015;7(4):156-166.	2	6		AGA Excluded	Viviscal
**76**	Roberts JL. Androgenetic alopecia: Treatment results with topical minoxidil. Journal of the American Academy of Dermatology. 1987;16(3 II SUPPL.):705-710.	11				Minoxidil 2%
**77**	Rushton DH, Unger WP, Cotterill PC, Kingsley P, James KC. Quantitative assessment of 2% topical minoxidil in the treatment of male pattern baldness. Clinical and Experimental Dermatology. 1989;14(1):40-46.	5				Minoxidil 2%

**78**	Sasaki GH. The Effects of Lower Versus Higher Cell Number of Platelet-Rich Plasma (PRP) in Hair Density and Diameter in Androgenetic Alopecia (AGA): A Randomized, Double-Blinded, Placebo, Paralleled Group Half-Scalp IRB Study. Aesthet. 2021;29:29.	5			Split Head	PRP
**79**	Schulz C, Bielfeldt S, Reimann J. Fenugreek + micronutrients: Efficacy of a food supplement against hair loss. Kosmetische Medizin. 2006;27(4):176-179.	2	12			Fenugreek
**80**	Shapiro J, Ho A, Sukhdeo K, Yin L, Lo Sicco K. Evaluation of platelet-rich plasma as a treatment for androgenetic alopecia: A randomized controlled trial. Journal of the American Academy of Dermatology. 2020;83(5):1298-1303.	10	15		Split Head	PRP
**81**	Shupack JL, Kassimir JJ, Thirumoorthy T, Reed ML, Jondreau L. Dose-response study of topical minoxidil in male pattern alopecia. Journal of the American Academy of Dermatology. 1987;16(3 Pt 2):673-676.	11				Minoxidil 2%
**82**	Sisto T, Bussoletti C, Celleno L. Efficacy of a cosmetic caffeine shampoo in androgenetic alopecia management. II Note. Journal of Applied Cosmetology. 2013;31(1):57-66.	14				Caffeine
**83**	Stough D. Dutasteride improves male pattern hair loss in a randomized study in identical twins. Journal of Cosmetic Dermatology. 2007;6(1):9-13.	2	15			Dutasteride
**84**	Stough DB, Rao NA, Kaufman KD, Mitchell C. Finasteride improves male pattern hair loss in a randomized study in identical twins. European Journal of Dermatology. 2002;12(1):32-37.	14				Finasteride
**85**	Suchonwanit P, Chalermroj N, Khunkhet S. Low-level laser therapy for the treatment of androgenetic alopecia in Thai men and women: a 24-week, randomized, double-blind, sham device-controlled trial. Lasers in Medical Science. 2019;34(6):1107-1114.	2	15			LLLT
**86**	Tak YJ, Lee SY, Cho AR, Kim YS. A randomized, double-blind, vehicle-controlled clinical study of hair regeneration using adipose-derived stem cell constituent extract in androgenetic alopecia. Stem Cells Translational Medicine. 2020.	10	12			Stem Cell Tx
**87**	Tan CH, Lee JSS, Tan KT, Wang ECE, Chan RKW, Chuah SY. A randomized double-blind, split-scalp, placebocontrolled study to evaluate the efficacy of platelet-rich plasma for the treatment of androgenetic alopecia. British Journal of Dermatology. 2019;181 (Supplement 1):51.	14				PRP
**88**	Tawfik AA, Osman MAR. The effect of autologous activated platelet-rich plasma injection on female pattern hair loss: A randomized placebo-controlled study. Journal of Cosmetic Dermatology. 2018;17(1):47-53.	2	15			PRP
**89**	Thom E. Efficacy and tolerability of hairgain in individuals with hair loss: A placebo-controlled, double-blind study. Journal of International Medical Research. 2001;29(1):2-6.	2	12			Viviscal/Hairga in
**90**	Thom E. Nourkrin: Objective and subjective effects and tolerability in persons with hair loss. Journal of International Medical Research. 2006;34(5):514-519.	2	12			Nourkrin

**91**	Tsuboi R, Niiyama S, Irisawa R, Harada K, Nakazawa Y, Kishimoto J. Autologous cell-based therapy for male and female pattern hair loss using dermal sheath cup cells: A randomized placebo-controlled double-blinded dose-finding clinical study. Journal of the American Academy of Dermatology. 2020.	12	15			Stem Cell Tx
**92**	Uzel BPC, Takano GHS, Chartuni JCN, et al. Intradermal injections with 0.5% minoxidil for the treatment of female androgenetic alopecia: A randomized, placebo-controlled trial. Dermatol Ther. 2021;34(1):e14622.	3				Minoxidil
**93**	Van Neste D, Fuh V, Sanchez-Pedreno P, et al. Finasteride increases anagen hair in men with androgenetic alopecia. British Journal of Dermatology. 2000;143(4):804-810.	2				Finasteride
**94**	Whiting DA, Jacobson C. Treatment of female androgenetic alopecia with minoxidil 2%. International Journal of Dermatology. 1992;31(11):800-804.	1				Minoxidil 2%
**95**	Whiting DA, Waldstreicher J, Sanchez M, Kaufman KD. Measuring reversal of hair miniaturization in androgenetic alopecia by follicular counts in horizontal sections of serial scalp biopsies: Results of finasteride 1 mg treatment of men and postmenopausal women. Journal of Investigative Dermatology Symposium Proceedings. 1999;4(3):282-284.	11			Biopsy	Finasteride
**96**	Yazici Y, Smith S, Swearingen C, Simsek I, Di Francesco A, Hood J. Safety and efficacy of a topical treatment (SM04554) for androgenetic alopecia (AGA): Results from a phase 1 Trial. Journal of the American Academy of Dermatology. 2016;1):AB138.	3				Wnt Activator
**97**	Zanardo L. Effects of a Low Level Laser Therapy on Hormonal, Age-Related Hair Loss in Women after Treatment of 6 Months. [German]. Aktuelle Dermatologie. 2016;42(3):90-95.	7				LLLT
**98**	Zimber MP, Ziering C, Zeigler F, et al. Hair regrowth following a Wnt- and follistatin containing treatment: safety and efficacy in a first-in-man phase 1 clinical trial. J Drugs Dermatol. 2011;10(11):1308-1312.	3				Wnt Activator

Article Database Search Methodology and Strategy Report

Search Methodology:

Alopecia standard of care treatments safety and efficacy - RCTs

Methods section (suggested text for journal article)
A professional librarian (EU) ran a search in the MEDLINE, Medline-in-Process, Medline ePub Ahead of Print and EMBASE databases(OvidSP); and Cochrane (Wiley) on August 11, 2021. We used both subject headings and textword terms to search for articles on Alopecia AND current standard of care replacement therapies (e.g.minoxidil or finasteride or duasteride or biotin or low level laser light therapy or supplements or or transplant, etc.) AND Randomized Clinical Trials. The search results were not limited to publication date or language.

We retrieved a total of **3153** references. All references were saved in an EndNote library used to identify the **843** duplicates. The remaining **2310 + 3** hand searches **+ 1** grey search unique references were reviewed against our inclusion criteria. The complete search strategies are listed in below.

**Database**	**Dates**	**Total**	**Unique**	**Duplicates**
Cochrane	Issue 8 of 12, August 2021	829	422	407
Embase	1980 to 2021 Week 31	957	847	110
HandSearches	Scholarly research	3	3	0
Grey Literature Search	On file	1	1	0
MEDLINE(R)	1946 to July Week 5 2021	1219	953	266
Medline Epub Ahead of Print	to August 10, 2021	18	16	2
Medline -in-Process	1946 to August 10, 2021	130	72	58
**Total**		**3157**	**2314**	**843**

Search Strategy:

#	Searches	Results	Type
1	Alopecia/	11636	
2	((androgenetica or androgenetic or androgenic or male or female) adj2 Alopecia*).ti,ab,kf.	1869	
3	(telogen adj2 effluvium).ti,ab,kf.	261	
4	(("Non scarring" or "non-scarring") adj2 alopecia*).ti,ab,kf.	103	
5	(hair adj2 (loss or thinning or plug or plugs)).ti,ab,kf.	6481	
6	(Baldness or Balding).ti,ab,kf.	1288	
7	("self-reported" adj2 thinning).ti,ab,kf.	0	
8	Hair Follicle/in [Injuries]	38	
9	hair follicle/ and (injury or injuries).ti,ab,kf.	213	
10	or/1-9	16524	Alopecia terms
11	minoxidil/ or (minoxidil or alocutan or alopexy or alopexyl or alostil or aloxidil or "apo-gain" or crecisan or growell or "hair-treat" or hairgaine or hairgrow or headway or hebald or kapodin or kenacin or lacovin or locemix or "locion epc" or loniten or lonnoten or lonolox or lonoten or manoxidil or rogaine or minocutan or minodyl or minona or minorga or minotricon or minovital or "minoxi 5" or minoxicutan or minoxidil or minoximen or minoxitrim or minoxyl or moxidil or multigain or neocapil or neoxidil or nuhair or prexidil or regaine or regroe or regrou or regrowth or rehair or theroxidil or tiazolin or trefostil or unipexil or ylox).mp. or 38304-91-5.rn.	12123	
12	finasteride/ or (finasteride or alocare or andozac or "chibro proscar" or "chibro-proscar" or finastar or finastid or fincar or finired or finpro or fistrin or folians or frosst or genaprost or harifin or "l 652931" or l652931 or "mk 0906" or "mk 906" or mk0906 or mk906 or nasterol or pilus or "pro-cure" or prohair or propecia or propeshia or proscar or prosh or prostacare or prostacom or prostide or prostop or reprostom or symasteride or tensen or uromedin or "ym 152" or m152).mp. or 98319-26-7.rn.	6617	
13	Dutasteride/ or (dutaseride or advodart or avidart or avodart or avolve or duagen or "gg 745" or gg745 or "gi 198745" or "gi 198745x" or gi198745 or gi198745x).mp. or 164656-23-9.rn.	665	
14	Low-Level Light Therapy/ or (low adj2 (level or light or intensity or energy or laser or power) adj2 (therap* or treat*)).ti,ab,kf.	8107	
15	Viviscal.mp.	3	
16	exp Dietary Supplements/ or (nutraceutical* or supplement*).mp.	342827	
17	Nutrafol.mp.	2	
18	biotin/ or (biotin or "bio h tin" or bioepiderm or "bios ii" or biotine or "coenzyme r" or qizenday or "vitamin bw" or "vitamin h").mp. or 58-85-5.rn.	31986	
19	biocytin/ or (biocytin or biotinyllysine or "n biotinyl l lysine").mp. or 576-19-2.rn.	2294	
20	"2 iminobiotin".mp.	61	
21	("2 iminobiotin" or iminobiotin or guanidinobiotin).mp. or 13395-35-2.rn.	105	
22	exp Collagen/ and (administration, oral/ or administration, buccal/ or administration, sublingual/ or administration, topical/ or administration, cutaneous/)	1254	
23	seafood/ or fish products/ or exp shellfish/	16158	
24	((Marine or fish) adj2 extract).mp.	293	
25	exp sharks/	5606	
26	(samumed or SM04554).mp.	0	
27	Replicel.mp.	0	
28	Electric Stimulation Therapy/ or Electric Stimulation/	134849	

29	(Haircell or electrostimulation).mp.	3098	
30	(follica or (hair adj2 follicle adj2 Neogenesis) or "FOL-004" or wounding).mp.	7682	
31	platelet-rich plasma/ or platelet-rich fibrin/ or (platelet adj2 rich adj2 plasma*).ti,ab,kf. or ("platelet-rich" adj2 plasma*).ti,ab,kf.	10503	
32	hair/tr or hair follicle/tr or (hair adj2 (autograft* or graft* or transplant*)).ti,ab,kf.	1379	
33	or/11-32	577691	Hair loss medications, supplements, transplants
34	10 and 33	2735	Base clinical set
35	controlled clinical trial.pt.	94293	
36	randomized controlled trial.pt.	538640	
37	randomized.ab.	456987	
38	placebo.ab.	199567	
39	drug therapy.fs.	2354230	
40	randomly.ab.	307217	
41	trial.ab.	483405	
42	groups.ab.	1897753	
43	or/35-42	4634227	
44	exp animals/ not humans.sh.	4867787	
45	43 not 44	3972091	Cochrane Therapy filter box 3.c
46	34 and 45	1209	Cochrane therapy filter results
47	limit 34 to multicenter study	60	Multicenter studies
48	46 or 47	1219	Final results

Medline Epub Ahead of Print

#	Searches	Results	Comment
1	((androgenetica or androgenetic or androgenic or male or female) adj2 Alopecia*).ti,ab,kf.	77	
2	(telogen adj2 effluvium).ti,ab,kf.	12	
3	(("Non scarring" or "non-scarring") adj2 alopecia*).ti,ab,kf.	3	
4	(hair adj2 (loss or thinning or plug or plugs)).ti,ab,kf.	168	
5	(Baldness or Balding).ti,ab,kf.	24	
6	("self-reported" adj2 thinning).ti,ab,kf.	0	
7	(hair adj2 follicle adj2 (injury or injuries)).ti,ab,kf.	1	
8	or/1-7	233	Alopecia terms
9	(minoxidil or alocutan or alopexy or alopexyl or alostil or aloxidil or "apo-gain" or crecisan or growell or "hair-treat" or hairgaine or hairgrow or headway or hebald or kapodin or kenacin or lacovin or locemix or "locion epc" or loniten or lonnoten or lonolox or lonoten or manoxidil or rogaine or minocutan or minodyl or minona or minorga or minotricon or minovital or "minoxi 5" or minoxicutan or minoxidil or minoximen or minoxitrim or minoxyl or moxidil or multigain or neocapil or neoxidil or nuhair or prexidil or regaine or regroe or regrou or regrowth or rehair or theroxidil or tiazolin or trefostil or unipexil or ylox).mp.	199	
10	(finasteride or alocare or andozac or "chibro proscar" or "chibro-proscar" or finastar or finastid or fincar or finired or finpro or fistrin or folians or frosst or genaprost or harifin or "l 652931" or l652931 or "mk 0906" or "mk 906"	58	

	or mk0906 or mk906 or nasterol or pilus or "pro-cure" or prohair or propecia or propeshia or proscar or prosh or prostacare or prostacom or prostide or prostop or reprostom or symasteride or tensen or uromedin or "ym 152" or m152).mp.		
11	(dutaseride or advodart or avidart or avodart or avolve or duagen or "gg 745" or gg745 or "gi 198745" or "gi 198745x" or gi198745 or gi198745x).mp.	3	
12	(low adj2 (level or light or intensity or energy or laser or power) adj2 (therap* or treat*)).ti,ab,kf.	115	
13	Viviscal.mp.	0	
14	(nutraceutical* or supplement*).mp.	7367	
15	Nutrafol.mp.	0	
16	(biotin or "bio h tin" or bioepiderm or "bios ii" or biotine or "coenzyme r" or qizenday or "vitamin bw" or "vitamin h").mp.	191	
17	(biocytin or biotinyllysine or "n biotinyl l lysine").mp. or 576-19-2.rn.	6	
18	("2 iminobiotin" or iminobiotin or guanidinobiotin).mp. or 13395-35-2.rn.	1	
19	(Collagen adj2 (oral or orally or topical* or transdermal* or cutaneous*)).ti,ab,kf.	9	
20	(seafood or fish or shellfish).ti,ab,kf.	2246	
21	((Marine or fish) adj2 extract).mp.	3	
22	(shark or sharks).ti,ab,kf.	86	
23	(samumed or SM04554).mp.	0	
24	Replicel.mp.	0	
25	(Electric adj2 Stimulation).ti,ab,kf.	63	
26	(Haircell or electrostimulation).mp.	25	
27	(follica or (hair adj2 follicle adj2 Neogenesis) or "FOL-004" or wounding).mp.	102	
28	((platelet adj2 rich adj2 (plasma* or fibrin*)) or ("platelet-rich" adj2 (fibrin or plasma*))).ti,ab,kf.	300	
29	(hair adj2 (autograft* or graft* or transplant*)).ti,ab,kf.	40	
30	or/9-29	10569	Hair loss medications, supplements, transplants
31	8 and 30	67	Base clinical set
32	((randomized or controlled or multicentre or multicenter) adj (trial or trials)).ti,ab,kf.	8734	
33	randomized.ab.	11247	
34	placebo.ab.	3012	
35	randomly.ab.	5447	
36	trial.ab.	11797	
37	groups.ab.	38625	
38	or/32-37	55599	Therapy filter
39	31 and 38	18	Therapy filter results

Medline-in-Process & In-Data-Review Citations

#	Searches	Results	Comment
1	((androgenetica or androgenetic or androgenic or male or female) adj2 Alopecia*).ti,ab,kf.	53	
2	(telogen adj2 effluvium).ti,ab,kf.	10	
3	(("Non scarring" or "non-scarring") adj2 alopecia*).ti,ab,kf.	9	
4	(hair adj2 (loss or thinning or plug or plugs)).ti,ab,kf.	173	
5	(Baldness or Balding).ti,ab,kf.	10	

6	("self-reported" adj2 thinning).ti,ab,kf.	0	
7	(hair adj2 follicle adj2 (injury or injuries)).ti,ab,kf.	0	
8	or/1-7	227	Alopecia terms
9	(minoxidil or alocutan or alopexy or alopexyl or alostil or aloxidil or "apo-gain" or crecisan or growell or "hair-treat" or hairgaine or hairgrow or headway or hebald or kapodin or kenacin or lacovin or locemix or "locion epc" or loniten or lonnoten or lonolox or lonoten or manoxidil or rogaine or minocutan or minodyl or minona or minorga or minotricon or minovital or "minoxi 5" or minoxicutan or minoxidil or minoximen or minoxitrim or minoxyl or moxidil or multigain or neocapil or neoxidil or nuhair or prexidil or regaine or regroe or regrou or regrowth or rehair or theroxidil or tiazolin or trefostil or unipexil or ylox).mp.	204	
10	(finasteride or alocare or andozac or "chibro proscar" or "chibro-proscar" or finastar or finastid or fincar or finired or finpro or fistrin or folians or frosst or genaprost or harifin or "l 652931" or l652931 or "mk 0906" or "mk 906" or mk0906 or mk906 or nasterol or pilus or "pro-cure" or prohair or propecia or propeshia or proscar or prosh or prostacare or prostacom or prostide or prostop or reprostom or symasteride or tensen or uromedin or "ym 152" or m152).mp.	101	
11	(dutaseride or advodart or avidart or avodart or avolve or duagen or "gg 745" or gg745 or "gi 198745" or "gi 198745x" or gi198745 or gi198745x).mp.	0	
12	(low adj2 (level or light or intensity or energy or laser or power) adj2 (therap* or treat*)).ti,ab,kf.	97	
13	Viviscal.mp.	0	
14	(nutraceutical* or supplement*).mp.	7015	
15	Nutrafol.mp.	0	
16	(biotin or "bio h tin" or bioepiderm or "bios ii" or biotine or "coenzyme r" or qizenday or "vitamin bw" or "vitamin h").mp.	254	
17	(biocytin or biotinyllysine or "n biotinyl l lysine").mp. or 576-19-2.rn.	1	
18	("2 iminobiotin" or iminobiotin or guanidinobiotin).mp. or 13395-35-2.rn.	1	
19	(Collagen adj2 (oral or orally or topical* or transdermal* or cutaneous*)).ti,ab,kf.	4	
20	(seafood or fish or shellfish).ti,ab,kf.	2862	
21	((Marine or fish) adj2 extract).mp.	11	
22	(shark or sharks).ti,ab,kf.	107	
23	(samumed or SM04554).mp.	0	
24	Replicel.mp.	0	
25	(Electric adj2 Stimulation).ti,ab,kf.	87	
26	(Haircell or electrostimulation).mp.	35	
27	(follica or (hair adj2 follicle adj2 Neogenesis) or "FOL-004" or wounding).mp.	92	
28	((platelet adj2 rich adj2 (plasma* or fibrin*)) or ("platelet-rich" adj2 (fibrin or plasma*))).ti,ab,kf.	301	
29	(hair adj2 (autograft* or graft* or transplant*)).ti,ab,kf.	12	
30	or/9-29	10906	Hair loss medications, supplements, transplants
31	8 and 30	51	Base clinical set
32	((randomized or controlled or multicentre or multicenter) adj (trial or trials)).ti,ab,kf.	8874	
33	randomized.ab.	12443	
34	placebo.ab.	3502	
35	randomly.ab.	6098	

36	trial.ab.	14205	
37	groups.ab.	40718	
38	or/32-37	59679	Therapy filter
39	31 and 38	130	Therapy filter results

EMBASE

#	Searches	Results	Comment
1	alopecia/ or male type alopecia/	45106	
2	((androgenetica or androgenetic or androgenic or male or female) adj2 Alopecia*).ti,ab,kw.	3335	
3	hair loss/	13572	
4	(telogen adj2 effluvium).ti,ab,kw.	567	
5	(("Non scarring" or "non-scarring") adj2 alopecia*).ti,ab,kw.	276	
6	(hair adj2 (loss or thinning or plug or plugs)).ti,ab,kw.	11572	
7	(Baldness or Balding).ti,ab,kw.	1844	
8	("self-reported" adj2 thinning).ti,ab,kw.	1	
9	exp hair follicle/ and (injury or injuries).ti,ab,kw.	620	
10	or/1-9	60420	Alopecia terms
11	minoxidil/ or (minoxidil or alocutan or alopexy or alopexyl or alostil or aloxidil or "apo-gain" or crecisan or growell or "hair-treat" or hairgaine or hairgrow or headway or hebald or kapodin or kenacin or lacovin or locemix or "locion epc" or loniten or lonnoten or lonolox or lonoten or manoxidil or rogaine or minocutan or minodyl or minona or minorga or minotricon or minovital or "minoxi 5" or minoxicutan or minoxidil or minoximen or minoxitrim or minoxyl or moxidil or multigain or neocapil or neoxidil or nuhair or prexidil or regaine or regroe or regrou or regrowth or rehair or theroxidil or tiazolin or trefostil or unipexil or ylox).mp. or 38304-91-5.rn.	21142	
12	finasteride/ or (finasteride or alocare or andozac or "chibro proscar" or "chibro-proscar" or finastar or finastid or fincar or finired or finpro or fistrin or folians or frosst or genaprost or harifin or "l 652931" or l652931 or "mk 0906" or "mk 906" or mk0906 or mk906 or nasterol or pilus or "pro-cure" or prohair or propecia or propeshia or proscar or prosh or prostacare or prostacom or prostide or prostop or reprostom or symasteride or tensen or uromedin or "ym 152" or m152).mp. or 98319-26-7.rn.	15696	
13	Dutasteride/ or (dutaseride or advodart or avidart or avodart or avolve or duagen or "gg 745" or gg745 or "gi 198745" or "gi 198745x" or gi198745 or gi198745x).mp. or 164656-23-9.rn.	3031	
14	dutasteride plus tamsulosin/ or (combodart or duodart or jalyn or jelnu or juteo).mp.	128	
15	dutasteride plus testosterone/	5	
16	Low level light laser therapy/ or (low adj2 (level or light or intensity or energy or laser or power) adj2 (therap* or treat*)).ti,ab,kw.	6413	
17	Viviscal.mp.	13	
18	nutraceutical/	5469	
19	nutraceutical*.mp.	12453	
20	exp supplementation/	263577	
21	Nutrafol.mp.	6	
22	biotin/ or (biotin or "bio h tin" or bioepiderm or "bios ii" or biotine or "coenzyme r" or qizenday or "vitamin bw" or "vitamin h").mp. or 58-85-5.rn.	38481	
23	biotin derivative/	734	
24	biocytin/ or (biocytin or biotinyllysine or "n biotinyl l lysine").mp. or 576-19-2.rn.	1939	

25	"2 iminobiotin"/ or (iminobiotin or guanidinobiotin).mp. or 13395-35-2.rn. [****EMBASE since 2015****]	132	
26	exp collagen/po, tp, td [Oral Drug Administration, Topical Drug Administration, Transdermal Drug Administration]	236	
27	exp fish product/	2216	
28	((Marine or fish) adj2 extract).mp.	497	
29	shark/	4187	
30	shellfish/ or shellfish protein/	5617	
31	(samumed or SM04554).mp.	31	
32	Replicel.mp.	1	
33	electrostimulation/	67299	
34	(Haircell or electrostimulation).mp.	77705	
35	(follica or (hair adj2 follicle adj2 Neogenesis) or "FOL-004" or wounding).mp.	10155	
36	thrombocyte rich plasma/ or platelet-rich fibrin/ or (platelet adj2 rich adj2 plasma*).ti,ab,kw. or ("platelet-rich" adj2 plasma*).ti,ab,kw.	18894	
37	hair transplantation/ or (hair adj2 (autograft* or graft* or transplant*)).ti,ab,kw.	1781	
38	or/11-37	475887	Hair loss medications, supplements, transplants
39	10 and 38	6085	Base clinical set
40	randomized controlled trial/	665763	
41	Controlled clinical study/	463547	
42	random*.ti,ab.	1676923	
43	Randomization/	91335	
44	Intermethod comparison/	273774	
45	placebo*.ti,ab.	323213	
46	(compare or compared or comparison).ti.	522507	
47	((evaluated or evaluate or evaluating or assessed or assess) and (compare or compared or comparing or comparison)).ab.	2333287	
48	(open adj label).ti,ab.	89443	
49	((double or single or doubly or singly) adj (blind or blinded or blindly)).ti,ab.	239450	
50	double-blind procedure/	183270	
51	Parallel group$1.ti,ab.	27828	
52	(Crossover or cross over).ti,ab.	109304	
53	((assign* or match or matched or allocation) adj5 (alternate or group$1 or intervention$1 or patient$1 or subject$1 or participant$1)).ti,ab.	356604	
54	(assigned or allocated).ti,ab.	420570	
55	(controlled adj7 (study or design or trial)).ti,ab.	381039	
56	(volunteer or volunteers).ti,ab.	252598	
57	Human experiment/	548561	
58	Trial.ti.	329159	
59	or/40-58	5403014	
60	(Random$ adj sample$ adj7 ("cross section$" or questionnaire$1 or survey$1 or database$1)).ti,ab. not (comparative study/ or controlled study/ or randomi$ed controlled.ti,ab. or randomly assigned.ti,ab.)	6032	
61	Cross-sectional study/ not (randomized controlled study/ or controlled clinical study/ or controlled study/ or randomi?ed controlled.ti,ab. or control groups$1.ti,ab.)	280451	
62	(((case adj control$) and random$) not randomi?ed controlled).ti,ab.	18732	
63	(Systematic review not (trial or study)).ti.	182121	

64	(nonrandom$ not random$).ti,ab.	16894	
65	"random field$".ti,ab.	2533	
66	(random cluster adj3 sampl$).ti,ab.	1372	
67	(review.ab. and review.pt.) not trial.ti.	909888	
68	("we searched".ab. and review.ti.) or review.pt.	2744712	
69	"update review".ab.	119	
70	(databases adj4 searched).ab.	44360	
71	(rat or rats or mouse or mice or swine or porcine or murine or sheep or lambs or pigs or piglets or rabbit or rabbits or cat or cats or dog or dogs or cattle or bovine or monkey or monkeys or trout or marmoset$1).ti. and animal experiment/	1066398	
72	Animal experiment/ not (human experiment/ or human/)	2211214	
73	or/60-72	5398301	
74	59 not 73	4672820	Cochrane Therapy filter terms Box 3.e
75	39 and 74	901	Cochrane Therapy filter results
76	limit 39 to (randomized controlled trial or controlled clinical trial or multicenter study)	425	General Trials limit
77	75 or 76	957	Final results

Cochrane

ID	Search	Hits	Comment
#1	[mh alopecia]	682	
#2	((androgenetica or androgenetic or androgenic or male or female) NEAR/2 Alopecia*)	605	
#3	(telogen NEAR/2 effluvium)	35	
#4	(("Non scarring" or "non-scarring") NEAR/2 alopecia*)	8	
#5	(hair NEAR/2 (loss or thinning or plug or plugs))	1365	
#6	(balding or baldness)	225	
#7	("self-reported" NEAR/2 thinning)	2	
#8	MeSH descriptor: [Hair Follicle] this term only and with qualifier(s): [injuries - IN]	0	
#9	[mh ^"hair follicle"] and (injury or injuries)	3	
#10	{or #1-#9}	2147	Alopecia terms
#11	[mh minoxidil] or (minoxidil or alocutan or alopexy or alopexyl or alostil or aloxidil or "apo-gain" or crecisan or growell or "hair-treat" or hairgaine or hairgrow or headway or hebald or kapodin or kenacin or lacovin or locemix or "locion epc" or loniten or lonnoten or lonolox or lonoten or manoxidil or rogaine or minocutan or minodyl or minona or minorga or minotricon or minovital or "minoxi 5" or minoxicutan or minoxidil or minoximen or minoxitrim or minoxyl or moxidil or multigain or neocapil or neoxidil or nuhair or prexidil or regaine or regroe or regrou or regrowth or rehair or theroxidil or tiazolin or trefostil or unipexil or ylox)	1329	
#12	[mh finasteride] or (finasteride or alocare or andozac or "chibro proscar" or "chibro-proscar" or finastar or finastid or fincar or finired or finpro or fistrin or folians or frosst or genaprost or harifin or "l 652931" or l652931 or "mk 0906" or "mk 906" or mk0906 or mk906 or nasterol or pilus or "pro-cure" or prohair or propecia or propeshia or proscar or prosh or prostacare or prostacom or prostide or prostop	863	

	or reprostom or symasteride or tensen or uromedin or "ym 152" or m152)		
#13	[mh Dutasteride] or (dutaseride or advodart or avidart or avodart or avolve or duagen or "gg 745" or gg745 or "gi 198745" or "gi 198745x" or gi198745 or gi198745x)	255	
#14	[mh ^"Low-Level Light Therapy"]	1058	
#15	(low NEAR/2 (level or light or intensity or energy or laser or power) NEAR/2 (therap* or treat*)):ti,ab	2013	
#16	Viviscal	7	
#17	MeSH descriptor: [Dietary Supplements] explode all trees	13343	
#18	(nutraceutical* or supplement* or nutrafol)	95181	
#19	[mh biotin] or (biotin or "bio h tin" or bioepiderm or "bios ii" or biotine or "coenzyme r" or qizenday or "vitamin bw" or "vitamin h")	356	
#20	[mh biocytin] or (biocytin or biotinyllysine or "n biotinyl l lysine")	0	
#21	("2 iminobiotin" or iminobiotin or guanidinobiotin)	6	
#22	[mh Collagen] and ([mh "administration, oral"] or [mh "administration, buccal"] or [mh "administration, sublingual"] or [MH "administration, topical"] or [mh "administration, cutaneous"])	208	
#23	[mh ^seafood] or [mh ^"fish products"] or [mh shellfish]	193	
#24	((Marine or fish) NEAR/2 extract)	25	
#25	[mh sharks]	5	
#26	(samumed or SM04554)	39	
#27	replicel	0	
#28	[mh ^"Electric Stimulation Therapy"] or [mh ^"Electric Stimulation"]	3694	
#29	(Haircell or electrostimulation)	2117	
#30	(follica or (hair NEAR/2 follicle NEAR/2 Neogenesis) or "FOL-004" or wounding)	103	
#31	[mh ^"platelet-rich plasma"] or [mh ^"platelet-rich fibrin"] or (platelet NEAR/2 rich NEAR/2 plasma*) or ("platelet-rich" NEAR/2 plasma*)	2401	
#32	MeSH descriptor: [Hair] explode all trees and with qualifier(s): [transplantation - TR]	16	
#33	(hair NEAR/2 (autograft* or graft* or transplant*))	64	
#34	{or #11-#33}	109586	Hair loss medications, supplements, transplants
#35	#10 and #34	829	Base clinical set
